# Eremophilane Sesquiterpenes from a Deep Marine-Derived Fungus, *Aspergillus* sp. SCSIOW2, Cultivated in the Presence of Epigenetic Modifying Agents

**DOI:** 10.3390/molecules21040473

**Published:** 2016-04-18

**Authors:** Liyan Wang, Mengjie Li, Jianqiang Tang, Xiaofan Li

**Affiliations:** Shenzhen Key Laboratory of Marine Bioresource and Eco-environmental Science, Shenzhen Key Laboratory of Microbial Genetic Engineering, College of Life Sciences and Oceanography, Shenzhen University, Shenzhen 518060, China; lmengjie16@163.com (M.L.); tangjianqiang888@163.com (J.T.)

**Keywords:** marine fungus, eremophilane, epigenetic modification, ECD, nitric oxide inhibitory activity

## Abstract

Chemical epigenetic manipulation was applied to a deep marine-derived fungus, *Aspergillus* sp. SCSIOW2, resulting in significant changes of the secondary metabolites. Three new eremophilane-type sesquiterpenes, dihydrobipolaroxin B (**2**), dihydrobipolaroxin C (**3**), and dihydrobipolaroxin D (**4**), along with one known analogue, dihydrobipolaroxin (**1**), were isolated from the culture treated with a combination of histone deacetylase inhibitor (suberohydroxamic acid) and DNA methyltransferase inhibitor (5-azacytidine). **1**–**4** were not produced in the untreated cultures. **2** and **3** might be artificial because **1** could form **2** and **3** spontaneously in water by intracellular acetalization reaction. The absolute configurations of **1** and **2** were assigned based on ECD spectroscopy combined with time-dependent density functional theory calculations. All four compounds exhibited moderate nitric oxide inhibitory activities without cytotoxic effects.

## 1. Introduction

**Figure 1:**
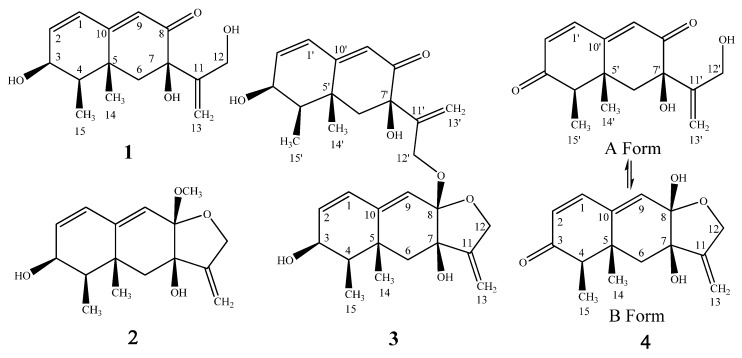
Structures of **1**, **2**, **3**, and **4**.

Marine fungi have attracted increasing attention as a source of structurally novel and biologically active secondary metabolites [1,2]. However, with the recent completion of fungal genomes, it has become clear that the number of gene clusters that encode secondary metabolites greatly outnumbers the characterized compounds from these organisms [3]. This observation suggests that many gene clusters generally remain unexpressed under a variety of laboratory culture conditions. Epigenetic modifying agents, primarily histone-modifying and DNA methylation-modifying agents, have been introduced as promising tools for manipulating the silent fungal genes for the purpose of discovering novel structures [4,5,6]. This approach has already been successfully applied to several marine-derived fungi to discover novel natural products using chemical epigenetic modifying agents, such as the histone deacetylase inhibitors suberohydroxamic acid (SBHA) [7,8] and sodium butyrate [9] and the DNA methyltransferase inhibitor 5-azacytidine (5-AZA) [10,11]. Moreover, studies on the effect of the concomitant addition of an HDAC inhibitor and a DNA methyltransferase inhibitor on fungal secondary metabolite production have been conducted. A marked increase was observed in the secondary metabolites produced by *Isaria tenuipes* and *Gibellula formosana* cultivated in the presence of both SBHA and RG108 (a DNA methyltransferase (DMNT) inhibitor) compared with those of fungi cultivated without inhibitors or with either SBHA or RG-108 [12,13]. In our efforts to identify novel structures and bioactive metabolites from deep sea-derived (over 1000 m) fungi, we found that the EtOAc extracts of *Aspergillus* sp. SCSIOW2 exhibited strong potency for inhibiting nitric oxide production without cytotoxic effects. A bioassay-guided chemical investigation resulted in the isolation of a novel cyclic dipeptide, cyclo-(l-*N*-MeTyr-anthranilic acid), which we have named 14-hydroxy-cyclopeptine [14]. To enhance the chemical diversity from this strain, the chemical epigenetic induction method was applied to the fermentation. We found that the cultivation of *Aspergillus* sp. SCSIOW2 with a combination of 1 mM SBHA, a competitive histone deacetylase (HDAC) inhibitor, and 1 mM 5-azacytidine (5-AZA), a DNMT inhibitor, resulted in a marked increase in the production of secondary metabolites compared with those of fungi cultivated without inhibitors, as indicated by LCMS and TLC analyses (Figure S1A–D). Therefore, the EtOAc extract of this culture was scaled-up and further separated using column chromatography and semi-preparative HPLC, resulting in the isolation of four eremophilane-type sesquiterpenes, dihydrobipolaroxin (**1**), dihydrobipolaroxin B (**2**), dihydrobipolaroxin C (**3**), and dihydrobipolaroxin D (**4**) (Figure 1). Compounds **1**–**4** were not produced in the untreated cultures (Figure S1). **1** is a known compound that was previously reported from *Bipolaris cynodontis*, a fungal pathogen of Bermuda grass [15], however, there is no ^13^C-NMR data and the absolute stereochemistry was not resolved. Here we reported the chemical epigenetic induction, production, isolation, and nitric oxide production inhibitory activities of these four compounds from a deep marine-derived fungus, *Aspergillus* sp. SCSIOW2.

## 2. Results

**Figure 2:**
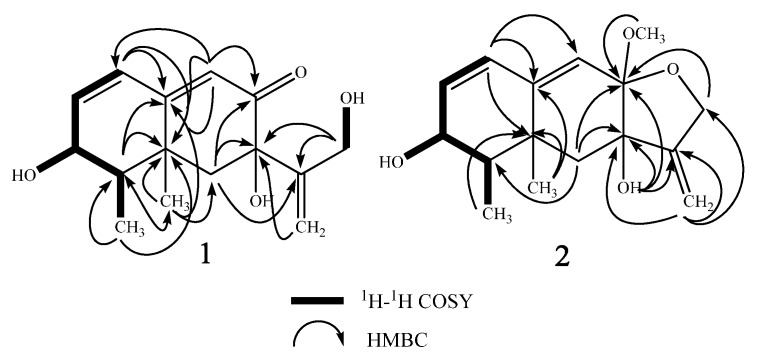
Key ^1^H-^1^H COSY and HMBC correlations of **1** and **2**.

**Figure 3:**
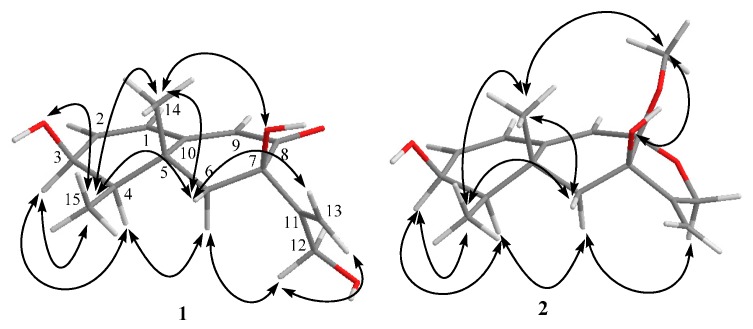
Key NOE correlations of **1** and **2**.

**Figure 4:**
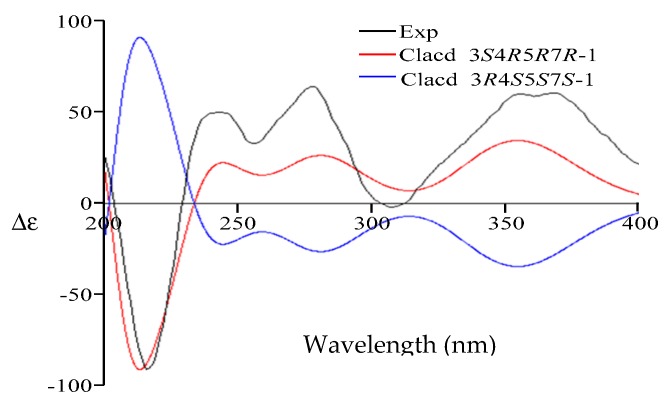
Comparison of the experimental ECD spectrum of **1** (black) with those calculated for the enantiomers 3*S*4*R*5*R*7*R* (red) and 3*R*4*S*5*S*7*S* (blue). (UV correction = −13 nm, bandwidth σ = 0.26 eV).

Compound **1** was isolated as a white amorphous powder. The structure of **1** was solved based on comprehensive NMR and HRMS analysis. Using HRESIMS, a molecular ion was measured at 265.1435 [M + H]^+^ (calcd. for C_15_H_21_O_4_, 265.1440), indicating a molecular formula of C_15_H_20_O_4_ with six degrees of unsaturation. The UV spectrum (MeOH) presented a single absorption maximum at λ_max_ (log ε) 281 (4.37) nm. The ^1^H- and ^13^C-NMR, DEPT, and HMQC spectroscopic data revealed a carbonyl carbon, three olefinic methines, an olefinic quaternary carbon, an exo-methylene group, an oxy-quaternary carbon, an oxymethine, an oxymethylene, an aliphatic methylene, two methyl groups, and three hydroxyl groups. The planar structure of **1** was determined to be dihydrobipolaroxin based on careful ^1^H-^1^H COSY and HMBC analyses (Table 1 and Figure 2) and comparison with the literature data [8]. Because the stereochemistry was not previously reported for this compound, NOESY correlations were utilized to determine the relative configuration of **1**. The axial methyl Me-14 exhibited correlations with 7-OH, Me-15, and H-6β. H-4 exhibited cross peaks with H-3 and H-6α; Me-15 exhibited cross peaks with Me-14 and H-6β; and Me-15 also exhibited cross peaks with H-3 and 3-OH with similar peak intensities; therefore, the α orientations of Me-14, Me-15, and 3-OH were established, while Me-15 was equatorial. Furthermore, one of the exo-methylene protons (H-13a) that resonated at δ_H_ 5.18 showed cross peaks with H-6β, while 7-OH presented correlations with Me-14 in the NOESY spectra. Thus, 7-OH was determined to be in a β orientation (Table 1, Figure 3). The relative configuration of **1** was thus established. A crystal for an XRD structure of **1** was unachievable due to the limited amount and the instability of the structure. The ECD spectrum of **1** was then recorded and compared with those calculated for each enantiomer using the time-dependent density functional theory (TDDFT) method. After conformation space analysis, 10 conformers were found for **1** (Table S1 shows the equilibrium populations of 10 stable conformations in methanol at the B3LYP/aug-cc-pVDZ level). Consequently, the calculated ECD spectrum for the enantiomer 3*S*4*R*5*R*7*R* showed a perfect fit with the experimental plot of **1**, which exhibited one negative and three positive Cotton effects at 219, 244, 279, and 356 nm, respectively (Figure 4). However, the calculated ECD spectrum of the 3*R*4*S*5*S*7*S* enantiomer was opposite to the experimental ECD data, with one positive and three negative Cotton effects at 210, 240, 282, and 356 nm, respectively (Figure 4). Hence, the stereochemistry of **1** was determined to be 3*S*4*R*5*R*7*R*.

**Figure 5:**
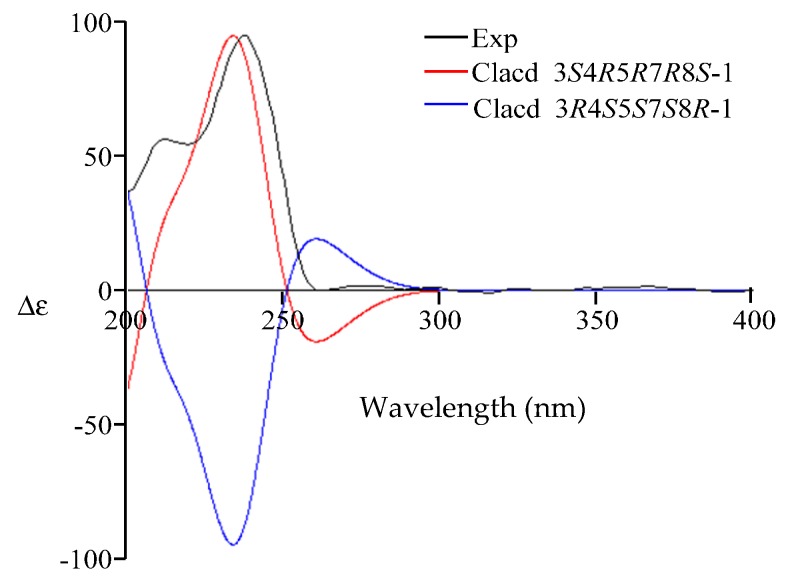
Comparison of the experimental ECD spectrum of **2** (black) with those calculated for the enantiomers 3*S*4*R*5*R*7*R*8*S* (red) and 3*R*4*S*5*S*7*S*8*R* (blue). (UV correction = 13 nm, bandwidth σ = 0.3 eV).

The HRESIMS of **2** presented a *m/z* of 279.1592 [M + H]^+^ (calcd. for C_16_H_23_O_4_, 279.1596), 14 units larger than **1**, which indicated an extra CH_2_ group. The UV maximum exhibited a short wavelength shift [λ_max_ (log ε) 238 (4.30) nm] compared with that of **1**. The ^1^H- and ^13^C-NMR were almost identical to those of **1**, except for three major differences: the absence of the resonance for the carbonyl moiety (C-8), which had been replaced by an acetal carbon (δ_C_ 101.4, q); the oxymethylene group (δ_H_ 3.82 d 4.2, H-12) changed to an AB quartet at δ_H_ 4.37 (d, 13.8) and δ_H_ 4.24 (d, 13.8), indicating a restrictive partial structure; and an additional methoxy group (δ_H_ 3.27 s, δ_C_ 48.1) (Table 2). These data strongly suggested an acetal group rather than a carbonyl group at C-8. The acetal structure at C-8 was further confirmed by HMBC correlations from the 8-methoxy and 12-H to the acetal carbon (C-8) (Table 2, Figure 2). Finally, careful ^1^H-^1^H COSY, HMQC, and HMBC analyses confirmed the structure as **2** (Table 2, Figure 2). The NOESY correlation between 8-methoxy and 14-Me indicated the β-orientation of the C-8 methoxy group. The NOE cross peak between H-12α and H-6α also supported the β-orientation of the C-8 methoxy because the distance between these two protons would be too far for an α-orientation. The other NOESY correlations were almost identical to those of **1**, indicating the same relative configurations (Table 2, Figure 3). Determining the absolute configuration of **2** was also attempted by ECD analysis. Only one conformer was found through confirmation space analysis because of the rigid structure of **2**. The calculated ECD spectrum for the enantiomer 3*S*4*R*5*R*7*R*8*S* exhibited a good fit with the experimental plot of **2**, which presented one major positive Cotton effect at 238 nm, whereas the calculated ECD spectrum of the 3*R*4*S*5*S*7*S*8*R* enantiomer was opposite to the experimental ECD data, with one negative Cotton effect at 230 nm (Figure 5). Thus, the 3*S*4*R*5*R*7*R*8*S* of **2** was determined.

The HRESIMS of **3** presented an *m/z* of 511.2704 [M + H]^+^ (calcd. for C_30_H_39_O_7_, 511.2696). The UV spectrum exhibited two maxima [λ_max_ (log ε) 239 (4.32), 283 (4.25) nm]. The ^1^H- and ^13^C-NMR spectra exhibited two sets of signals belonging to two bipolaroxin-type sesquiterpenes, respectively (Table 3). One set of signals is similar with those of **1**, possessing an 8′-carbonyl carbon (δ_C_ 196.7) and a free rotational 12′-oxymethylene group (δ_H_ 4.08 s, δ_C_ 61.4); the other set is almost identical with those of **2**, possessing a rigid five-membered ring and a C-8 acetal unit, except for the lack of a methoxy group. Careful 2D NMR analysis confirmed these two partial structures (Table 3). The HMBC correlation from the 12′ proton to the 8-acetal carbon unambiguously connected these two partial structures, and the structure was then characterized as **3**. NOE correlations of the two partial eremophilanes were almost identical with those of **1** and **2**, suggesting the same relative configurations. The only NOE correlation between these two partial structures is H-12′ with H-9, which did not provide us with much information about the stereochemistry of the C-8 linkage. However, the NOE cross peak between H-12α and H-6α convincingly indicated the β-orientation of the C-8 linkage.

**1** was found to be unstable in MeOH:H_2_O (9:1) solution when attempting crystallization one week after storage at room temperature, approximately 50% of **1** changed to **2** and **3** (Figure S2). Based on structures of **1**–**3**, the conversion was considered to be a typical acetalization reaction. The first step was nucleophilic addition of the α,β-unsaturated alcohol (12-OH) to the C-8 ketone; the second step was the addition of a methoxy group or 12-OH from another molecule to form **2** or **3**, respectively.

The HRESIMS of **4** presented a *m/z* of 263.1280 [M + H]^+^ (calcd. for C_15_H_19_O_4_, 263.1283), which is two units less than **1**, indicating a molecular formula of C_15_H_18_O_4_ with seven degrees of unsaturation. The ^1^H- and ^13^C-NMR spectra were quite similar to those of **3**, including two sets of bipolaroxin-type signals (Table 3). The major differences between **4** and **3** were the absence of two oxymethine groups, which were replaced by two carbonyl carbons (δ_C_ 200.3, 200.0) (Table 3). HMBC correlations between one carbonyl carbon at δ_C_ 200.3 with H1, H4, and Me-15 and the other one at δ_C_ 200.0 with H-1′, H-4′, and Me-15′ confirmed that the carbonyl carbons were located at the C-3 or C-3′ position. **3** was determined to be a dimer of **1**, connected with an acetal linkage at C-8; however, it was determined that **4** was a mixture of two compounds by careful 2D NMR analyses (Table 3). One compound (A form) is similar to **1**, containing a free rotational 12′-OH. The other compound (B form) is similar to **2**, containing a rigid five-membered ring with a hemiacetal structure. The A and B forms of **4** both contained 3 (or 3′)-carbonyl groups. NOESY correlations suggested the same relative configurations of **4** with those of **1**, **2**, and **3**. In the A form, H-4′ exhibited a cross peak with H-6′, Me-15′ exhibited cross peaks with Me-14′, H-6 β′, and OH-7′. Therefore, the α orientations of Me-14, Me-15, and OH-7 were established, while Me-15 was equatorial. In the B form, Me-14 exhibited a cross peak with OH-8, indicating the β orientation of the 8-hydroxyl group. The other NOE correlations were almost identical with those of the A form, suggesting the same relative configurations. The ^1^H-NMR integral area showed that the two distinct sets of signals can be observed in an approximately 3:5 (A:B, Figure S21) ratio in DMSO-*d*_6_. On standing in MeOD-*d*_4_, the equilibration changed to be 1:4 (A:B, Figure S27). The attempt to isolate the pure form of **4** failed due to the quick equilibration during the evaporation.

Great efforts have been conducted to purify **1**, too. HPLC analysis of purified **1** showed one clean peak (Figure S1A), however, it still contained minor impurities based on NMR spectra (Figures S3 and S4). The impurities might be generated from the same intramolecular acetalization to form an equilibria of compounds because those minor signals were very similar to **2** (Figures S3, S4, S9 and S10). However, we could not confirm the minor structure due to the low intensity of those signals.

**Figure 6:**
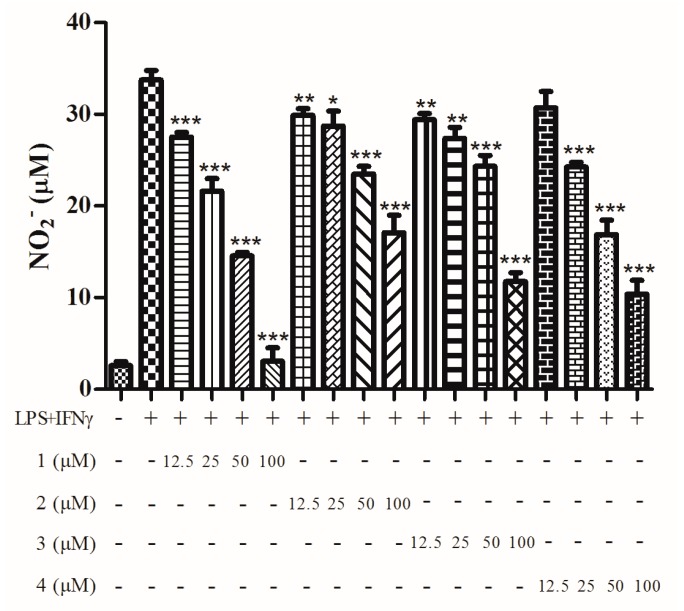
Effect of compounds **1**–**4** on inhibition of NO production stimulated by LPS and IFN-γ. The data were expressed as the means ± SD from four individual experiments and were analyzed using a *t* test to determine any significant differences. * *p* ≤ 0.05, ** *p* ≤ 0.01, *** *p* ≤ 0.001 compared to control group with LPS/IFN-γ.

**Figure 7:**
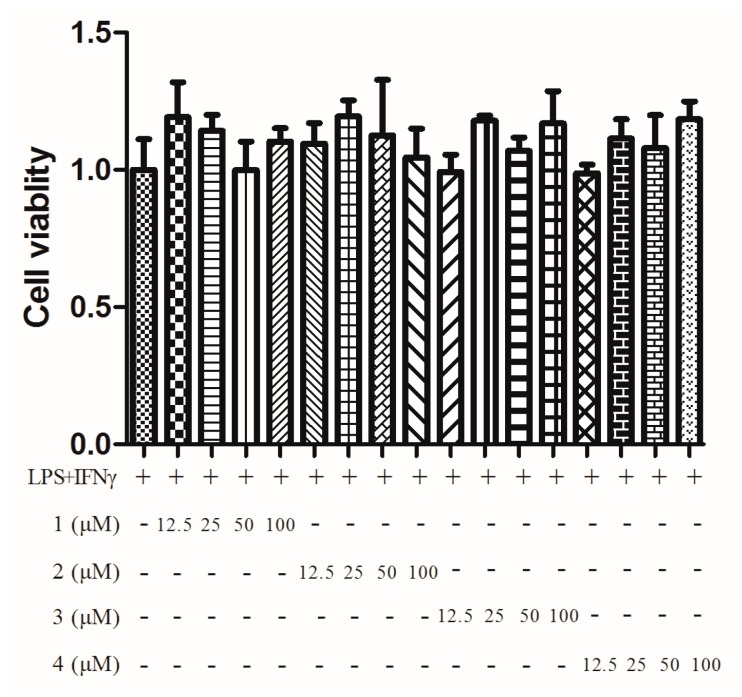
Cell viability determined by MTT assay. The experiment was performed four times, and the data are expressed as mean ± S.D. values.

We examined the inhibitory effects of **1**–**4** on the production of NO induced by LPS/INF-γ. **1**–**4** all exhibited NO production inhibitory activity with a dose dependent manner (Figure 6). The cytotoxic effects of **1**–**4** were measured using an MTT assay. None of the compounds exhibited any cytotoxic effects in the tested dose range (100–12.5 μM, Figure 7). It should be noticed that **1** contained minor impurities. The impurities could possibly be an equilibria form of **1** generated from intramolecular acetalization, which we could not confirm in this paper.

## 3. Discussion

Eremophilane type sesquiterpenes belong to bicyclic sesquiterpenes, containing only two complete isoprene subunits, are present in about 20 genera of the Asteraceae family, and can be regarded as the chemotaxonomic markers sources of *Ligularia*, *Senecio*, *Cacalia*, and *Petasites* genera [16]. They are also widely found from fungi species. These compounds are of interest because many of them showed a wide range of pharmaceutical functions including phytotoxic, cytotoxic, anti-bacterial, anti-microbial, anti-inflammatory, anti-allergic, and so on [16]. Macrophages play major roles in the immune responses by releasing various factors such as pro-inflammatory cytokines and nitrogen species. Excessive production of nitric oxide (NO) appears to associate with many chronic or acute diseases related to inflammation such as rheumatoid arthritis, cancer and even Alzheimer’s disease [17,18]. Inducible nitric oxide synthase (iNOS) is a key enzyme in the macrophage inflammatory response, which is the source of NO that is potently induced in response to proinflammatory stimuli. Activation interferon (IFN)-γ increases iNOS promoter activity and expression in response to lipopolysaccharide (LPS) *in vitro* [19,20]. Thus, we used LPS and IFN-γ activated Macrophage cells to evaluate NO production inhibitory activities. The cell viability was measured by MTT assay. Measurement of mitochondrial metabolic rate using MTT assay to indirectly reflect viable cell numbers has been widely applied. However, metabolic activity may be changed by different conditions or chemical treatments which can cause considerable variation in results reported from these assays [21]. Based on our knowledge, seven eremophilane type sesquiterpenes have been reported for their NO production inhibitory activities with the highest activity of 0.55 μg/mL (IC_50_), and 6-hydroxy group was considered critical for increasing the ability to inhibit NO production [22,23]. In our study, all four compounds (**1**–**4**) exhibited nitric oxide inhibitory activities in a dose dependent manner without cytotoxic effects. Although the activities were not as strong as reported components, these results showed that eremophilanes might have therapeutic benefits against various types of diseases by NO production inhibition potency.

## 4. Materials and Methods

### 4.1. General Experimental Procedures

Optical rotations were determined on a Jasco P-1020 polarimeter (Jasco, Tokyo, Japan). UV data were recorded on a Perkin Elmer Lambda 25 UV/Vis spectrometer (PerkinElmer, Boston, MA, USA). IR data were recorded using a Nicolet Avatar 330 FT-IR spectrometer (Thermo Scientific, Waltham, MA, USA). NMR spectra were acquired on a Bruker ASCEND 600 MHz NMR magnet system (Bruker, Ettlingen, Germany) using TMS as the internal standard. HR-ESIMS was performed using an AB SCIEX TOF/TOF™ 5800 system (AB Sciex, Redwood City, CA, USA). CD spectra were recorded on a Jasco J-815 CD Spectrometer (Jasco, Tokyo, Japan). Column chromatography was conducted using silica gel (100–200 mesh, Qingdao Marine Chemical Factory, Qingdao, China) and Sephadex LH-20 (Amersham Pharmacia Biotech, Piscataway, NJ, USA). TLC was performed on Merck TLC plates (silica gel 60 RP-18 F254S and silica gel 60 F254, Merck Millipore Corporation, Darmstadt, Germany), with compounds visualized by spraying with 5 % (*v*/*v*) H_2_SO_4_ in EtOH and then heating on a hot plate. HPLC was performed on a Shimadzu LC-20AT pump (Shimadzu Corporation, Tokyo, Japan) equipped with a SPD-20A UV-Vis detector and an Agilent Technologies 1260 Infinity series with a 1260 DAD detector. A YMC-Pack Ph column (4.6 × 250 mm, I.D. 5 μ), a YMC-Pack Pro C18 column (10 × 250 mm, I.D. 5 μ), and a YMC-Pack Pro C18 column (4.6 × 250 mm, I.D. 5 μ) were used for semi-preparative and analysis purposes, respectively.

### 4.2. Strain

Fungus SCSIOW2 was isolated from a deep marine sediment sample collected in the South China Sea (112°30.203E, 18°1.654N) at a depth of 2439 m. This fungus was characterized as *Aspergillus* sp. Based on the analysis of the ITS region sequence with GenebankS1. This fungus was deposited in the Marine Microbial Lab., College of Life Science, Shenzhen University (Shenzhen, China).

### 4.3. Fermentation, Extraction, and Isolation

Both seed and production media have the same constituents (2.0% glucose, 1.0% peptone, 0.5% yeast extract, 3.0% sea salt, with the pH adjusted to 7.5). *Aspergillus* sp. SCSIOW2 was cultured in 250 mL Erlenmeyer flasks containing 50 mL of seed medium. After growing at 28 °C, 220 rpm for two days, the cellular material was placed in a sterile Falcon tube and mixed by vortexing for several minutes to create a uniform fungal cell/spore suspension. Aliquots (5 mL) of seed cultures were inoculated into 160 mL of production medium in 1000 mL Erlenmeyer flasks. At the time of inoculation, 0.5 mL aliquots of DMSO-dissolved SBHA and water-dissolved 5-AZA were added in triplicate, resulting in final concentrations in the liquid media of 1 mM SBHA and 1 mM 5-AZA. Control group were added same amount of DMSO and water. The resulting cultures were fermented at 28 °C under static conditions for 15 days. The fermented broth of each flask was extracted three times with 250 mL of EtOAc. The EtOAc extracts of each condition were analyzed by reversed-phase LCMS on a YMC Pack pro ODS C18 column (4.6 × 150 mm I.D. 5 µ) eluted with MeOH–H_2_O (0:100–100:0 over 30 min, 1.0 mL/min). For preparative scale up, *Aspergillus* sp. SCSIOW2 was cultivated using 30 bottles of 1000 mL Erlenmeyer flasks containing 160 mL of production medium in the presence of both 1 mM SBHA and 1 mM 5-AZA. The combined EtOAc extract after evaporation (16.0 g) was applied to a Sephadex LH-20 column chromatograph (CC) with CHCl_3_–MeOH (1:1) to afford three fractions (Fr.1–Fr.3). Fr.2 (6.0 g) was further isolated by a silica gel CC, using gradient elution with hexane–EtOAc (95:5, 9:1; 8:2; 7:3), to afford 21 fractions (Fr.2-1–Fr.2-21). Fr.2-10 (62.0 mg), which was eluted with hexane–EtOAc (8:2), was further purified by HPLC with a YMC-Pack Ph column (4.6 × 250 mm I.D. 5 μ) eluted with MeOH–H_2_O (30:70–70:30 over 30 min, 0.8 mL/min) to yield dihydrobipolaroxin B (**2**) (1.8 mg, t_R_ 15.3 min). Fr.2-11 (142.9 mg), eluted with hexane–EtOAc (7:3), was purified by HPLC with a YMC-Pack Pro C18 column (4.6 × 250 mm I.D. 5 μ) eluted with MeCN–H_2_O (30:70–70:30 over 30 min, 1.0 mL/min) to yield dihydrobipolaroxin C (**3**) (1.0 mg, t_R_ 9.5 min). Fr.2-15 (70.0 mg), eluted with hexane–EtOAc (6:4), was purified by HPLC with a YMC-Pack Ph column (4.6 × 250 mm I.D. 5 μ) eluted with MeOH–H_2_O (0:100–100:0 over 30 min, 1.0 mL/min) to yield dihydrobipolaroxins (**1**) and (4) (3.5 mg and 1.2 mg, t_R_ 14.2 min and 16.6 min).

*Dihydrobipolaroxin* (**1**): white powder; ${\left[\alpha \right]}_{D}^{27}$ + 280 (*c* 1.0, MeOH); UV (MeOH) λ_max_ (log ε) 281 (4.37) nm; IR (film) ν_max_: 3407, 1651, 1626, 1455, 1304, 973 cm^−1^; ^1^H- and ^13^C-NMR see Table 1; HRESIMS *m/z* 265.1435 [M + H]^+^ (Calcd. for C_15_H_21_O_4_, 265.1440).

*Dihydrobipolaroxin B* (2): white powder; ${\left[\alpha \right]}_{D}^{27}$ + 240 (*c* 1.0, MeOH); UV (MeOH) λ_max_ (log ε) 238 (4.30) nm; IR (film) ν_max_: 3345, 3195, 2722, 1455, 1304, 808 cm^−1^; ^1^H- and ^13^C-NMR see Table 2; HRESIMS *m/z* 279.1592 [M + H]^+^ (Calcd. for C_16_H_23_O_4_, 279.1596).

*Dihydrobipolaroxin C* (**3**): white powder; ${\left[\alpha \right]}_{D}^{27}$ + 255 (*c* 0.5, MeOH); UV (MeOH) λ_max_ (log ε) 239 (4.32), 283 (4.25) nm; IR (film) ν_max_: 3410, 3196, 1621, 1457, 1304, 840 cm^−1^; ^1^H- and ^13^C-NMR see Table 3; HRESIMS *m/z* 511.2704 [M + H]^+^ (Calcd. for C_30_H_39_O_7_, 511.2696).

*Dihydrobipolaroxin D* (**4**): white powder; ${\left[\alpha \right]}_{D}^{27}$ + 50 (*c* 1.2, MeOH); UV (MeOH) λ_max_ (log ε) 280 (3.95) nm; IR (film) ν_max_: 3346, 3195,1675, 1591, 1304, 808 cm^−1^; ^1^H- and ^13^C-NMR see Table 3; HRESIMS *m/z* 263.1280 [M + H]^+^ (Calcd for C_15_H_19_O_4_, 263.1283).

### 4.4. Quantum Chemical ECD Calculations

In this study, we used the ECD calculation protocol proposed by Nugroho and Morita [24]. The initial 3D structures of the molecules were prepared using Chem3D and minimized with the MMFF94S force field implemented in Chem3D. After the initial structure was further optimized with XedMin in default mode, the conformation space was sampled using XedeX with an energy window of 5 kcal·mol^−1^ above the ground state and RMSD 0.8 to remove duplicated conformers [25]. Then, each conformer was optimized and verified as true minima of the potential energy surface using Gaussian 09 with the DFT method at the B3LYP/aug-cc-pVDZ level [26]. The polarizable continuum model (IEFPCM) was used to take the solvent effects of methanol into account. The optimized conformers were further used to perform a TDDFT calculation at the B3LYP/aug-cc-pVDZ level with the polarizable-conductor calculation model (IEFPCM, methanol as the solvent). In each TDDFT calculation, the 100 lowest electronic transitions were calculated for each conformer. The ECD spectra and overall ECD spectra (weighted by Boltzmann statistics) and comparison of the experimental and calculated spectra were performed using the software SpecDis [27,28].

### 4.5. Cell Culture Condition

Mouse RAW264.7 macrophage cells purchased from the American Type Culture Collection (ATCC, Manassas, VA, USA) , were cultured in RPMI 1640 medium supplemented with 10 % heat-inactivated fetal bovine serum (Gibco) at 37 °C in a humidified incubator with 5 % CO_2_ and 95 % air. The medium was routinely changed every two days. RAW 264.7 cells were passaged by trypsinization until they attained confluence.

### 4.6. Nitric Oxide Inhibitory Assay

The cells were plated in a 96-well plate at a density of 1.0 × 10^5^ cells/well and grown for 2 h to allow the cells to attach to the plate. The tested samples were dissolved in DMSO, and then two-fold serial dilutions were performed in RPMI-1640 (Thermo Fisher Scientific, Carlsbad, CA, USA). The final concentrations of samples **1**–**4** in the culture medium were 100, 50, 25, and 12.5 µM. The samples were added to the culture simultaneously with both *Escherichia coli* LPS (1.5 µg/mL, Sigma, St. Louis, MO, USA) and recombinant mouse IFN-γ (10 ng/mL, PeproTech, Rocky Hill, NJ, USA). Then, the cells were incubated at 37 °C for approximately 24 h and subsequently chilled on ice. Subsequently, 100 µL of the culture supernatant was placed in duplicate in the wells of 96-well plates. To quantify nitrite, 50 µL of Griess reagent (1% sulfanilamide in 5% H_2_PO_4_ and 0.1% *N*-1-naphthyletylenediamide dihydrochloride) was added to each well. After 10 min, the reaction products were colorimetrically quantified at 550 nm using a microplate reader (BIO-RAD, Hercules, CA, USA). The concentrations of nitrite were calculated by using a standard calibration curve [29,30].

### 4.7. MTT Assay

Cell viability was determined using an MTT assay. After 24 h of incubation with or without test samples, the old medium was replaced with 100 µL of fresh culture medium, and 10 µL of the 12 mM MTT stock solution was then added to each well. The cells were cultured at 37 °C for 4 h, the supernatant was removed, and then 50 µL of DMSO was added to each well and mixed thoroughly. The cells were further incubated at 37 °C for 10 min. The reaction products were quantified at 540 nm using a microplate reader (BIO-RAD). The untreated cells were considered as having 100% of viable cells. Results are expressed as percentage of viable cells when compared with the control group [31].

### 4.8. Statistical Analysis

All results were expressed as mean±SD. Statistical comparison was conducted using Student’s *t* test. The results were considered to be significant when *p* < 0.05.

## 5. Conclusions

In conclusion, the marine-derived fungal strain *Aspergillus* sp. SCSIOW2 was effectively induced by epigenetic modifying agents to produce novel compounds. Three new eremophilane-type sesquiterpenes, dihydrobipolaroxin B (2), dihydrobipolaroxin C (3), and dihydrobipolaroxin D (4), along with one known analogue, dihydrobipolaroxin (1), were isolated from the culture treated with a combination of SBHA and 5-AZA. The eremophilane sesquiterpenes were produced by *Aspergillus* sp. SCSIOW2 only when treated with chemical epigenetic modifiers. This result suggests that the combination of HDAC and DNMT inhibitors can be used to identify diverse natural products hidden in silent biosynthetic pathways from marine fungi. The structures of all compounds were determined on the basis of spectroscopic data. Compound 1 was previously reported from *Bipolaris cynodontis*, a fungal pathogen of Bermuda grass, as a minor constituent, along with a major compound, bipolaroxin [15]. Those authors did not assign the absolute conguration, neither the bioactivity, possibly due to paucity of material. In our study, the structures of 1–4 were able to determin by comprehensive NMR and HRMS analysis. The absolute configurations of 1 and 2 were assigned based on ECD spectroscopy combined with time-dependent density functional theory calculations. In addition, 1 was found unstable in solvent to form 2 and 3 by intracellular acetalization reaction between 12-OH (α,β-unsaturated alcohol) and the C-8 ketone. Thus 2 and 3 might be artificial products. 4 was found to be a mixture of two equilibrium structures in solution formed by the same acetalization reaction, the ^1^H-NMR integral area showed the two distinct sets of signals can be observed in an approximately 3:5 (A:B) ratio in DMSO-*d*_6_ and 1:4 in MeOD-*d*_4_. 1 contains minor impurities too. The impurities of 1 might be generated by the same reaction, however, the signals from impurities were too small to do further determination.

## Figures and Tables

**Table 1:** NMR spectroscopic data for compound **1** (DMSO-*d*_6_) ^a^.

Position	δ_H_ (Mult, *J* in Hz) ^b^	δ_C_^c^	^1^H-^1^H COSY	HMBC	NOESY
1	6.26 m ^d^	127.4	2	C-2,3,5,9,10	2,9
2	6.24 m ^d^	139.0	1,3	C-3,4,10	1
3	4.03 m	66.0	2,4,3-OH	C-1,2	4, 15
4	1.57 m	41.8	3,15	C-5,10,14,15	3,6α
5		35.9			
6α	1.86 d (14.4)	46.1	6β	C-4,5,7,8,10,11,14	4,12
6β	1.91 d (14.4)		6α	C-4,5,7,8,10,11,14	14,15,13a
7		75.9			
8		196.9			
9	5.76 s	122.9		C-1,5,7,10	1
10		163.0			
11		153.7			
12	3.82 d (4.2) (2H)	60.9	12-OH	C-7,11,13	6α, 13b
13a	5.18 d (1.8)	109.3	13b	C-7,11,12	6β
13b	5.21 d (1.8)		13a	C-7,11,12	12, 12-OH
14	1.35 s	22.6		C-4,5,6,10	6β,7-OH,15
15	0.99 d (6.6)	10.7	4	C-3,4,5	3,3-OH,6β,14
3-OH	4.88 d (5.4)		3	C-2,3,4	15
7-OH	5.30 s			C-6,7	14
12-OH	4.37 t (4.2)		12	C-11,12	13b

^a^ Chemical shifts (δ) in ppm; ^b^ 600 MHz; ^c^ 150 MHz; ^d^ overlapped signal.

**Table 2:** NMR spectroscopic data for compound **2** (DMSO-*d*_6_) ^a^.

Position	δ_H_ (Mult, *J* in Hz) ^b^	δ_C_^c^	^1^H-^1^H COSY	HMBC	NOESY
1	6.05 d (9.6)	128.0	2	C-3,5,9,10	2,9
2	5.85 dd (9.6, 4.8)	132.2	1,3	C-1,3,4,10	1
3	3.89 m	66.6	2,4,3-OH	C-1,2,4,5,15	4,15
4	1.36 m	43.1	3,15	C-3,5,10,14,15	3
5		35.9			
6α	1.32 d (13.8)	44.7	6β	C-5,7,8,10,11,14,	4,12
6β	1.68 d (13.8)		6α		14,15,13a
7		78.6			
8		101.4			
9	5.72 s	117.4		C-1,5,7,10	1,8-OCH_3_
10		144.9			
11		154.7			
12α	4.37 d (13.8)	66.7	12β	C-7,8,11,13	6α,13b
12β	4.24 d (13.8)		12α		13b
13a	5.12 s	104.2	13b	C-7,11,12	6β
13b	4.92 s		13a		12α,12β
14	1.25 s	20.8		C-4,5,6,10	6β,7-OH,8-OCH_3_,15
15	0.94 d (6.6)	10.6	4	C-3,4,5	3,3-OH,6β,14
3-OH	4.59 d (4.2)		3	C-2,3,4	15
7-OH	4.24 s			C-6,7,8,11	14
8-OCH_3_	3.27 s	48.1		C-8	9,7-OH,14

^a^ Chemical shifts (δ) in ppm; ^b^ 600 MHz; ^c^ 150 MHz.

**Table 3:** NMR spectroscopic data for compounds **3** and **4** (DMSO-*d*_6_) ^a^.

No.	3			4		
	δ_H_ (Mult, *J* in Hz) ^b^	δ_C_^c^	HMBC	δ_H_ (Mult, *J* in Hz) ^b^	δ_C_^c^	HMBC
1	5.99 d (9.6)	127.8	C-2,3,5,9,10	7.11 d (9.6)	144.3	C-2,3,9,10
2	5.84 dd (9.6,4.8)	132.3	C-1,3,4,10	5.93 d (9.6)	127.2	C-1,4,10
3	3.88 m	66.5	C-1,2,4,5		200.3	
4	1.32 m	43.0	C-5,6,10,14,15	2.34 m	52.2	C-3,5,6,14,15
5		35.8			39.1	
6α	1.30 d (13.8)	44.3	C-4,5,14	1.62 d (13.8)	44.1	C-4,5,7,8
6β	1.69 d (13.8)			1.76 d (13.8)		10,11,14
7		78.7			76.2	
8		101.6			98.6	
9	5.61 s	117.7	C-1,5,7,8,10	6.01 s	130.8	C-1,5,7,8
10		144.8			140.8	
11		154.2			154.3	
12	4.37 d (13.8)	67.0	C-8,11,13	4.35 d (14.4) ^d^	66.2	C-7,8,11,13
12	4.24 d (13.8)			4.40 d (14.4) ^d^		
13a	5.12 brs	104.5	C-7,11	5.18 s	104.9	C-7,11,12
13b	4.89 brs ^d^			4.99 s		
14	1.22 s	20.8	C-4,5,6	1.11 s	19.95	C-4,5,6,10
15	0.92 d (7.2)	10.6	C-3,4,5	0.86 d (6.6)	7.09	C-3,4,5
1′	6.26 m ^d^	139.0	C-3′,5′,9′,10′	7.30 d (10.2)	142.6	C-3′,5′,9′,10′
2′	6.25 m ^d^	127.4	C-1′,3′,4′, 10′	6.22 d (10.2) ^d^	131.5	C-1′,4′,10′
3′	3.96 m	66.1			200.0	
4′	1.57 m	41.8	C-5′,6′,10′,14′,15′	2.70 m	51.9	C-3′,5′,6′,10′
5′		35.9			40.0	14′,15′
6′α	1.91 s (2H)	45.5	C-7′,8′,10′	2.28 d (14.4)	45.4	C-5′,7′,8′
6′β			11′,14′	1.92 d (14.4)		10′,14′
7′		75.8			75.1	
8′		196.7			196.4	
9′	5.74 s	122.9	C-5′,7′,10′	6.22 s ^d^	127.5	C-1′,5′,7′,10′
10′		163.2			158.9	
11′		149.6			152.7	
12′	4.08 s (2H)	61.4	C-8,6′,11′,13′	3.90 d (4.8)	61.0	C-7′,11′,13′
13′a	5.28 brs (2H)	112.5	C-7′,11′,12′	5.26 s	110.0	C-7′,11′,12′
13′b				5.23 s		
14′	1.22 s	22.6	C-6′	1.23 s	21.8	C-4′,5′,6′,10′
15′	0.92 d (7.2)	10.7	C-3′,4′,5′	0.98 d (6.6)	7.09	C-3′,4′,5′
3-OH	4.60 d (5.4)		C-2,3,4			
7-OH	3.59 d (1.2)		C-6,7,8	6.54 s		C-7,8
8-OH				4.36 s		C-7,8,9
3′-OH	4.88 d (6.6) ^d^		C-3′,4′			
7′-OH	5.39 s		C-11′	5.60 s		C-6′,7′,8′
12′-OH				4.81 t (4.8)		C-12′

^a^ Chemical shifts (δ) in ppm; ^b^ 600 MHz; ^c^ 150 MHz; ^d^ overlapped signal.

## Supplementary Materials

Supplementary materials can be accessed at:  http://www.mdpi.com/1420-3049/21/4/473/s1.

## References

[1] Newman D.J., Cragg G.M. (2012). Natural products as sources of new drugs over the 30 years from 1981 to 2010. J. Nat. Prod..

[2] Blunt J.W., Copp B.R., Keyzers R.A., Munro M.H.G., Prinsep M.R. (2012). Marine natural products. Nat. Prod. Rep..

[3] Scherlach K., Hertweck C. (2009). Triggering cryptic natural product biosynthesis in microorganisms. Org. Biomol. Chem..

[4] Shwab E.K., Bok J.W., Tribus M., Galehr J., Graessle S., Keller N.P. (2007). Histone deacetylase activity regulates chemical diversity in *Aspergillus*. Eukaryot. Cell.

[5] Cichewicz R.H. (2010). Epigenome manipulation as a pathway to new natural product scaffolds and their congeners. Nat. Prod. Rep..

[6] VanderMolen K.M., Darveaux B.A., Chen W.L., Swanson S.M., Pearce C.J., Oberlies N.H. (2014). Epigenetic manipulation of a filamentous fungus by the proteasome-inhibitor bortezomib induces the production of an additional secondary metabolite. RSC Adv..

[7] Wiliams R.B., Henrikson J.C., Hoover A.R., Lee A.E., Cichewicz R.H. (2008). Epigenetic remodeling of the fungal secondary metabolome. Org. Biomol. Chem..

[8] Vervoort H.C., Draskovic M., Crews P. (2011). A novel dipeptide from a hawaiian marine sediment-derived fungus, *Aspergillus insulicola*. Org. Lett..

[9] Zhang W., Shao C.L., Chen M., Liu Q.A., Wang C.Y. (2014). Brominated resorcylic acid lactones from the marine-derived fungus *Cochliobolus lunatus* induced by histone deacetylase inhibitors. Tetrahedron Lett..

[10] Wang X.R., Sena Filho J.G., Hoover A.R., King J.B., Ellis T.K., Powell D.R., Cichewicz R.H. (2010). Chemical epigenetics alters the secondary metabolite composition of guttate excreted by an atlantic-forest-soil-derived *Penicillium citreonigrum*. J. Nat. Prod..

[11] Chung Y.M., Wei C.K., Chuang D.W., El-Shazly M., Hsieh C.T., Asai T., Oshima Y., Hsieh T.J., Hwang T.L., Wu Y.C. (2013). An epigenetic modifier enhances the production of anti-diabetic and anti-inflammatory sesquiterpenoids from *Aspergillus sydowii*. Bioorg. Med. Chem..

[12] Asai T., Chung Y.M., Sakurai H., Ozeki T., Chang F.R., Yamashita K., Oshima Y. (2012). Aromatic polyketide production in *Cordyceps indigotica*, an entomopathogenic fungus, induced by exposure to a histone deacetylase inhibitor. Org. Lett..

[13] Asai T., Chung Y.M., Sakurai H., Ozeki T., Chang F.R., Wu Y.C., Yamashita K., Oshima Y. (2012). Highly oxidized ergosterols and isariotin analogs from an entomopathogenic fungus, *Gibellula formosana*, cultivated in the presence of epigenetic modifying agents. Tetrahedron.

[14] Zhou X., Fang P.Y., Tang J.Q., Wu Z.Q., Li X.F., Li S.M., Wang Y., Liu G., He Z.D., Gou D.M. (2015). A novel cyclic dipeptide from deep marine-derived fungus *Aspergillus* sp. SCSIOW2. Nat. Prod. Res..

[15] Sugawara F., Strobel G., Fisher L., Van Duyne G.D., Clardy J. (1985). Bipolaroxin, a selective phytotoxin produced by *Bipolaris cynodontis*. Proc. Natl. Acad. Sci. USA.

[16] Hou C.J., Kulka M., Zhang J.P., Li Y.M., Guo F.J. (2014). Occurrence and biological activities of eremophilane-type Sesquiterpenes. Mini-Rev. Midecinal Chem..

[17] Aktan F., Henness S., Roufogalis B.D., Ammit A.J. (2003). Gypenosides derived from *Gynostemma pentaphyllum* suppress NO synthesis in murine macrophages by inhibiting iNOS enzymatic activity and attenuating NF-κB-mediated iNOS protein expression. Nitric Oxide.

[18] Bogdan C. (2001). Nitric oxide and the immune response. Nat. Immunol..

[19] Lorsbach R.B., Murphy W.J., Lowenstein C.J., Snyder S.H., Russell W. (1993). Expression of the nitric oxide synthase gene in mouse macrophages activated for tumor cell killing. Molecular basis for the synergy between interferon-gamma and lipopolysaccharide. J. Biol. Chem..

[20] Tötemeyer S., Sheppard M., Lloyd A., Roper D., Dowson C., Underhill D., Murray P., Maskell D., Bryant C. (2006). IFN-γ enhances production of nitric oxide from macrophages via a mechanism that depends on nucleotide oligomerization domain-2. J. Immunol..

[21] Van Meerloo J., Kaspers G.J., Cloos J. (2011). Cell sensitivity assays: The MTT assay. Methods Mol. Biol..

[22] Hwang B.Y., Lee J.H., Koo T.H., Kim H.S., Hong Y.S., Ro J.S., Lee K.S., Lee J.J. (2002). Furanoligularenone, an eremophilane from *Ligularia fischeri*, inhibits the LPS-induced production of nitric oxide and prostaglandin E2 in macrophage RAW264.7 cells. Planta Med..

[23] Zhao J.H., Shen T., Yang X., Zhao H., Li X., Xie W.D. (2012). Sesquiterpenoids from Farfugium japonicum and their inhibitory activity on NO production in RAW264.7 Cells. Arch. Pharmacal Res..

[24] Nugroho E.A., Morita H. (2014). Circular dichroism calculation for natural products. J. Nat. Med..

[25] Cresset. http://www.cresset-group.com.

[26] Frisch M.J., Trucks G.W., Schlegel H.B., Scuseria G.E., Robb M.A., Cheeseman J.R., Scalmani G., Barone V., Mennucci B., Petersson G.A. (2013). Gaussian 09, Revision D.01.

[27] Bruhn T., Schaumlöffel A., Hemberger Y., Bringmann G. (2013). SpecDis, Version 1.60.

[28] Bruhn T., Schaumlöffel A., Hemberger Y., Bringmann G. (2013). SpecDis: Quantifying the comparison of calculated and experimental electronic circular dichroism spectra. Chirality.

[29] Zhao F., Wang L., Liu K. (2009). *In vitro* anti-inflammatory effects of arctigenin, a lignan from Arctium lappa L., through inhibiton on iNOS pathway. J. Ethnopharmacol..

[30] Li L., Wang L.Y., Wu Z.Q., Yao L.J., Wu Y.H., Huang L., Liu K., Zhou X., Gou D.M. (2014). Anthocyanin-rich fractions from red raspberries attenuate inflammation in both RAW264.7 macrophages and a mouse model of colitis. Sci. Rep..

[31] Denizot F., Lang R. (1986). Rapid colorimetric assay for cell growth and survival. Modifications to the tetrazolium dye proceduregiving improved sensitivity and reliability. J. Immunol. Methods.

