# Supplementary material for: Eremophilane Sesquiterpenes from a Deep Marine-Derived Fungus, Aspergillus sp. SCSIOW2, Cultivated in the Presence of Epigenetic Modifying Agents
Source: Molecules. 2016 Apr 18;21(4):473. doi: 10.3390/molecules21040473 (PMC6274295; doi:10.3390/molecules21040473)
Supplement: Supplementary file 1 [file molecules-21-00473-s001.pdf]

Supplementary Materials: Eremophilane Sesquiterpenes from a Deep Marine-Derived Fungus, *Aspergillus* sp. SCSIOW2, Cultivated in the Presence of Epigenetic Modifying Agents

Liyan Wang, Mengjie Li, Jianqiang Tang and Xiaofan Li

Table S1. Conformer distribution of 1 in solvated models (methanol) calculation at the B3LYP/aug-cc-PVDZ level.

Conformers	Contribution %
1	26.05
2	19.17
3	19.17
4	14.77
5	6.69
6	5.01
7	4.03
8	2.25
9	2.25
10	0.61

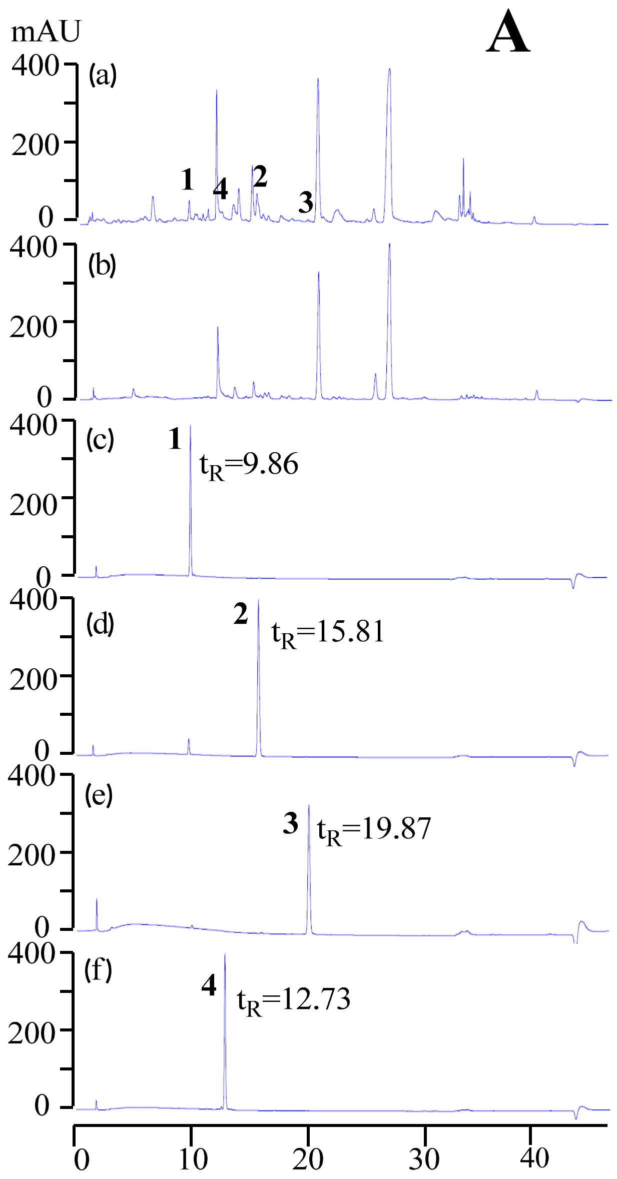

Figure S1. *Cont.*

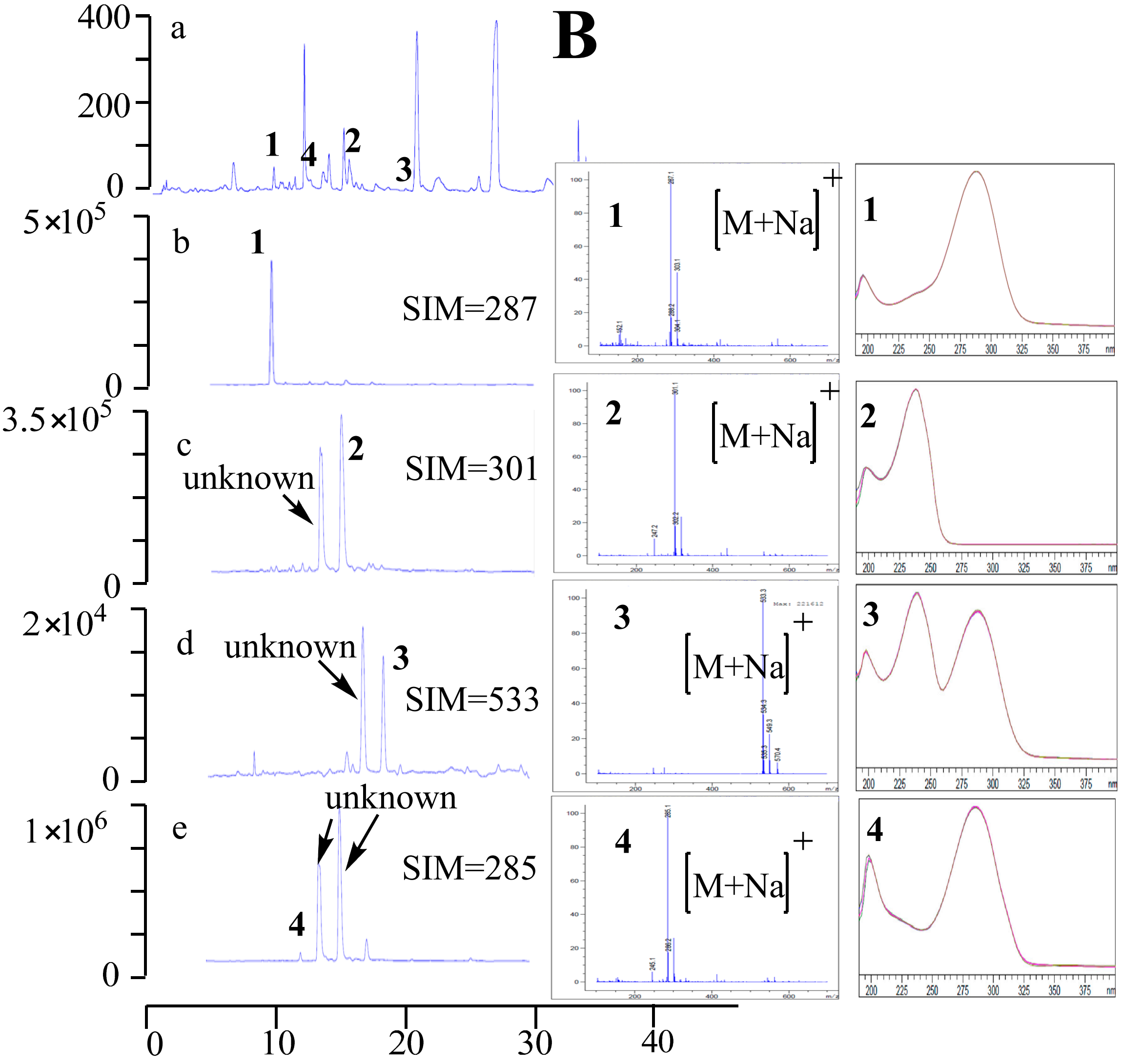

Figure S1. *Cont.*

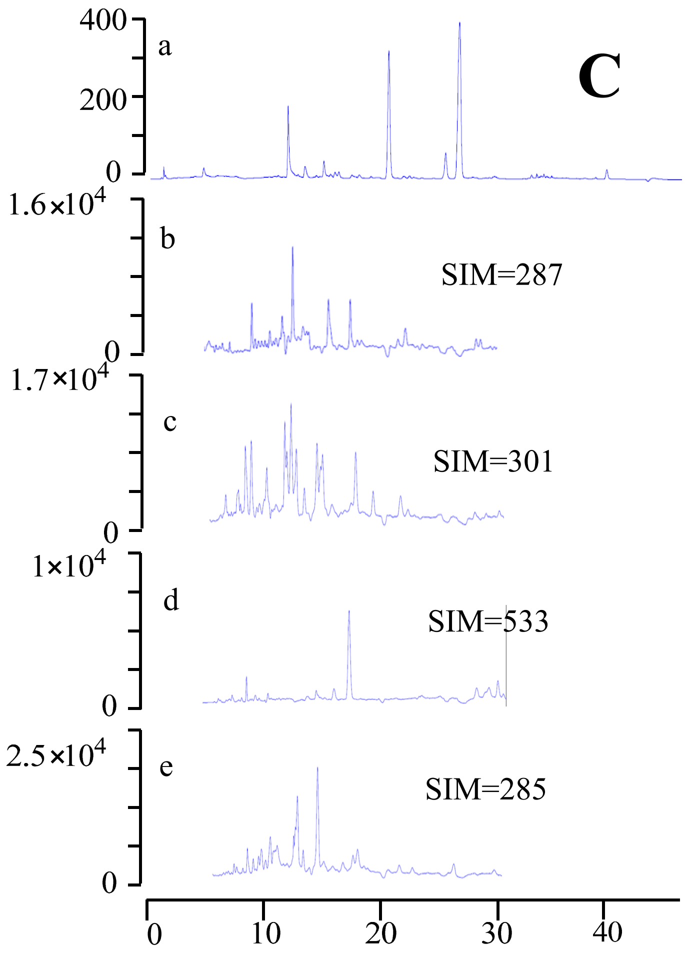

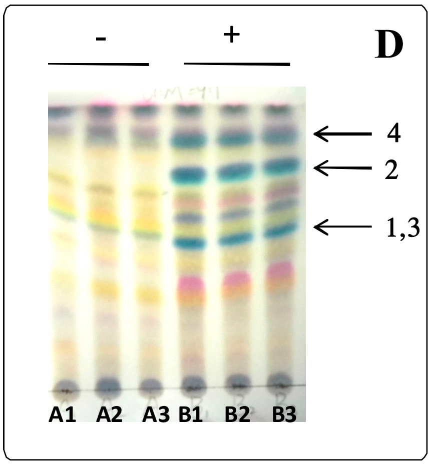

Figure S1. Effects of SBHA and 5-AZA on secondary metabolites (SMs) production by *Aspergillus* sp. SCSIOW2. SMs were extracted from one week cultures with (+) or without (−) 1 mM SBHA and 1 mM 5-AZA. (**A**) HPLC analyses [(a) = +; (b) = −; (c–f) = standards of 1–4]; Effects of SBHA and 5-AZA on secondary metabolites (SMs) production by *Aspergillus* sp. SCSIOW2. SMs were extracted from one week cultures with (+) or without (−) 1 mM SBHA and 1 mM 5-AZA. (**B**) LCMS analyses of samples with epigenetic modifiers. (a) = HPLC of +; (b–e) = SIM287, 301, 533, 285; Effects of SBHA and 5-AZA on secondary metabolites (SMs) production by *Aspergillus* sp. SCSIOW2. SMs were extracted from one week cultures with (+) or without (−) 1 mM SBHA and 1mM 5-AZA. (**C**) LCMS analyses of samples without epigenetic modifiers. (a) = HPLC of −; (b–e) = SIM287, 301, 533, 285; Effects of SBHA and 5-AZA on secondary metabolites (SMs) production by *Aspergillus* sp. SCSIOW2. SMs were extracted from one week cultures with (+) or without (−) 1 mM SBHA and 1mM 5-AZA. (**D**) TLC analysis.

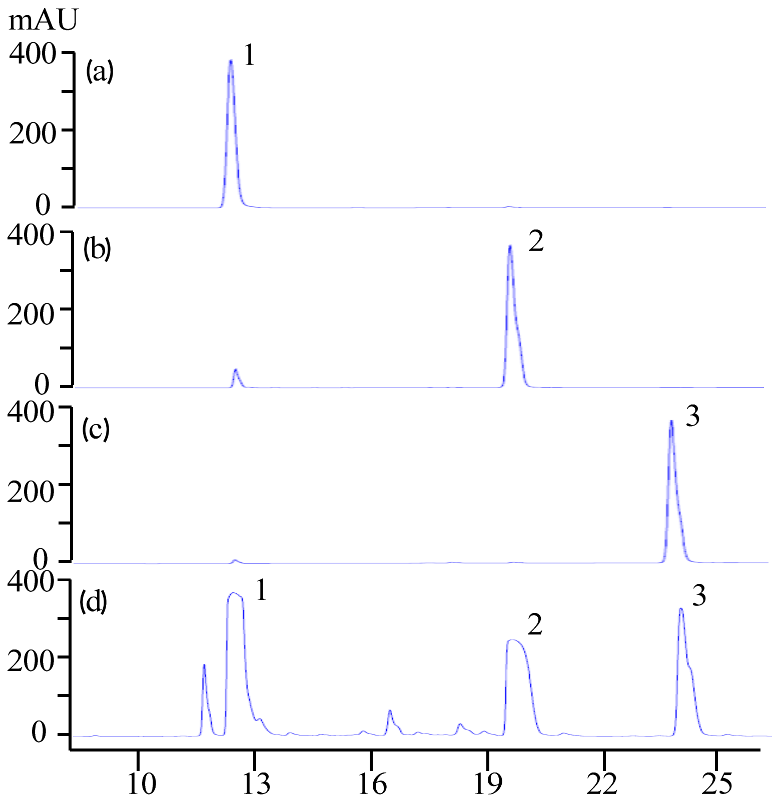

Figure S2. HPLC analysis of **1** (**a**), **2** (**b**) and **3** (**c**), and profile of **1** changed to **2** and **3** after storage for 1 week in MeOH:H_2O (9:1) solution (**d**); HPLC was performed with a YMC Pack pro ODS C18 column (4.6 × 250 mm I.D. 5 μ) eluted with MeOH-H_2O (0:100–50:50 from 0 to 10 min; 50:50–80:20 from 10 to 20 min; 1.0 mL/min) with detection at 254 nm.

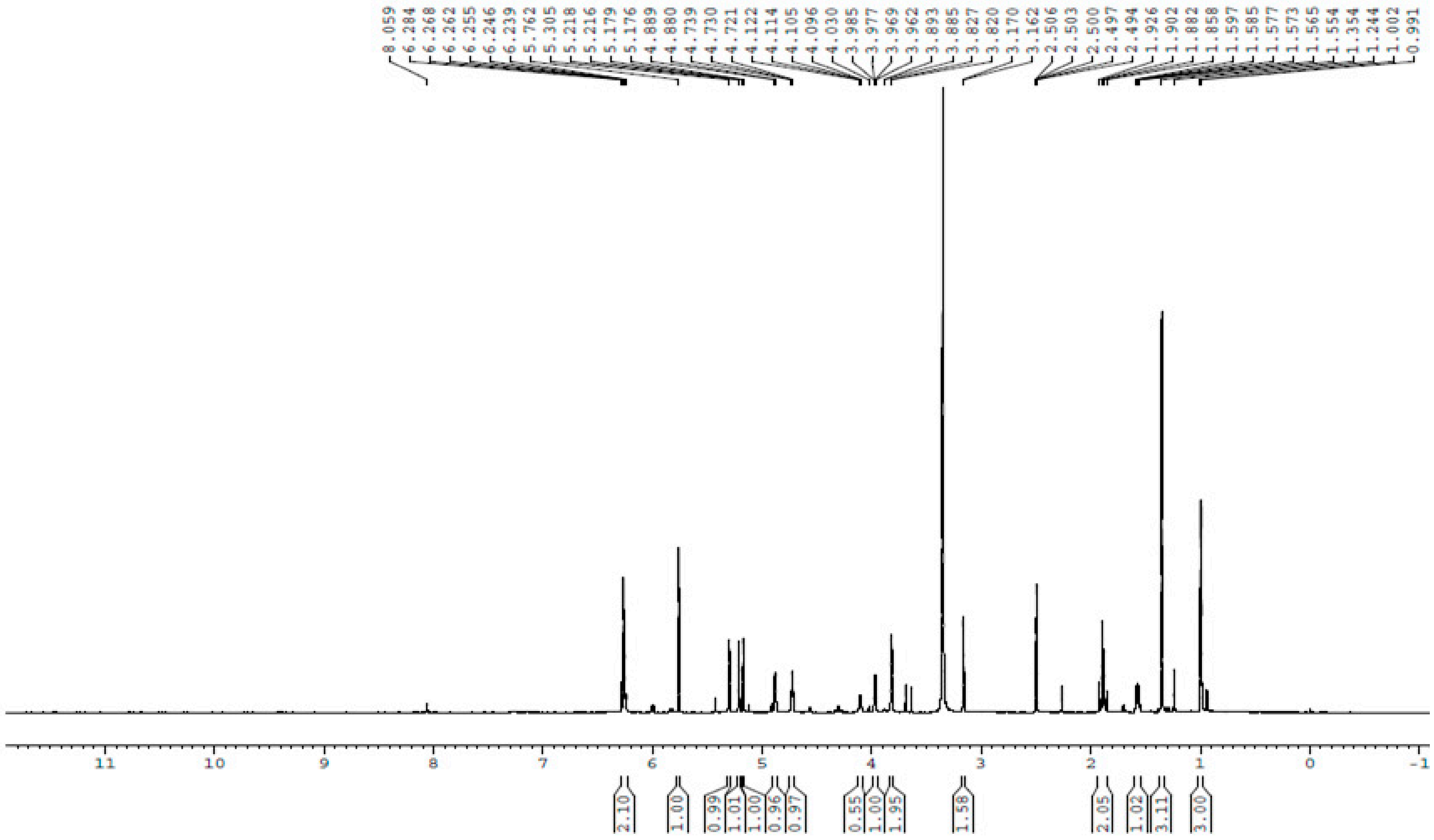

Figure S3. *Cont.*

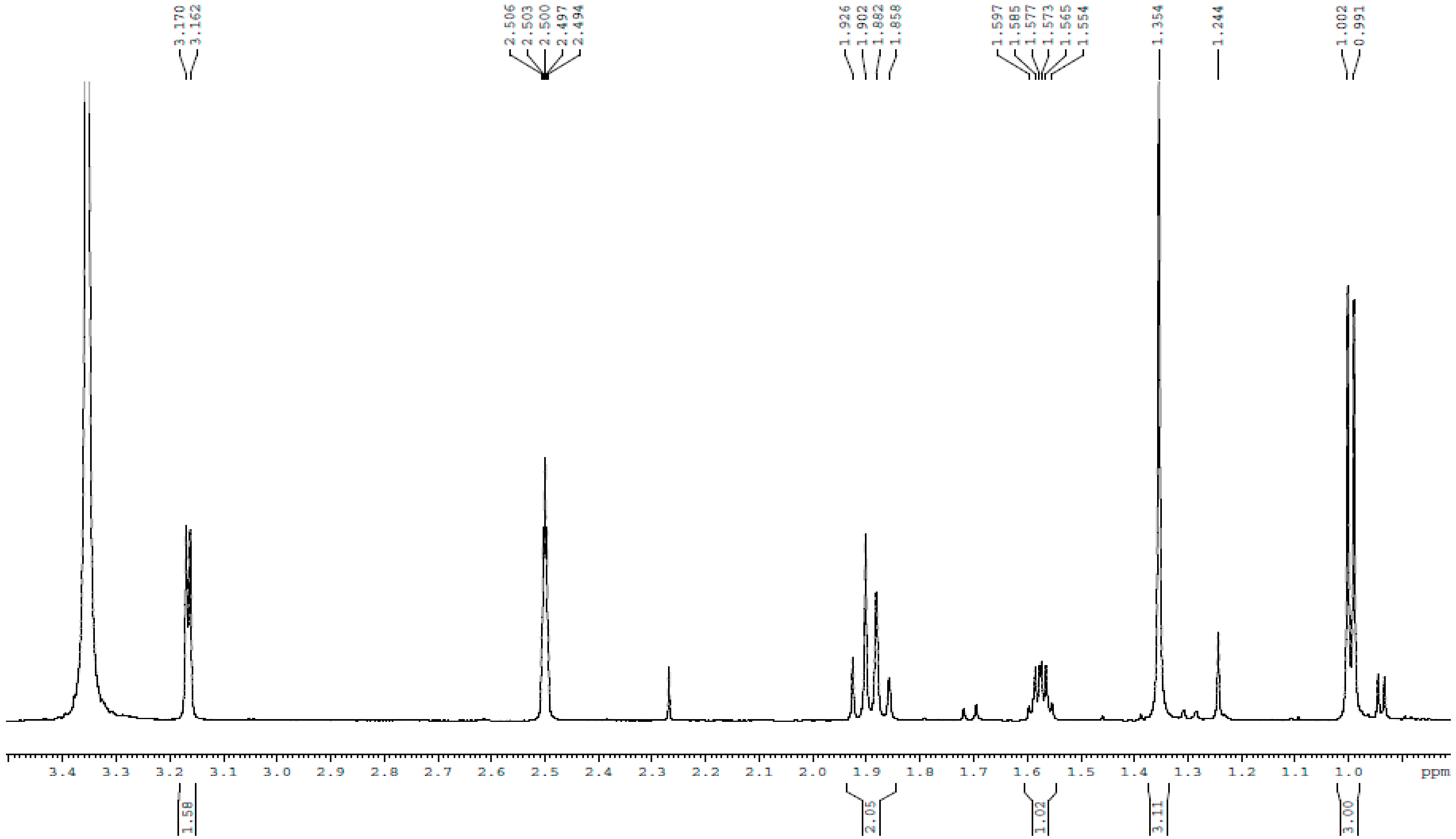

Figure S3. *Cont.*

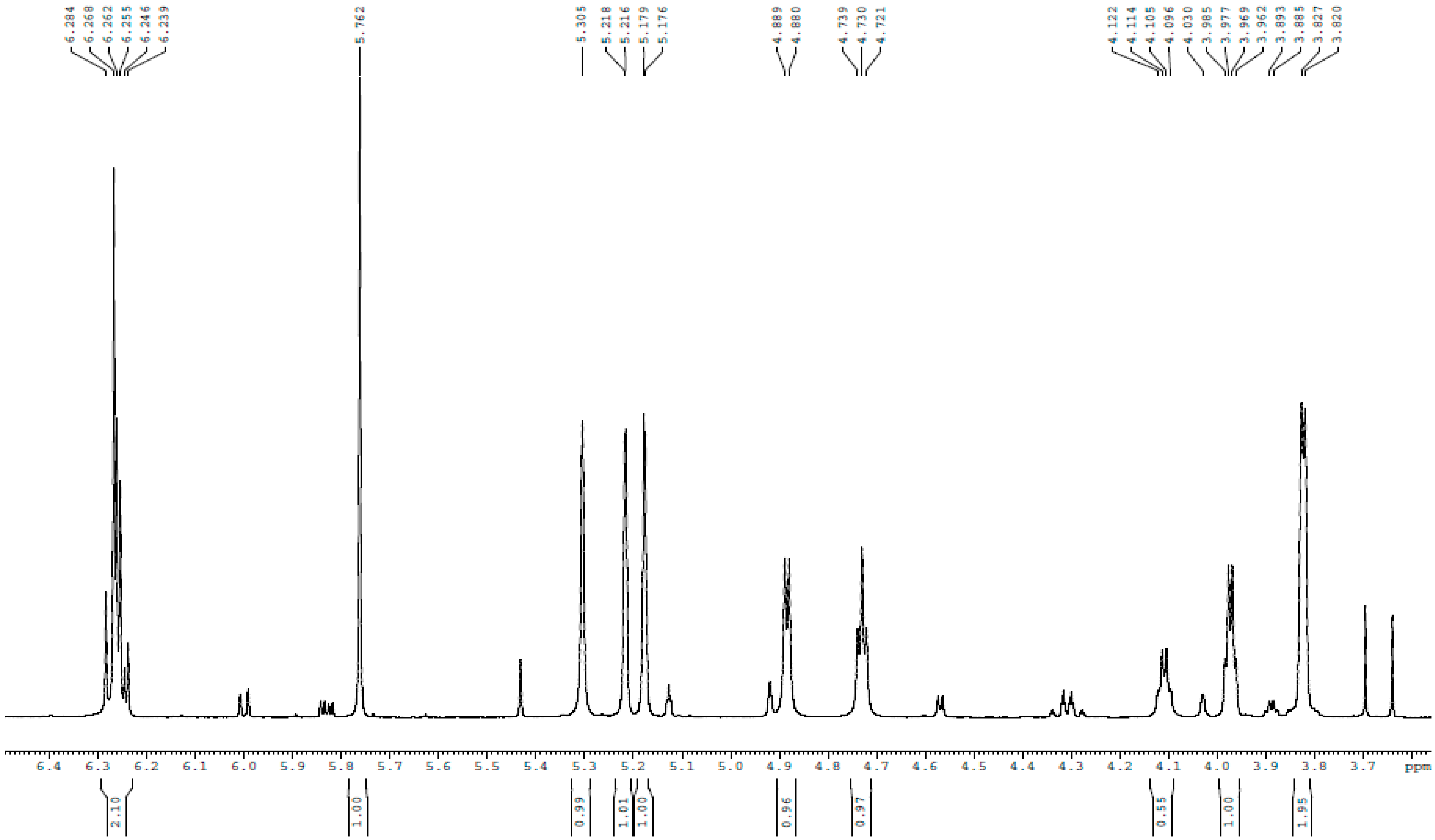

Figure S3. ^{1}H-NMR spectrum of **1** in DMSO-d$_6$.

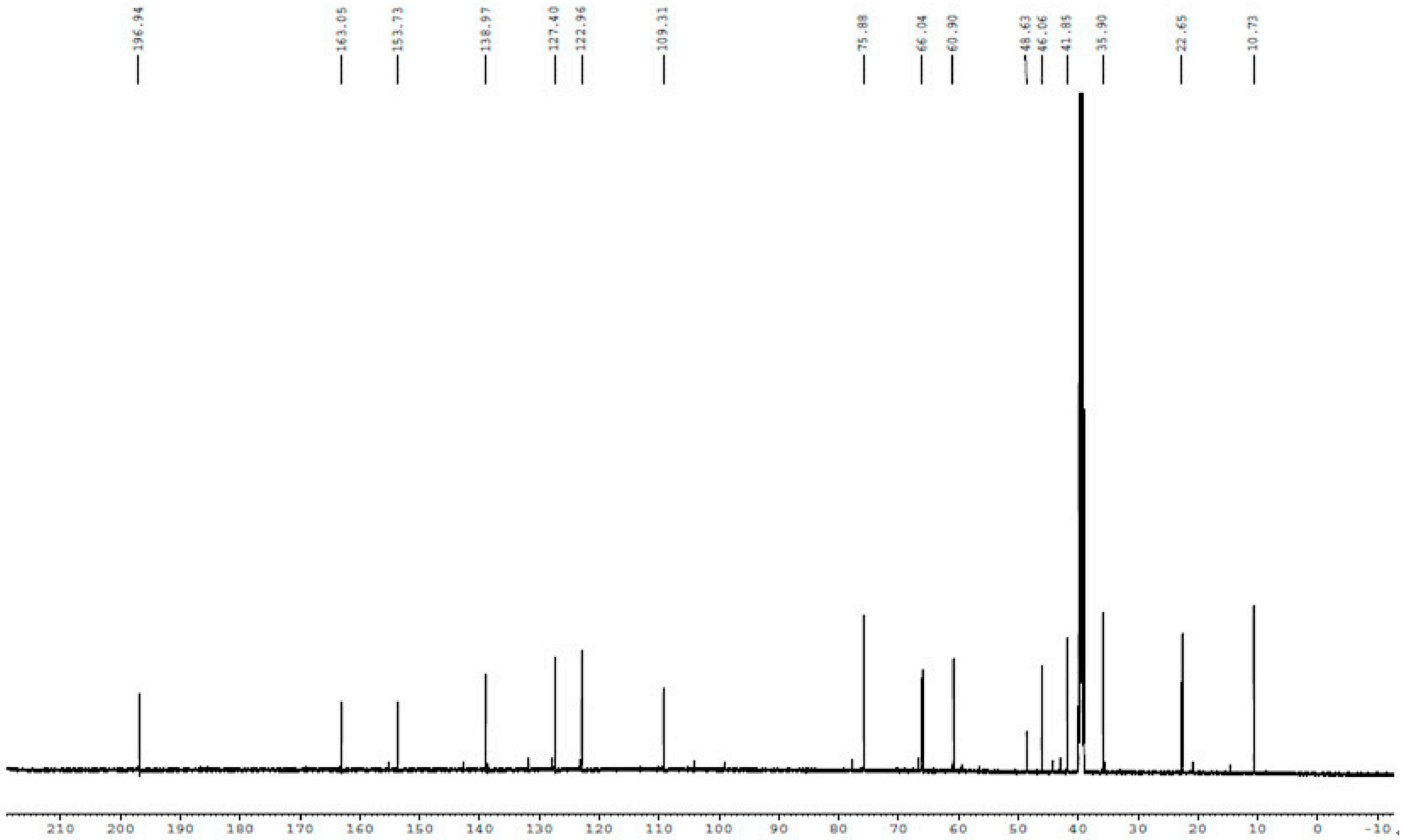

Figure S4. *Cont.*

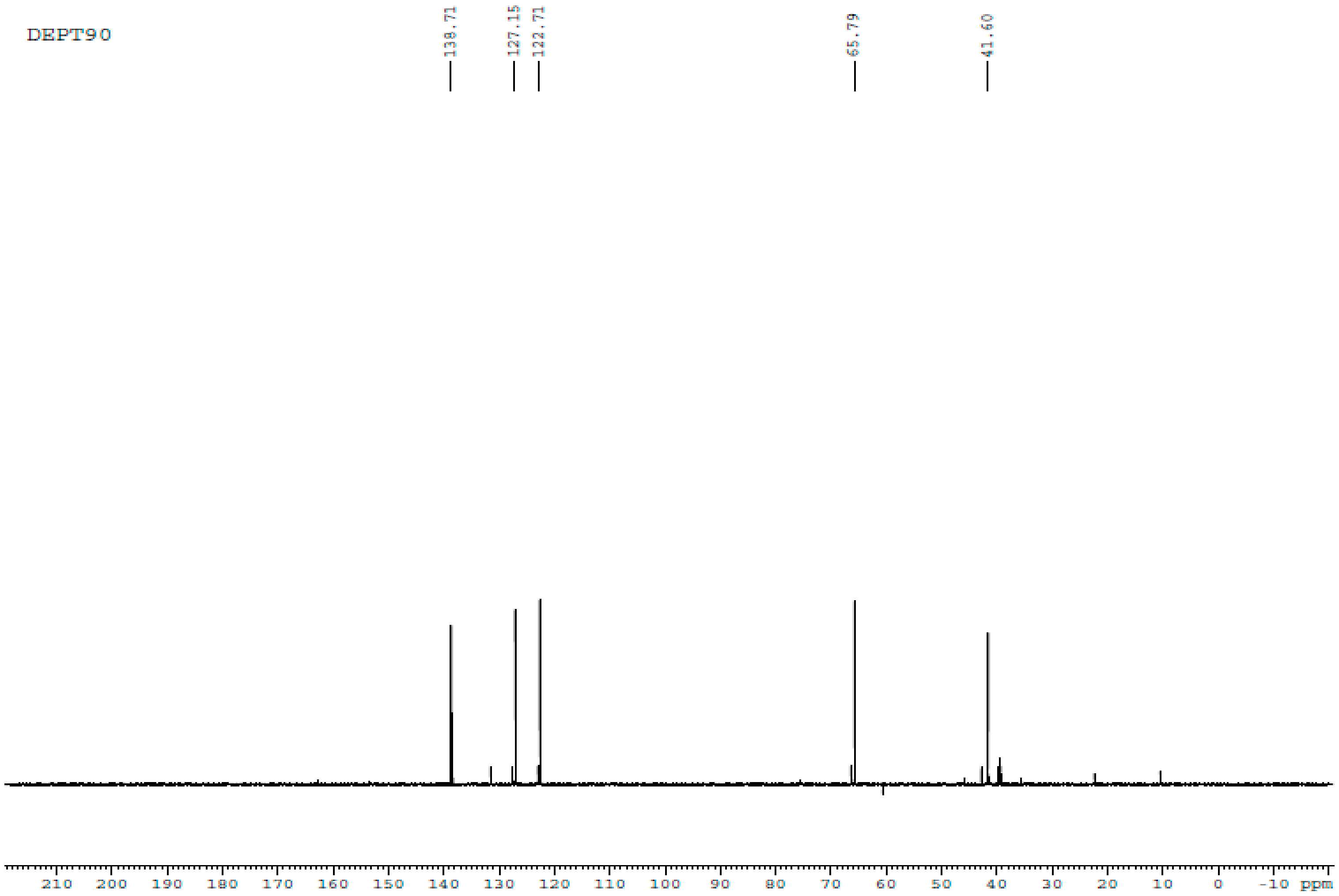

Figure S4. *Cont.*

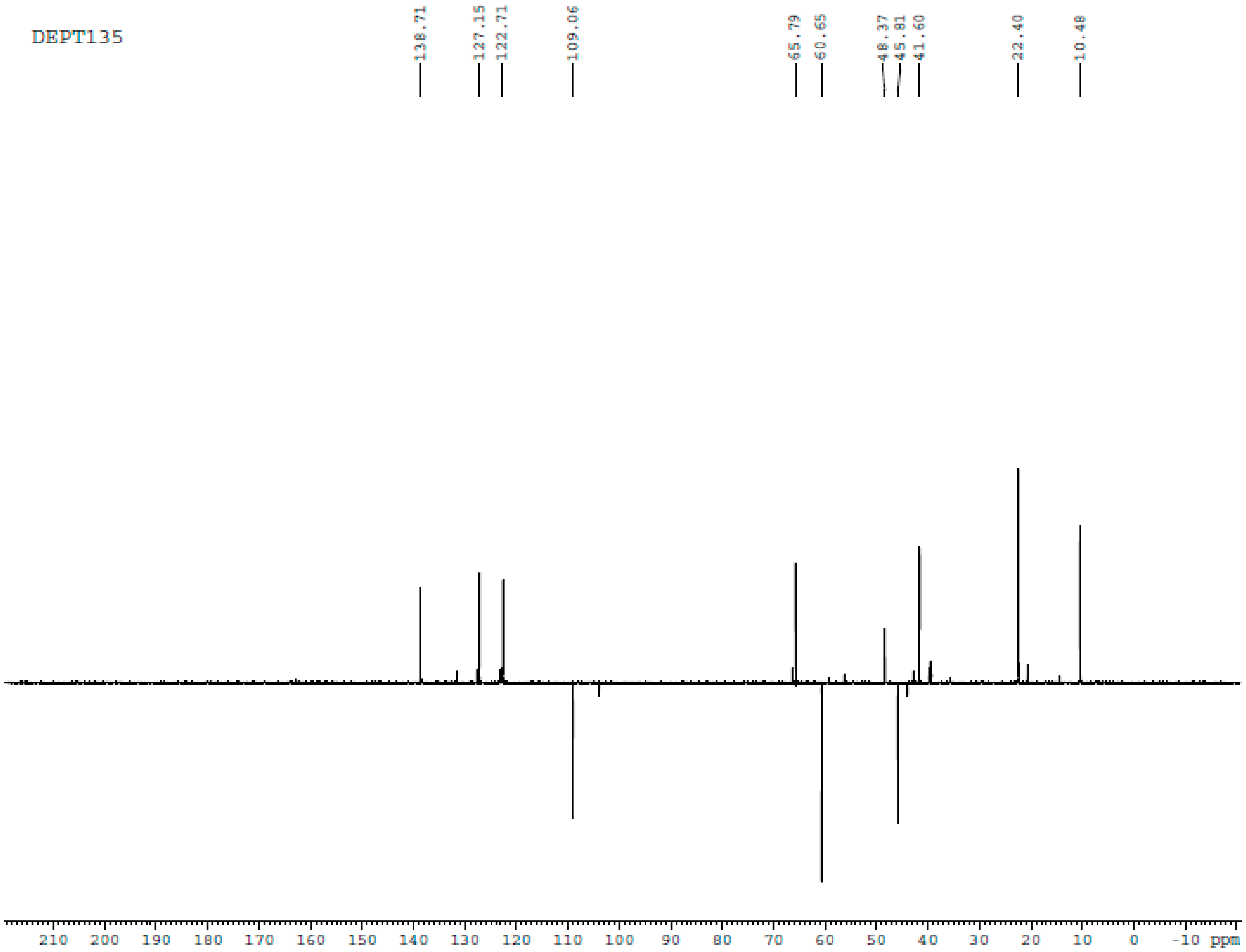

Figure S4. ^{13}C-NMR spectrum and DEPT of **1** in DMSO-*d*$_6$.

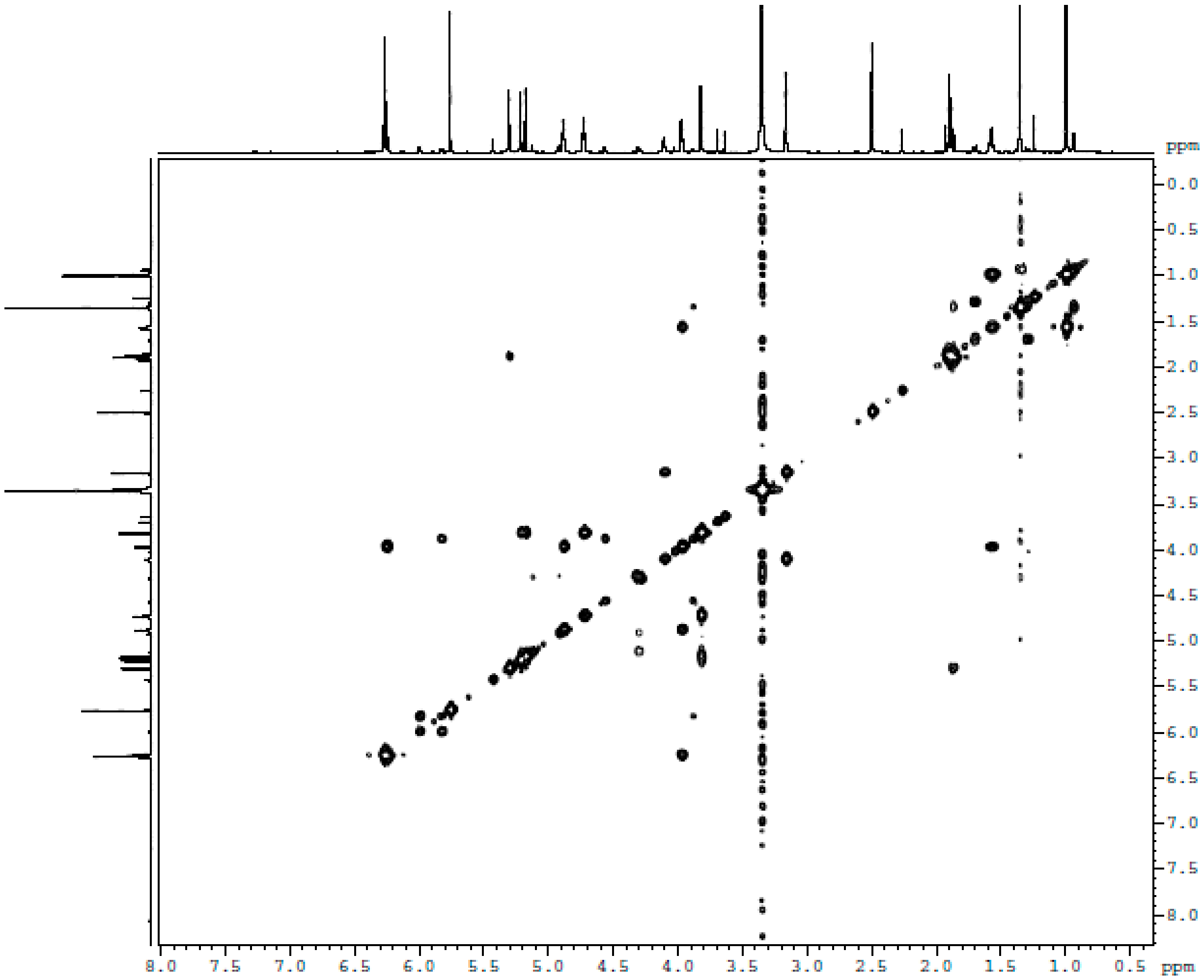

Figure S5. ^{1}H-^{1}H COSY spectrum of **1** in DMSO-d_6.

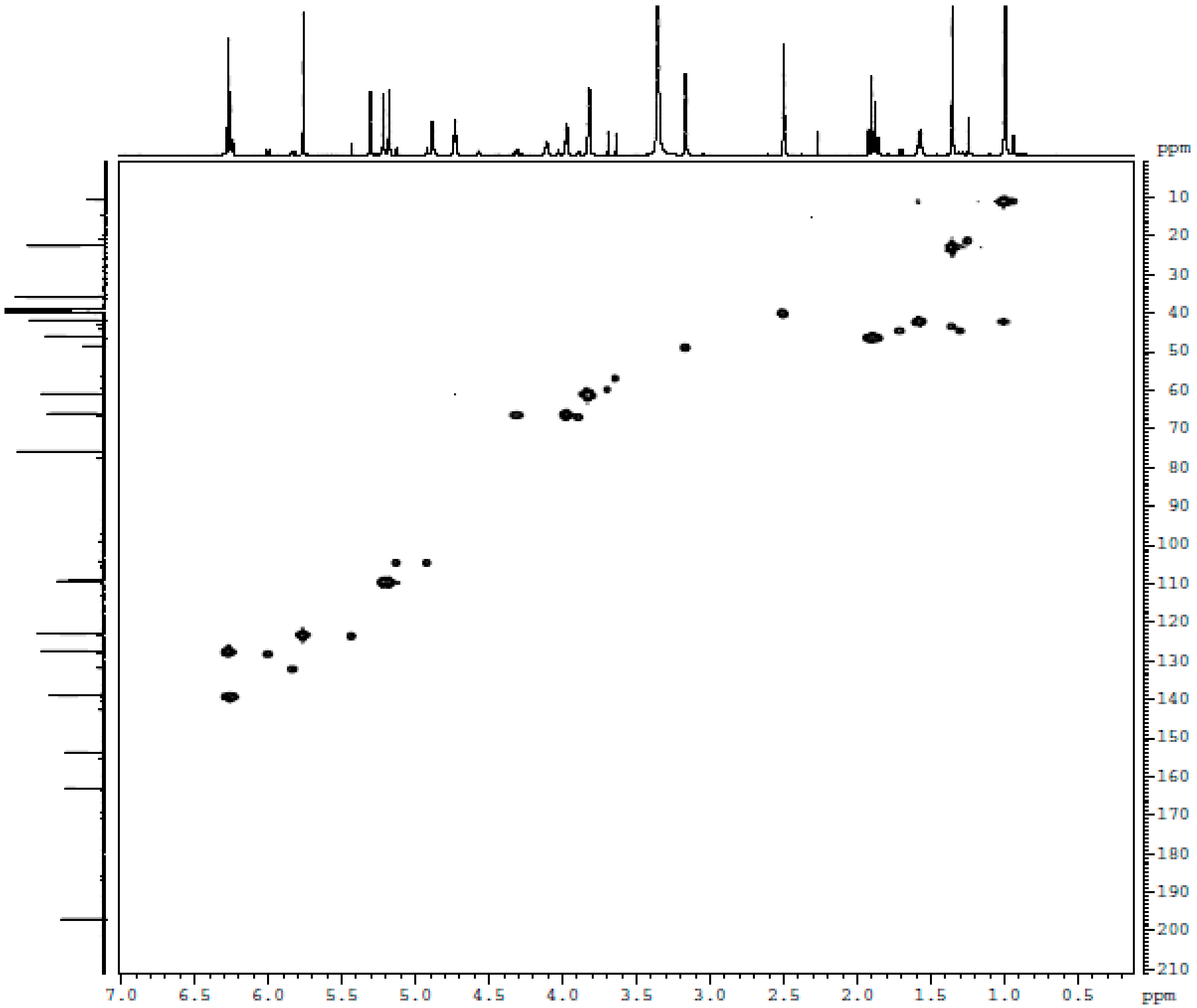

Figure S6. HSQC spectrum of **1** in DMSO-d_6.

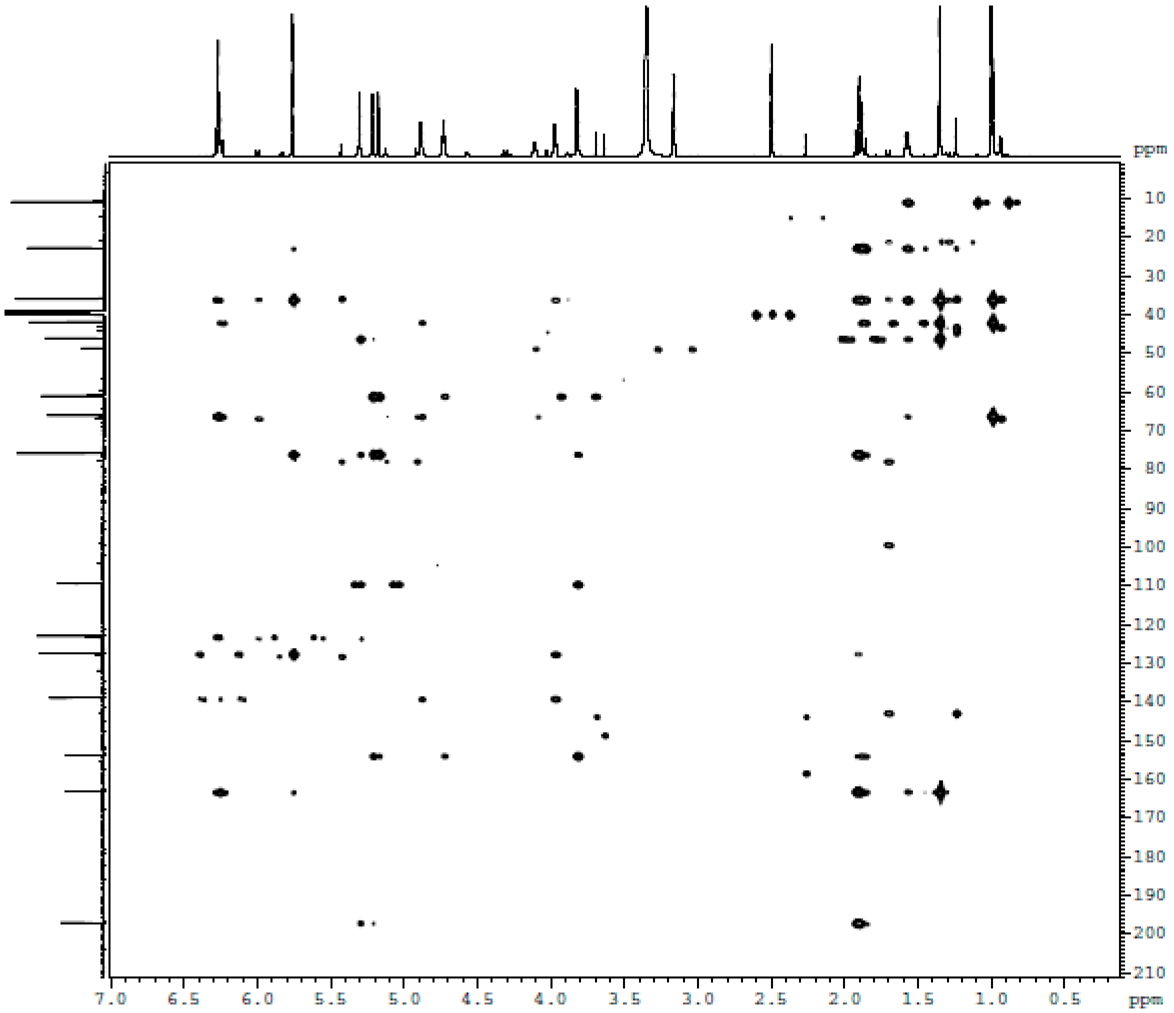

Figure S7. HMBC spectrum of **1** in DMSO-d$_6$.

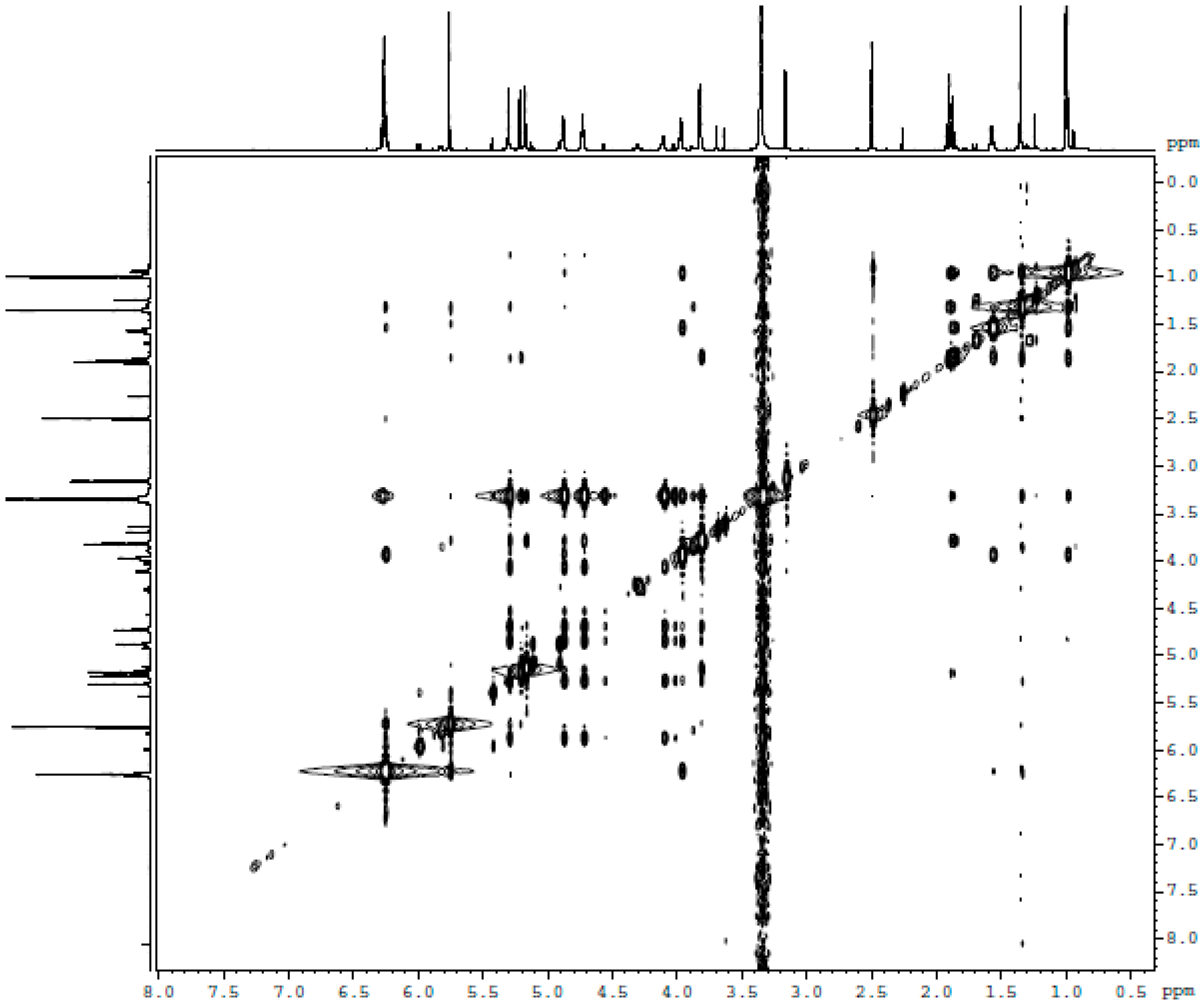

Figure S8. NOESY spectrum of **1** in DMSO-d_6.

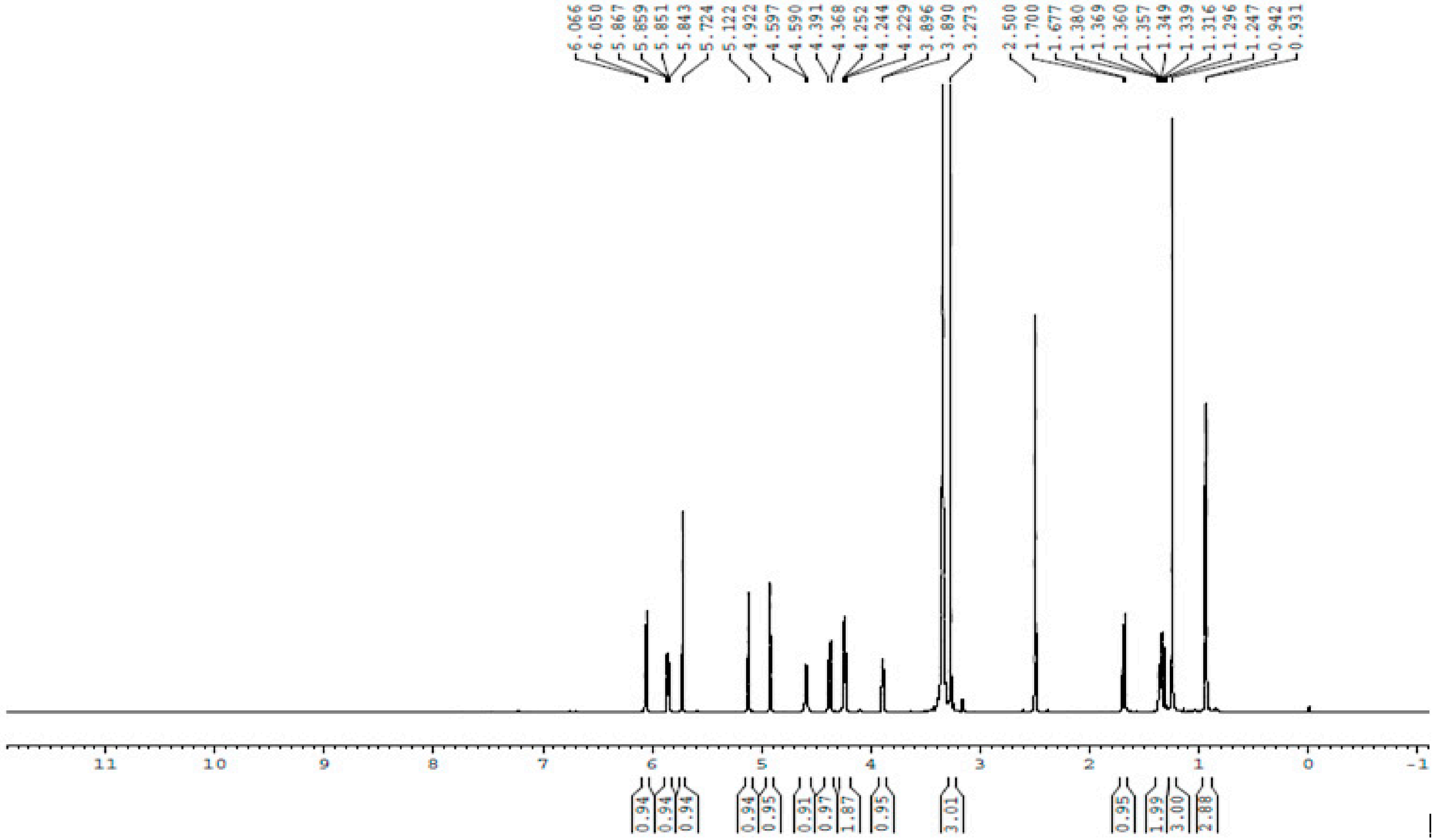

Figure S9. *Cont.*

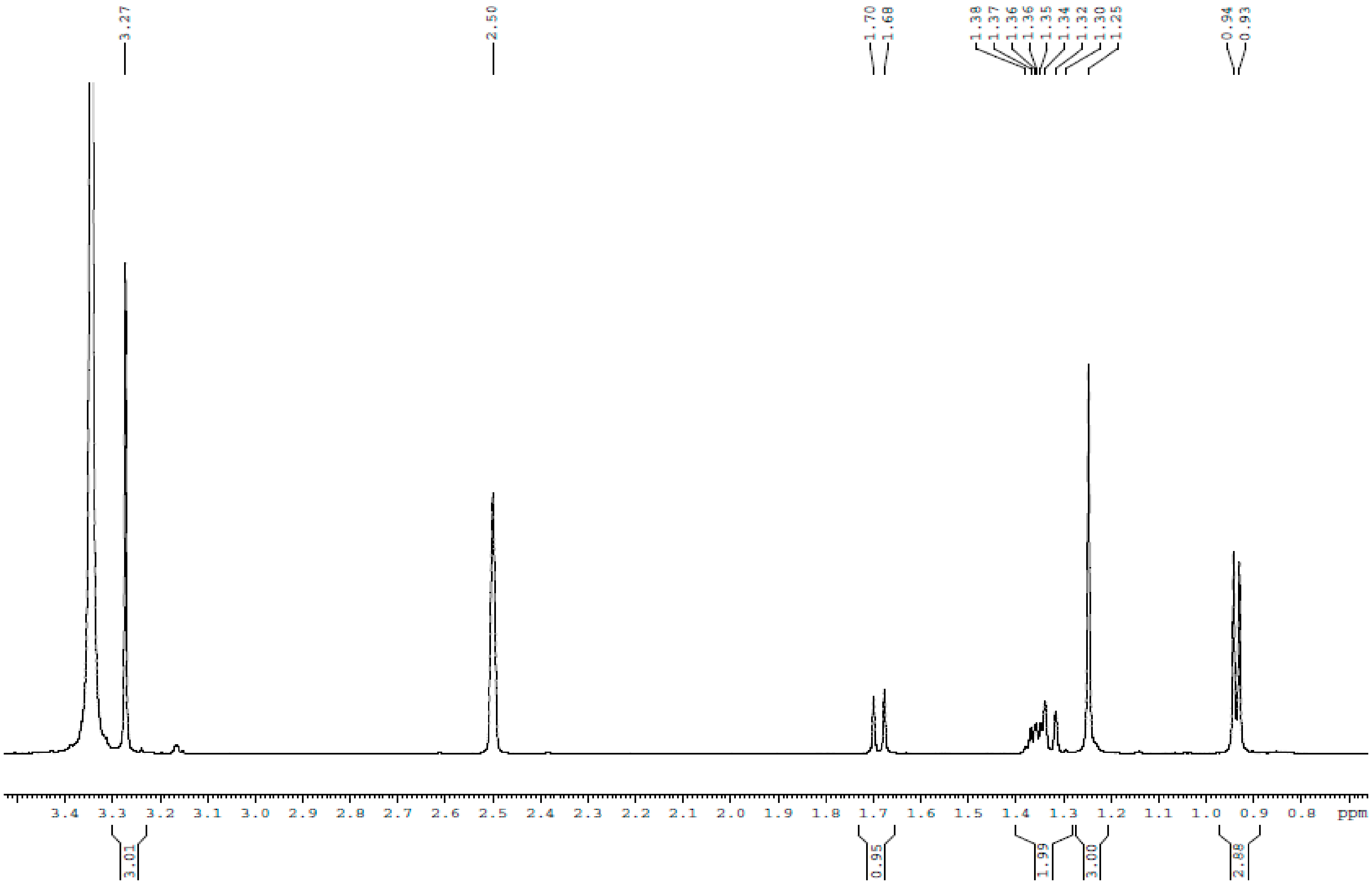

Figure S9. *Cont.*

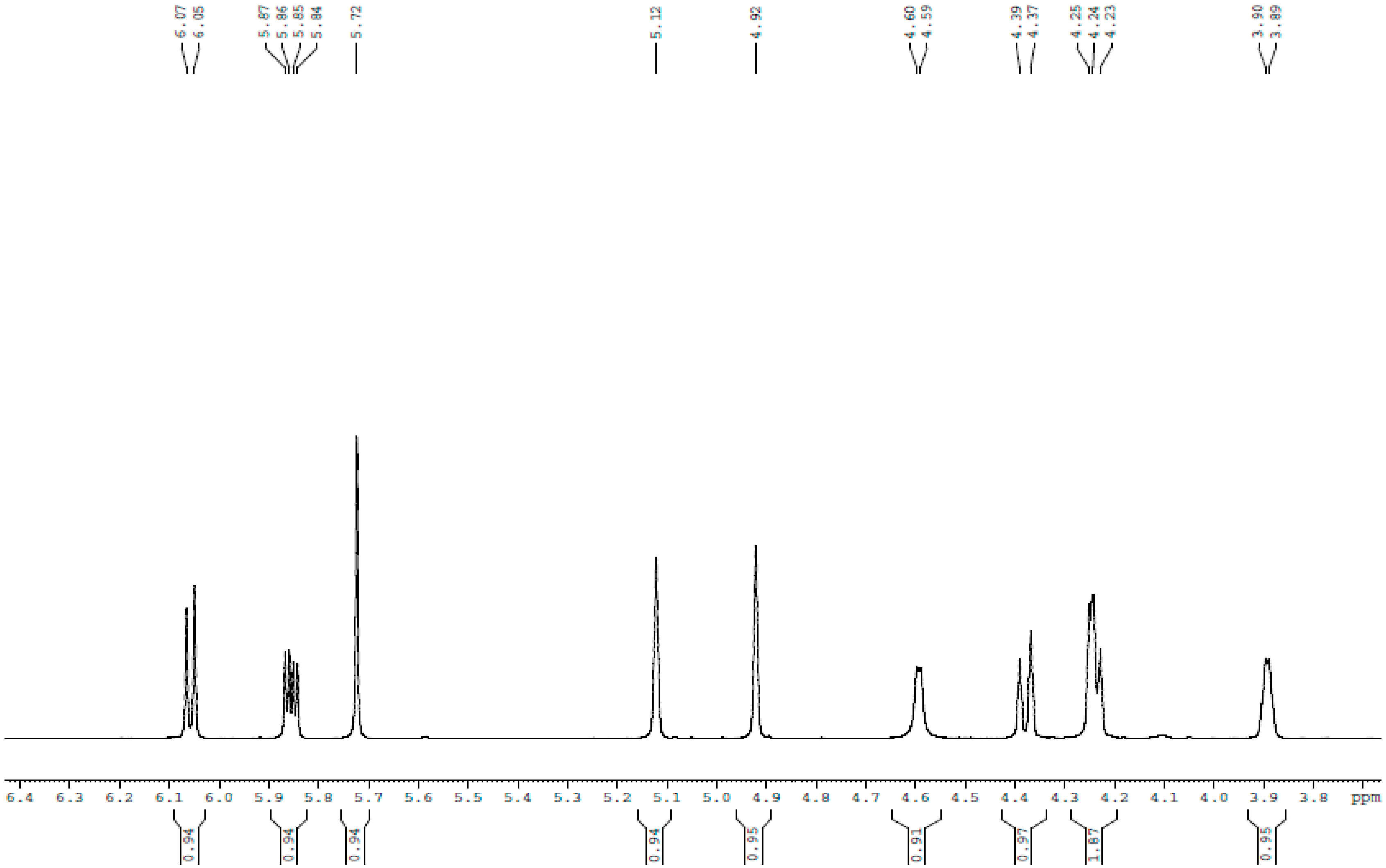

Figure S9. ^{1}H-NMR spectrum of **2** in DMSO-d_6.

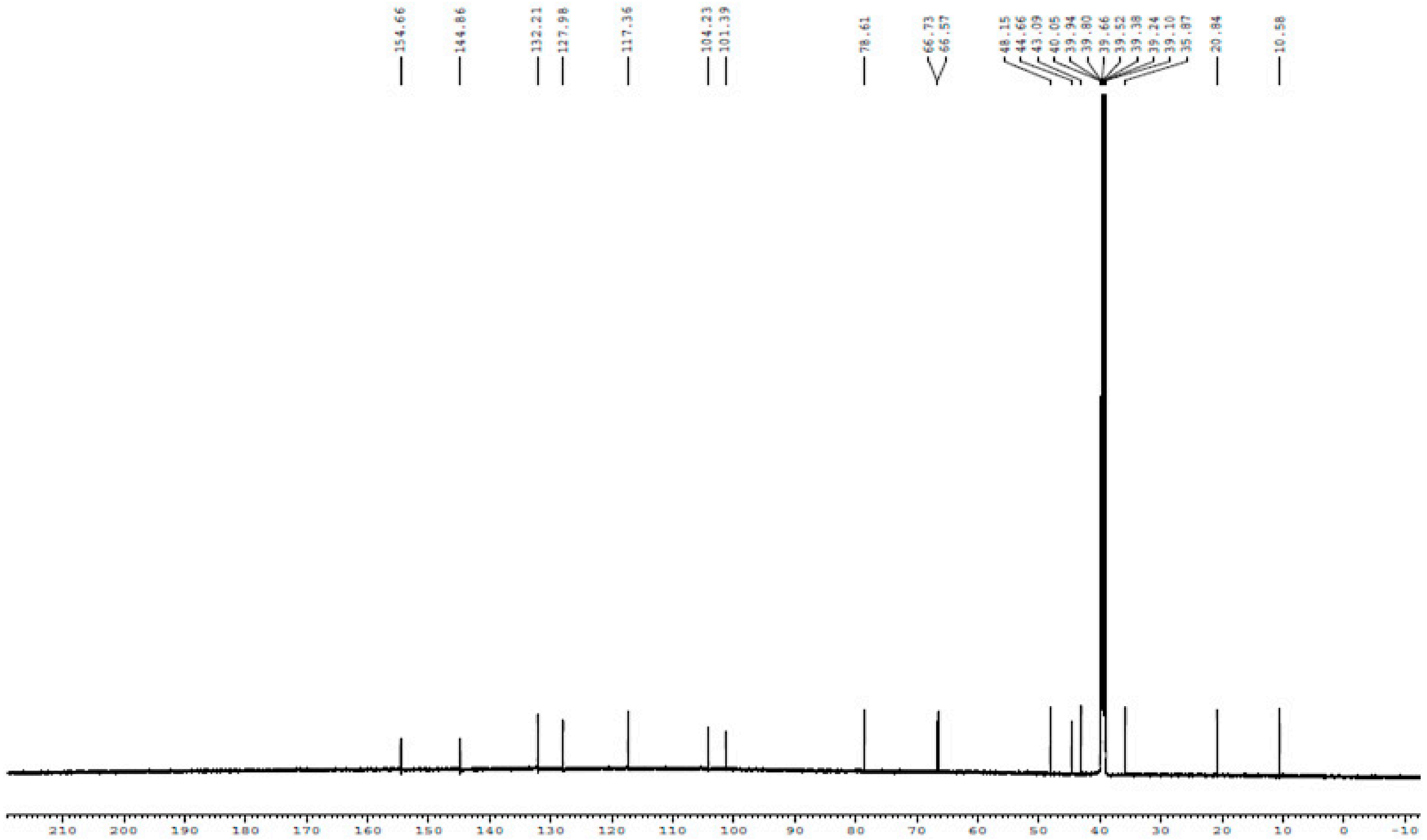

Figure S10. *Cont.*

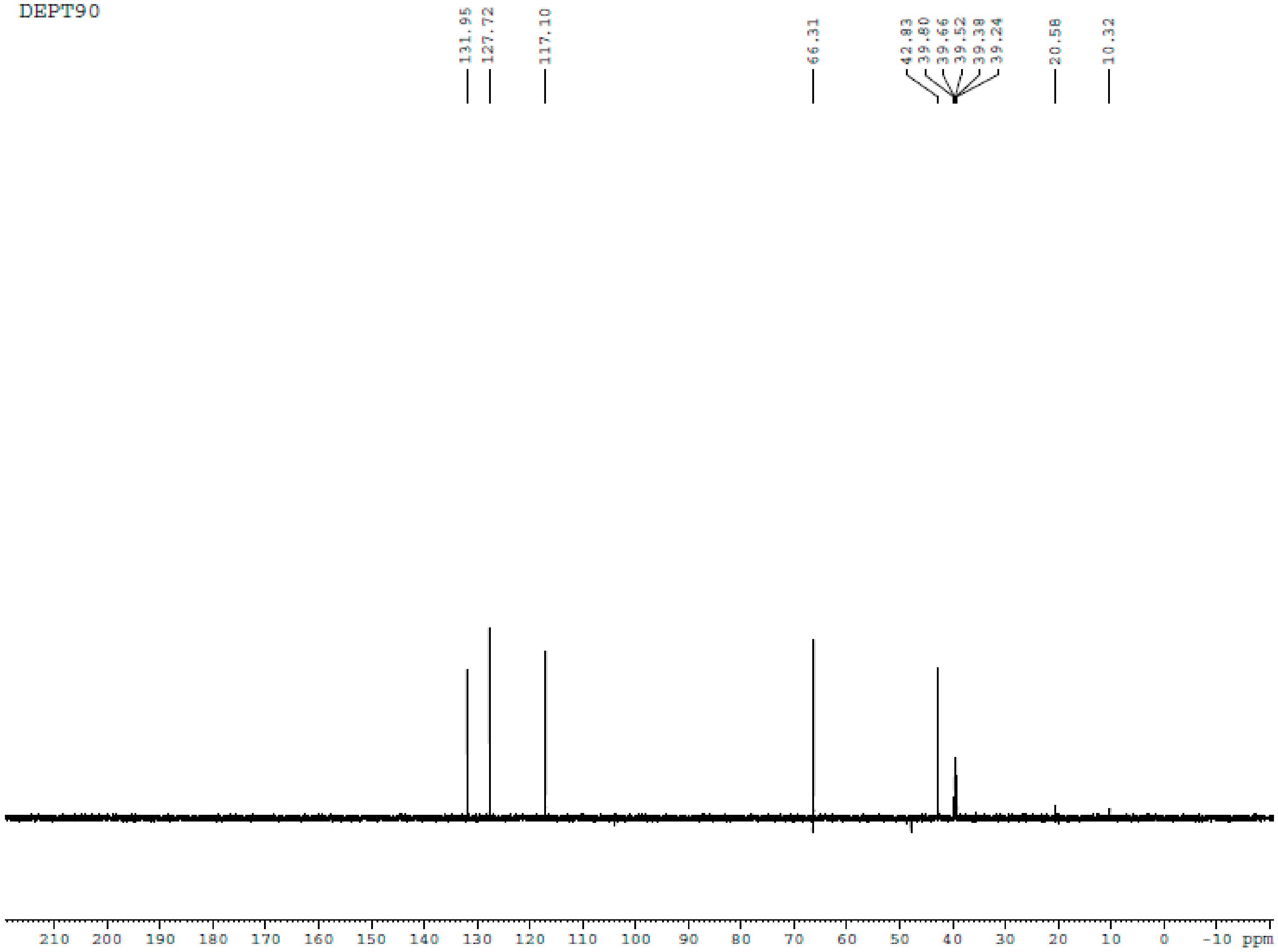

Figure S10. *Cont.*

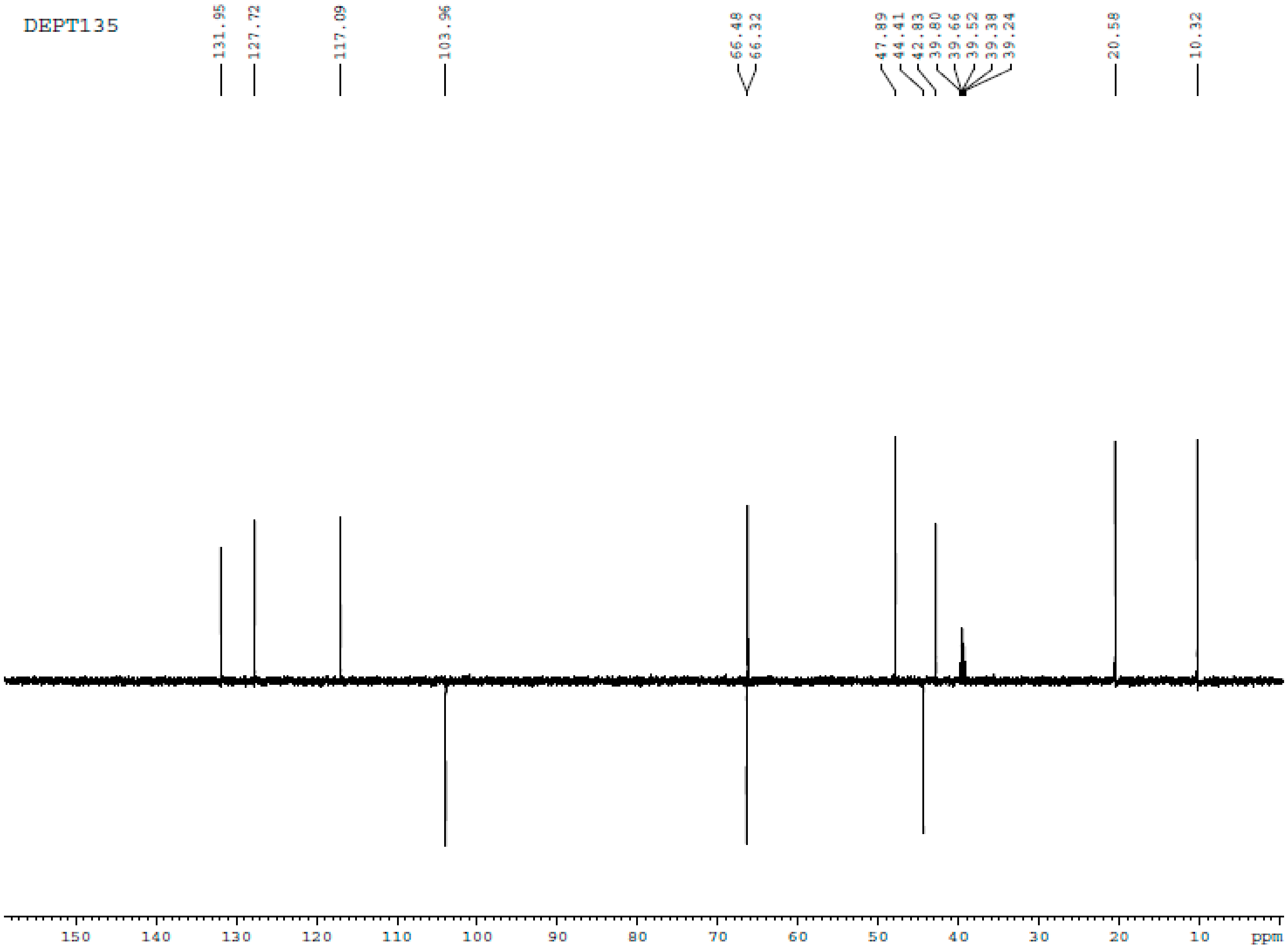

Figure S10. ^{13}C-NMR spectrum and DEPT of **2** in DMSO-*d*6.

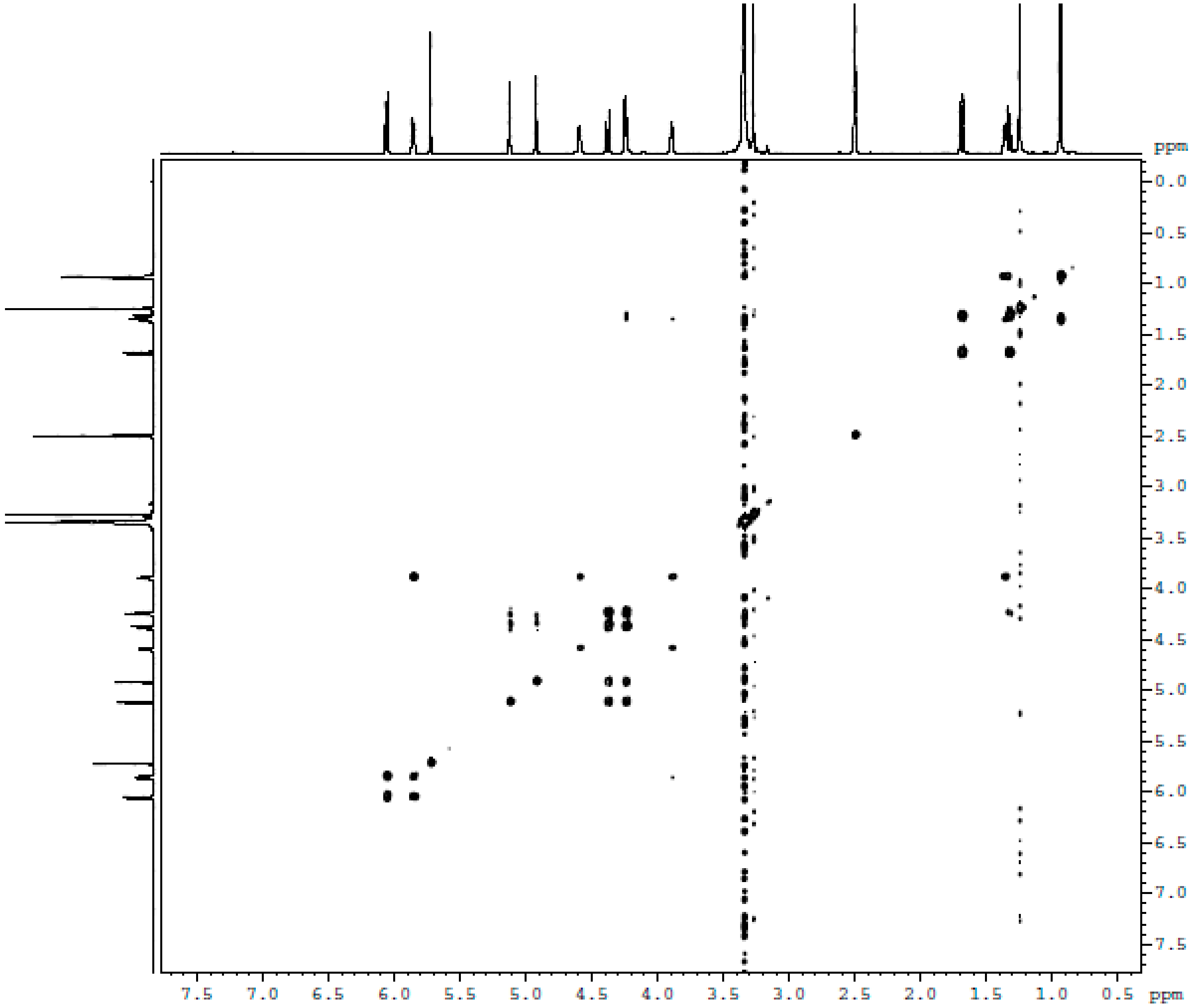

Figure S11. ^1H-^1H COSY spectrum of **2** in DMSO-d_6.

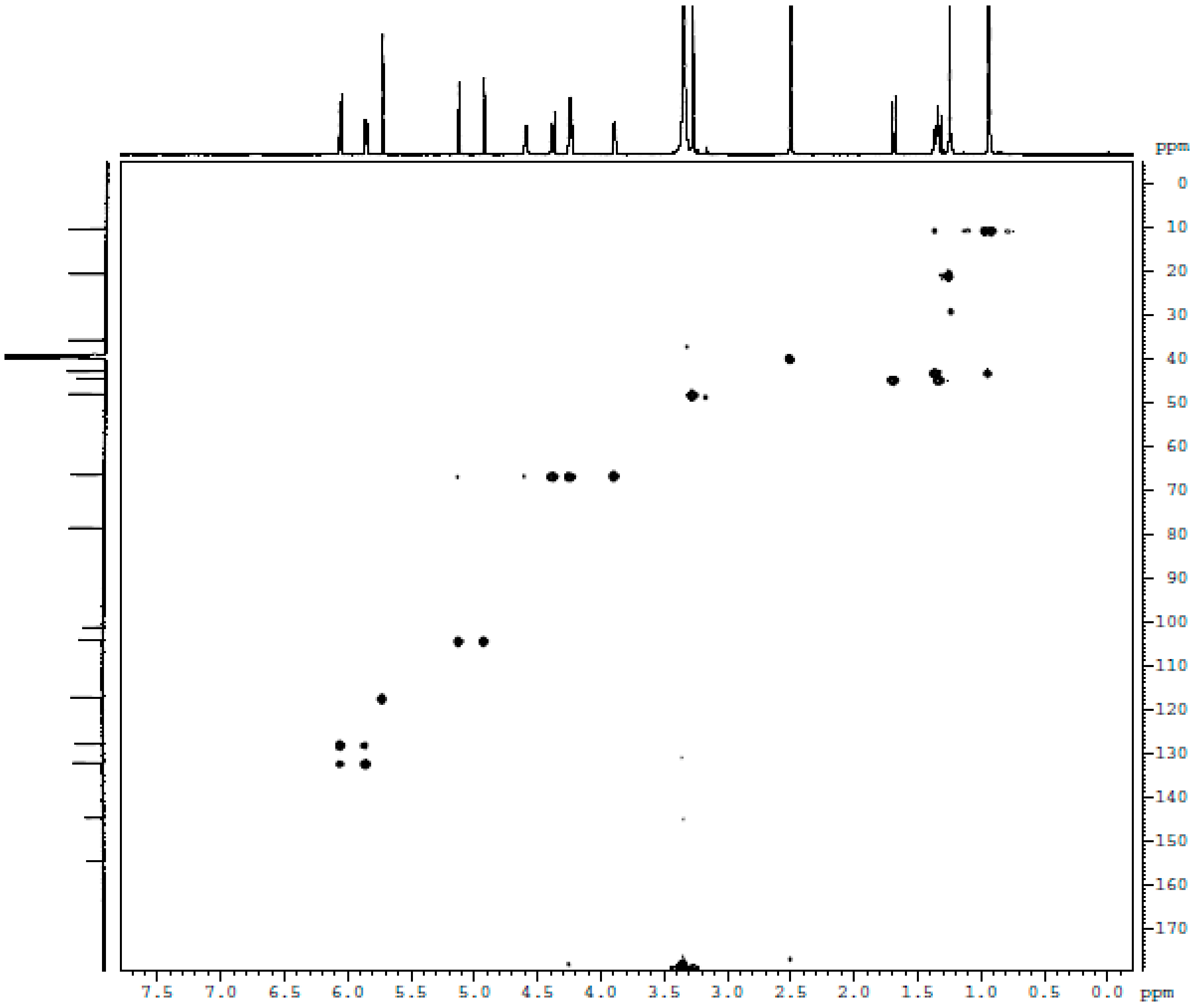

Figure S12. HSQC spectrum of **2** in DMSO-d_6.

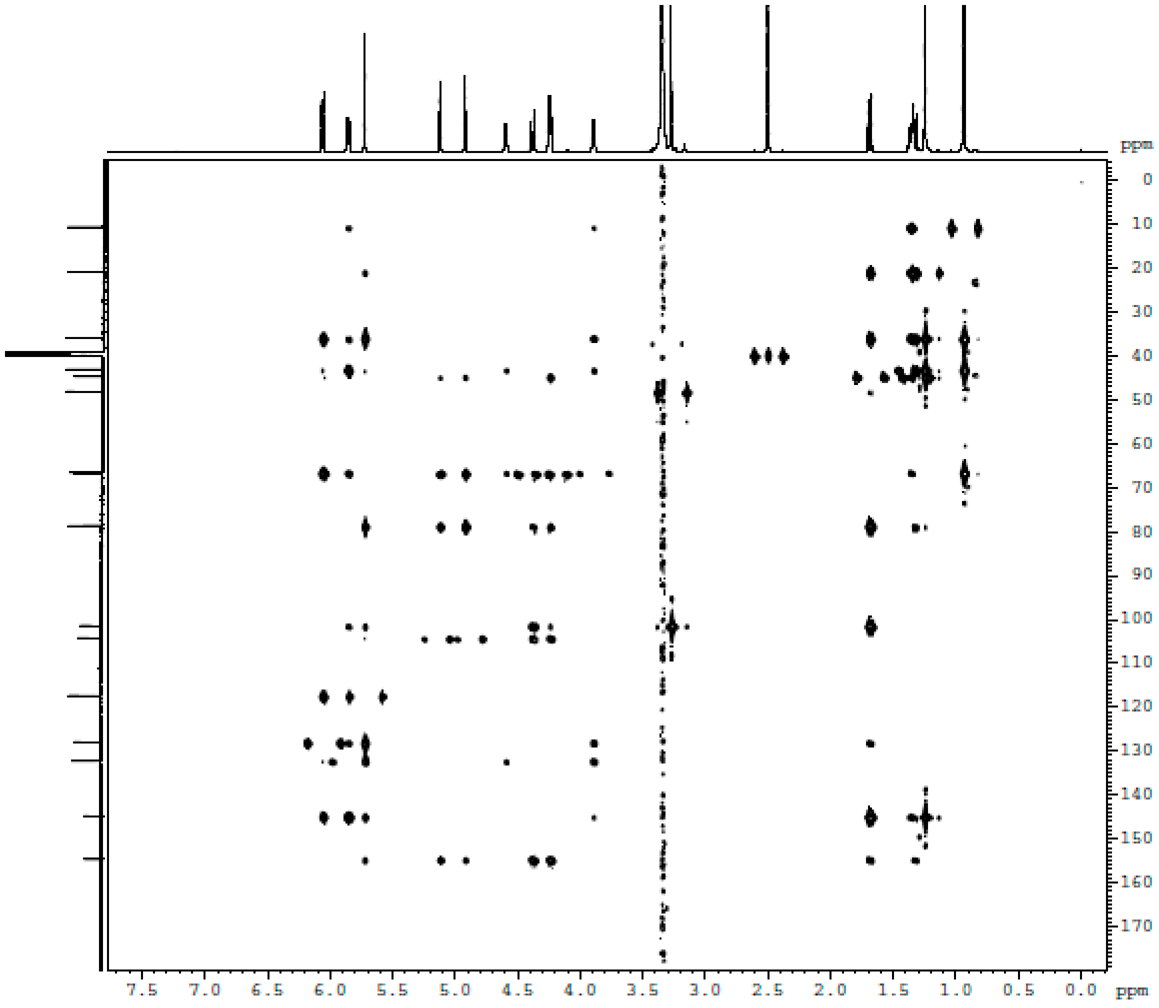

Figure S13. HMBC spectrum of **2** in DMSO-*d*6.

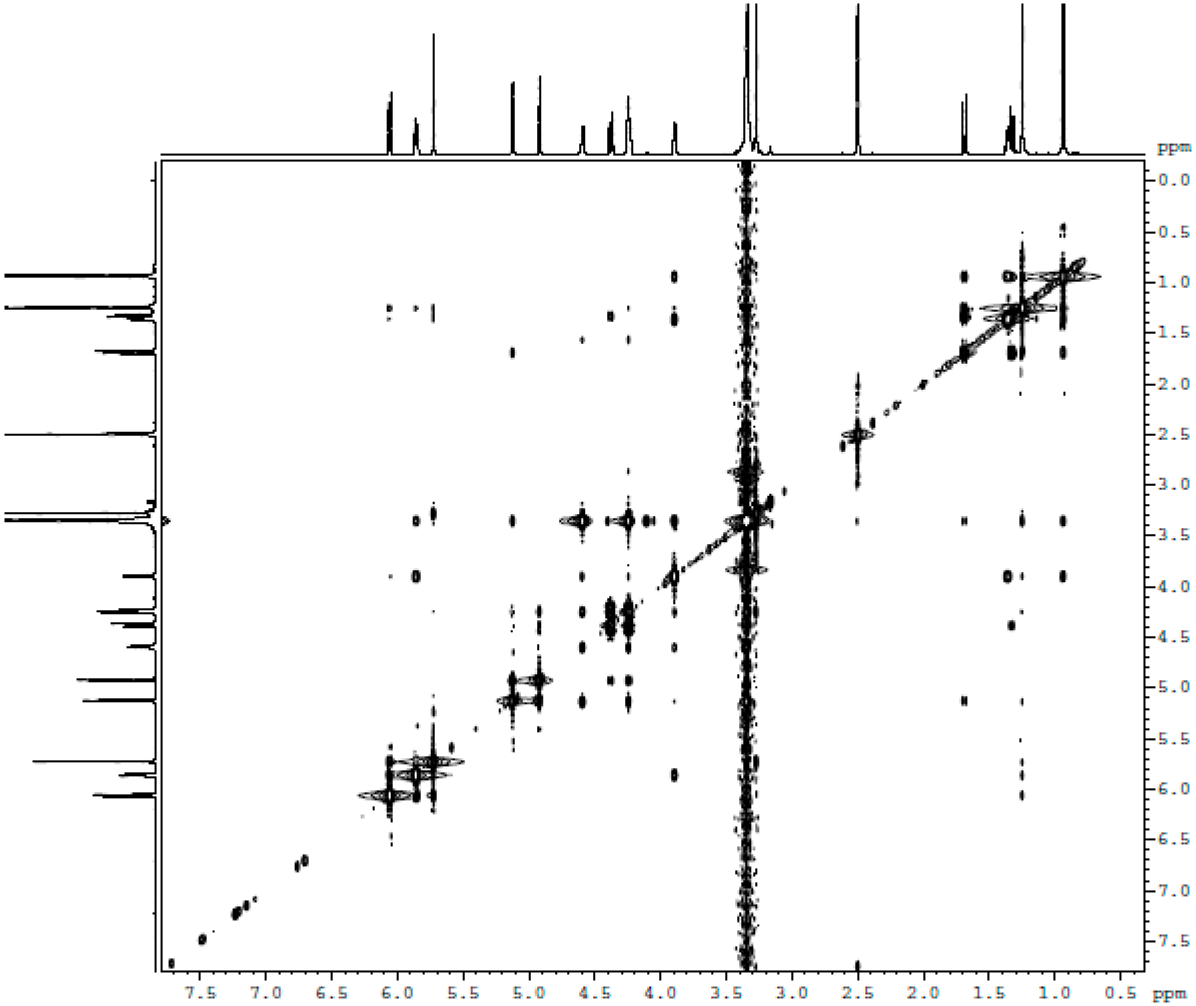

Figure S14. NOESY spectrum of **2** in DMSO-d_6.

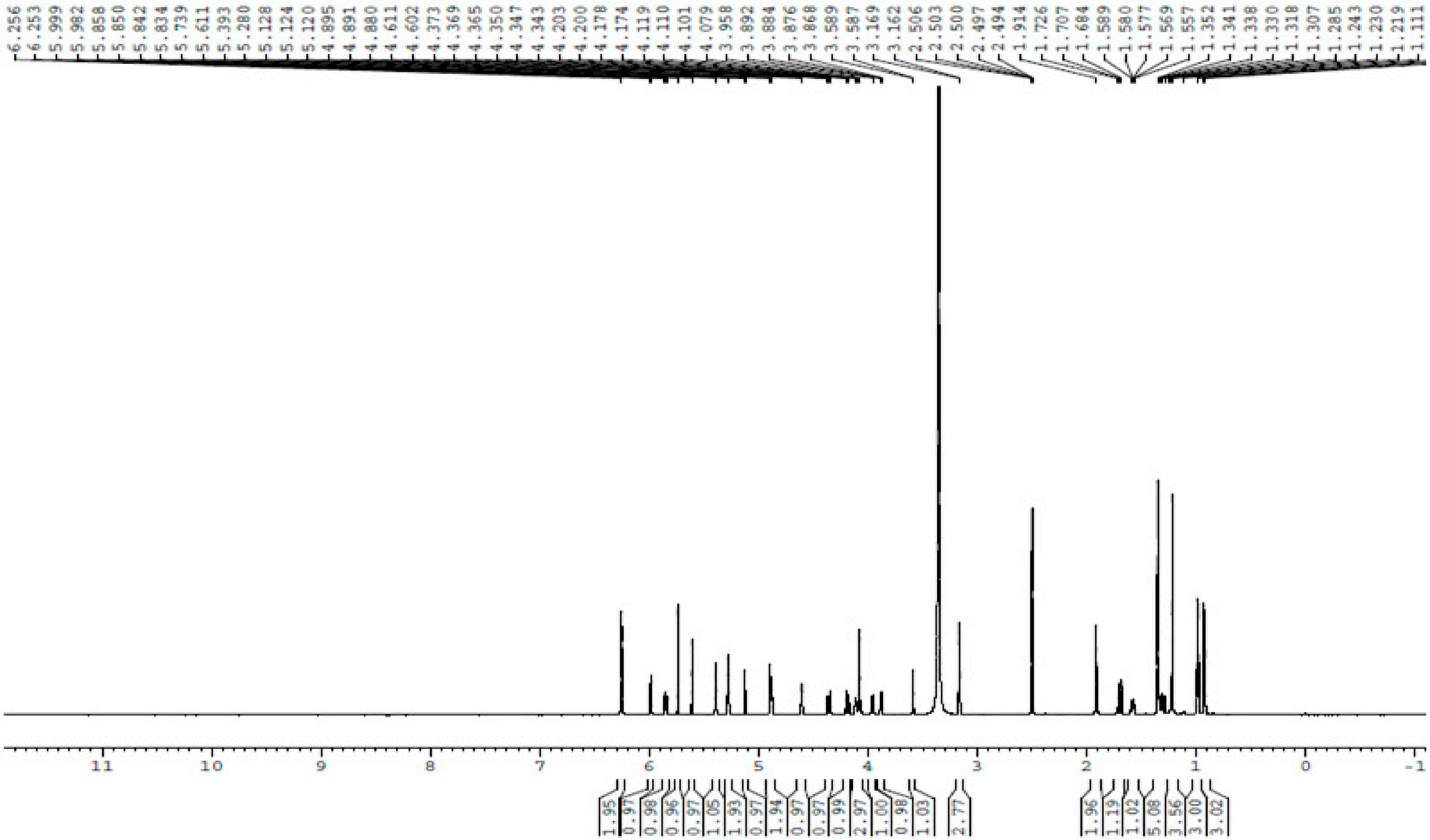

Figure S15. *Cont.*

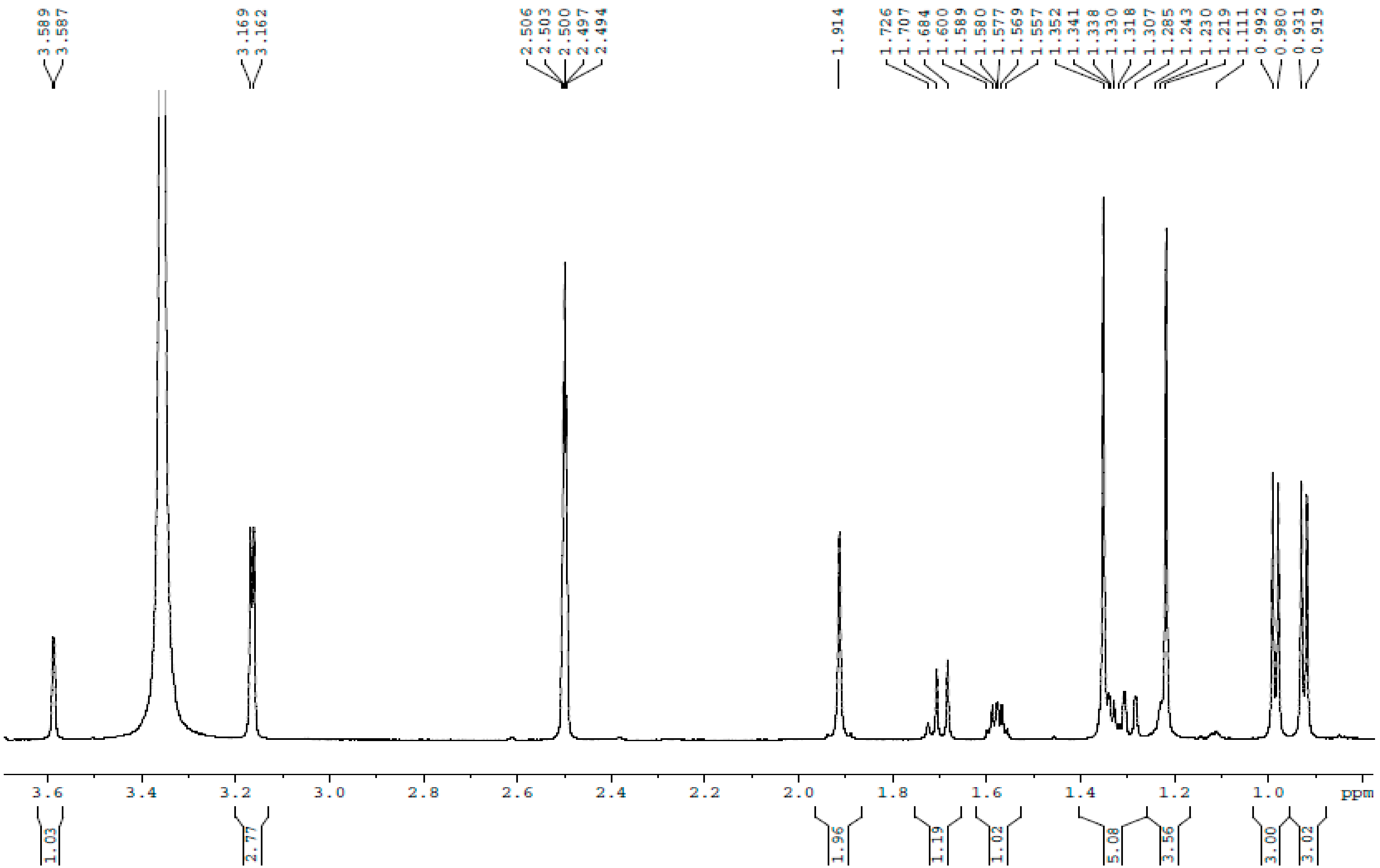

Figure S15. *Cont.*

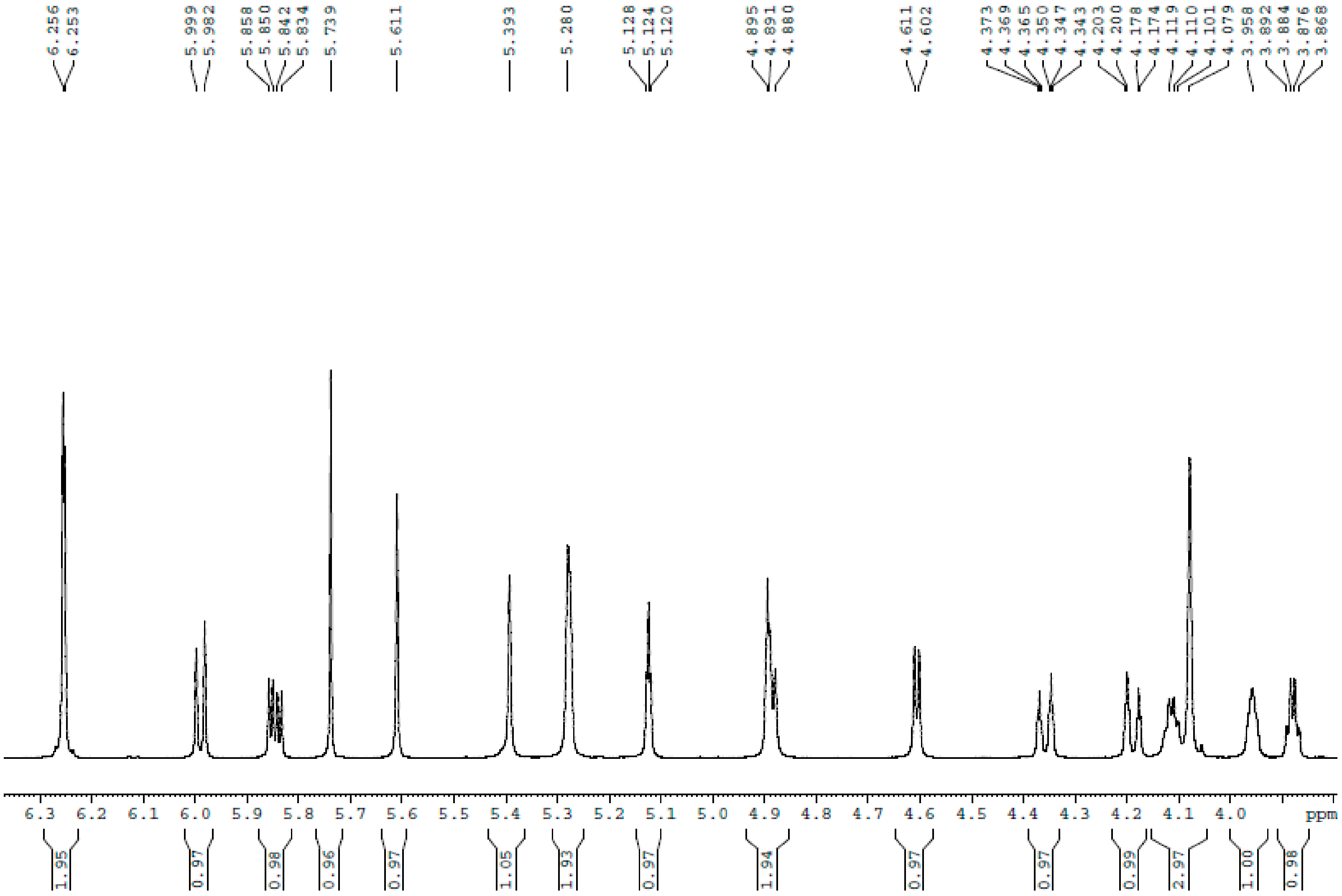

Figure S15. ^{1}H-NMR spectrum of **3** in DMSO-d_6.

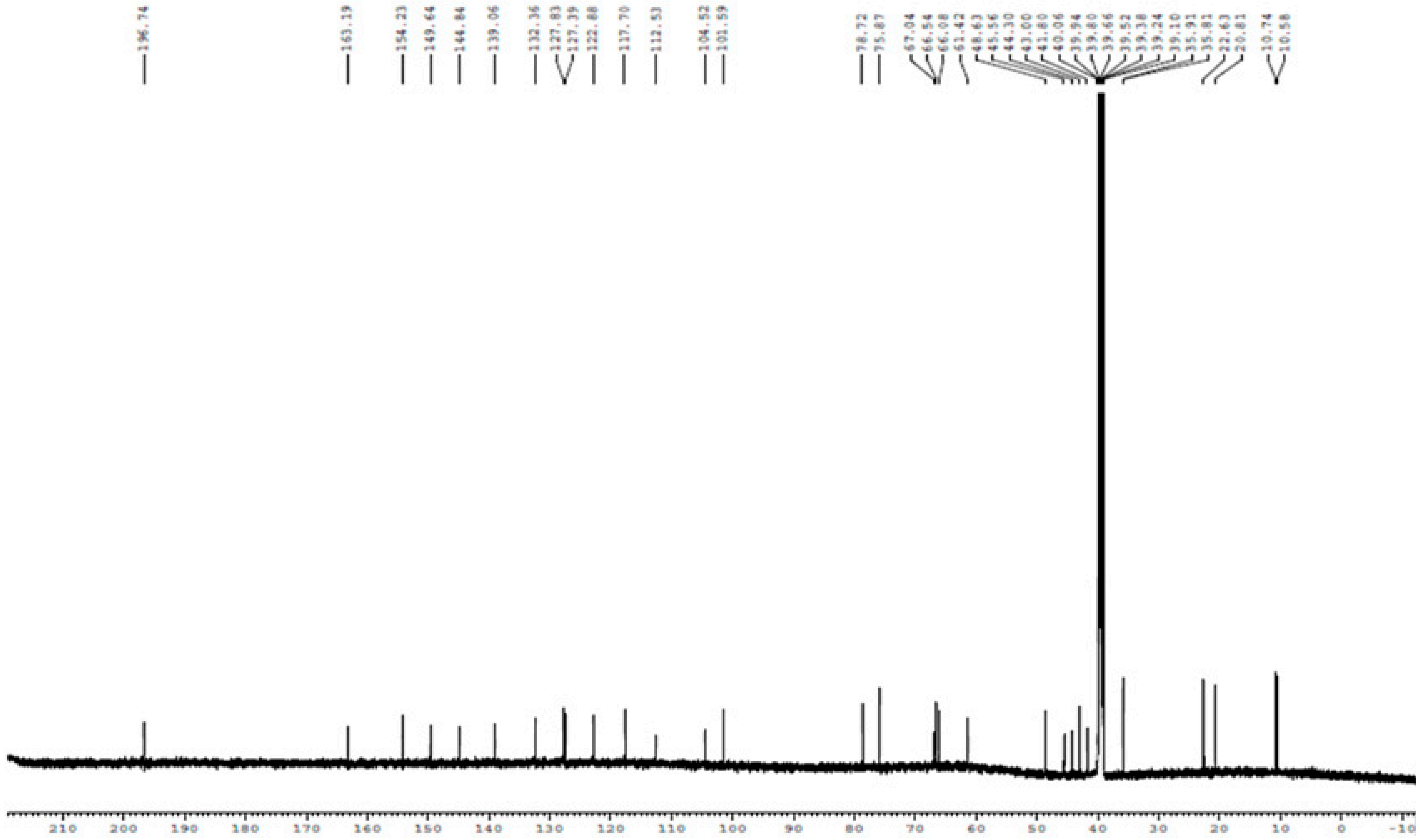

Figure S16. *Cont.*

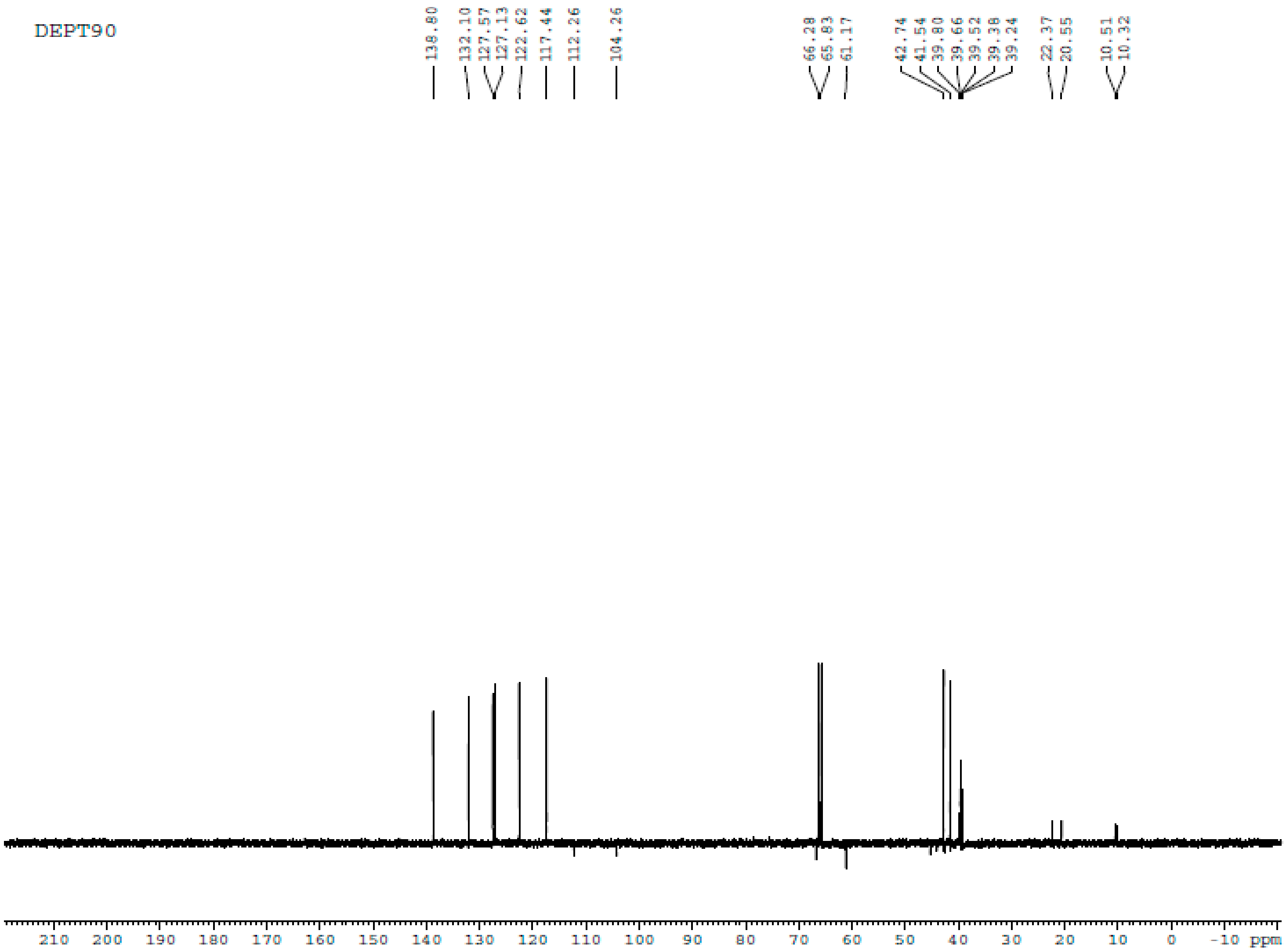

Figure S16. *Cont.*

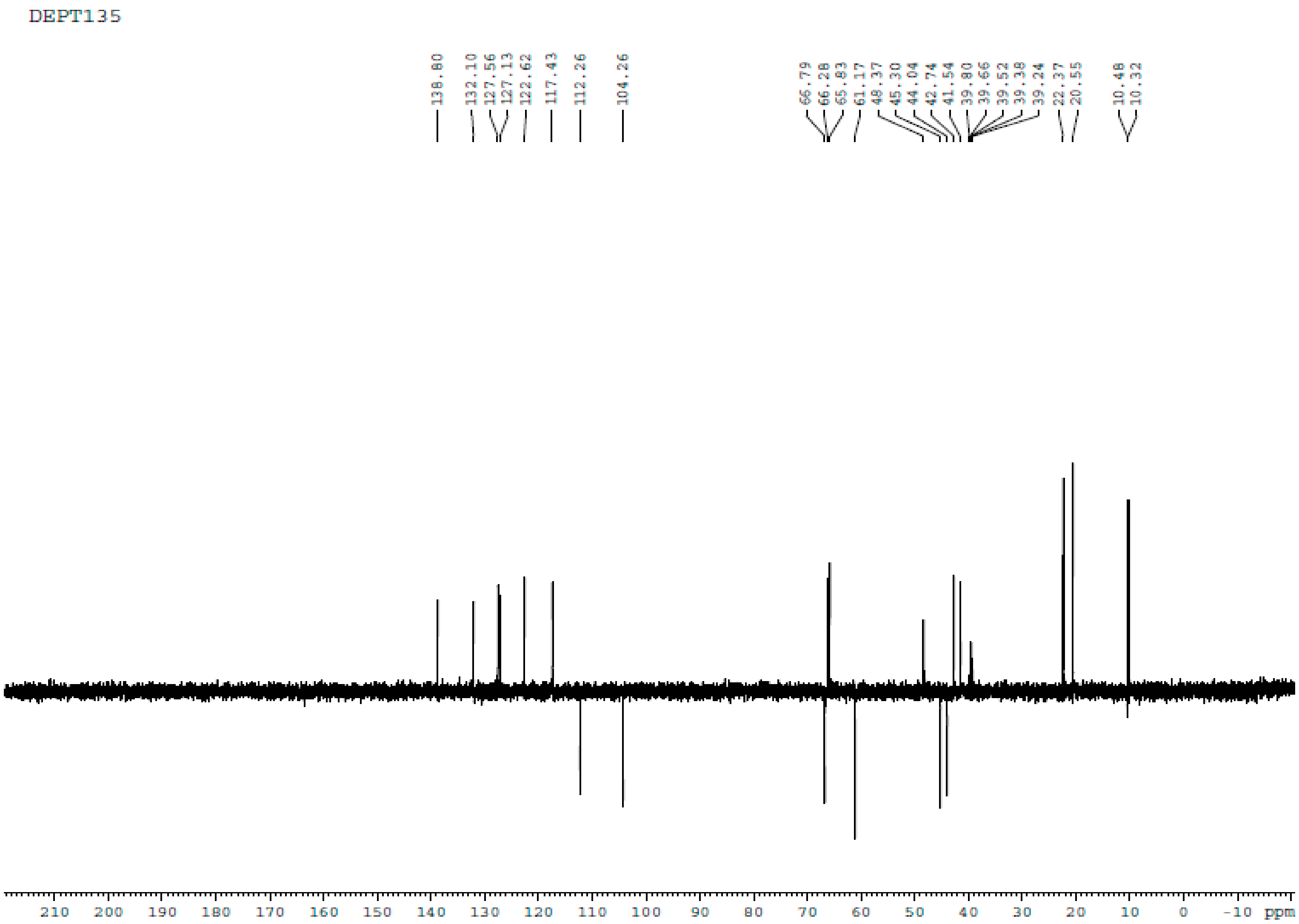

Figure S16. ^{13}C-NMR spectrum and DEPT of **3** in DMSO-*d*$_6$.

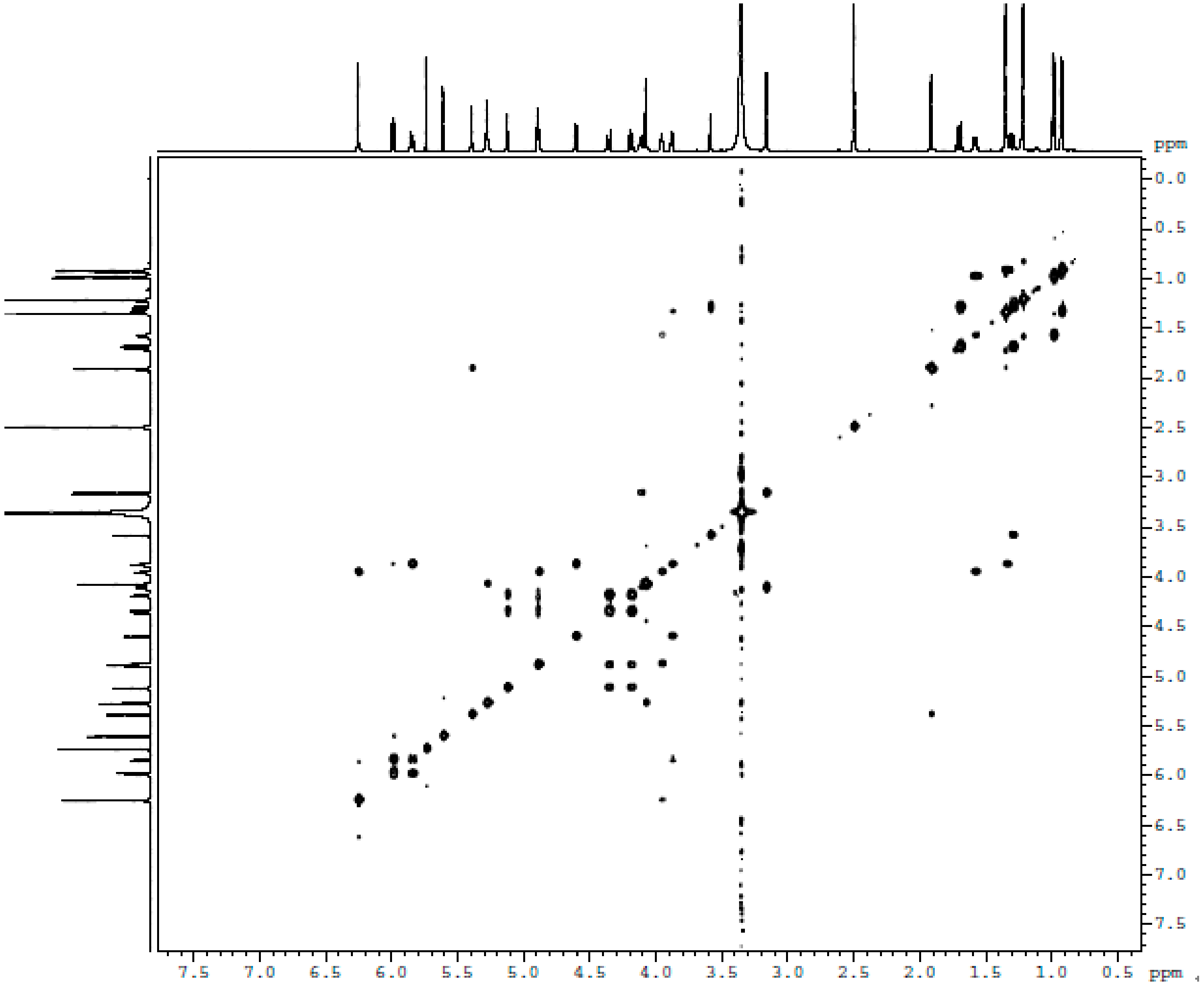

Figure S17. ^{1}H-^{1}H COSY spectrum of **3** in DMSO-d_6.

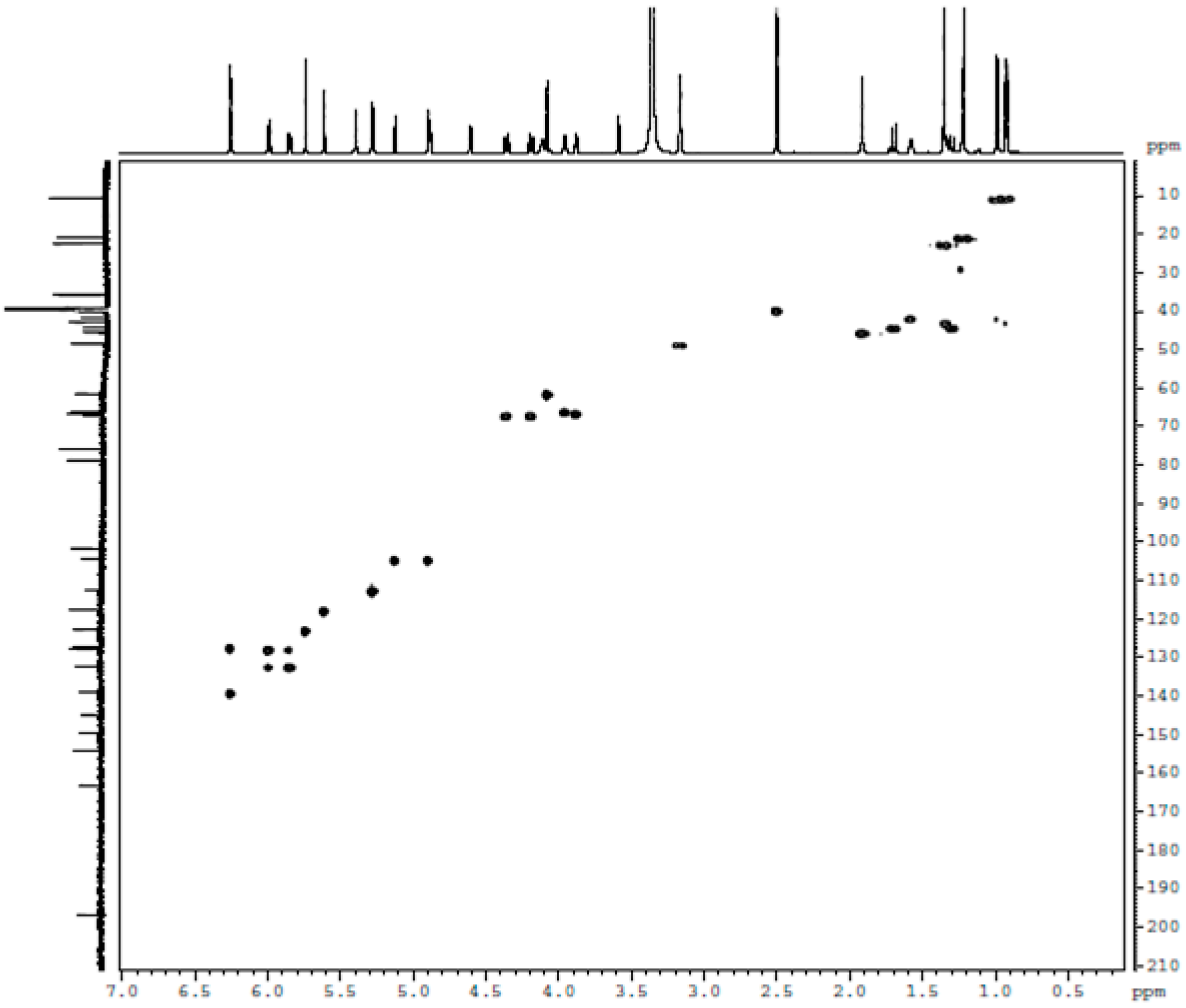

Figure S18. HSQC spectrum of **3** in DMSO-*d*6.

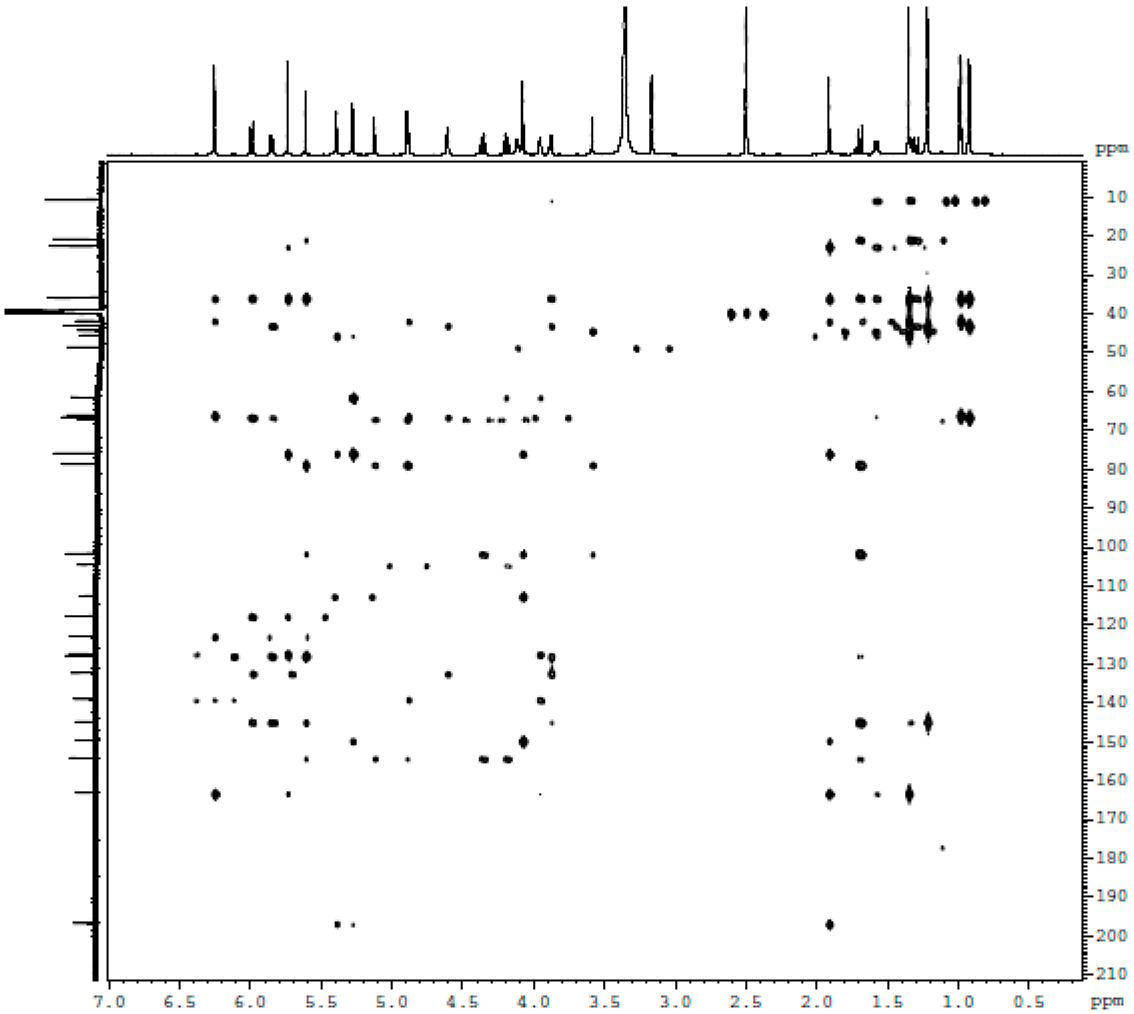

Figure S19. HMBC spectrum of **3** in DMSO-*d*6.

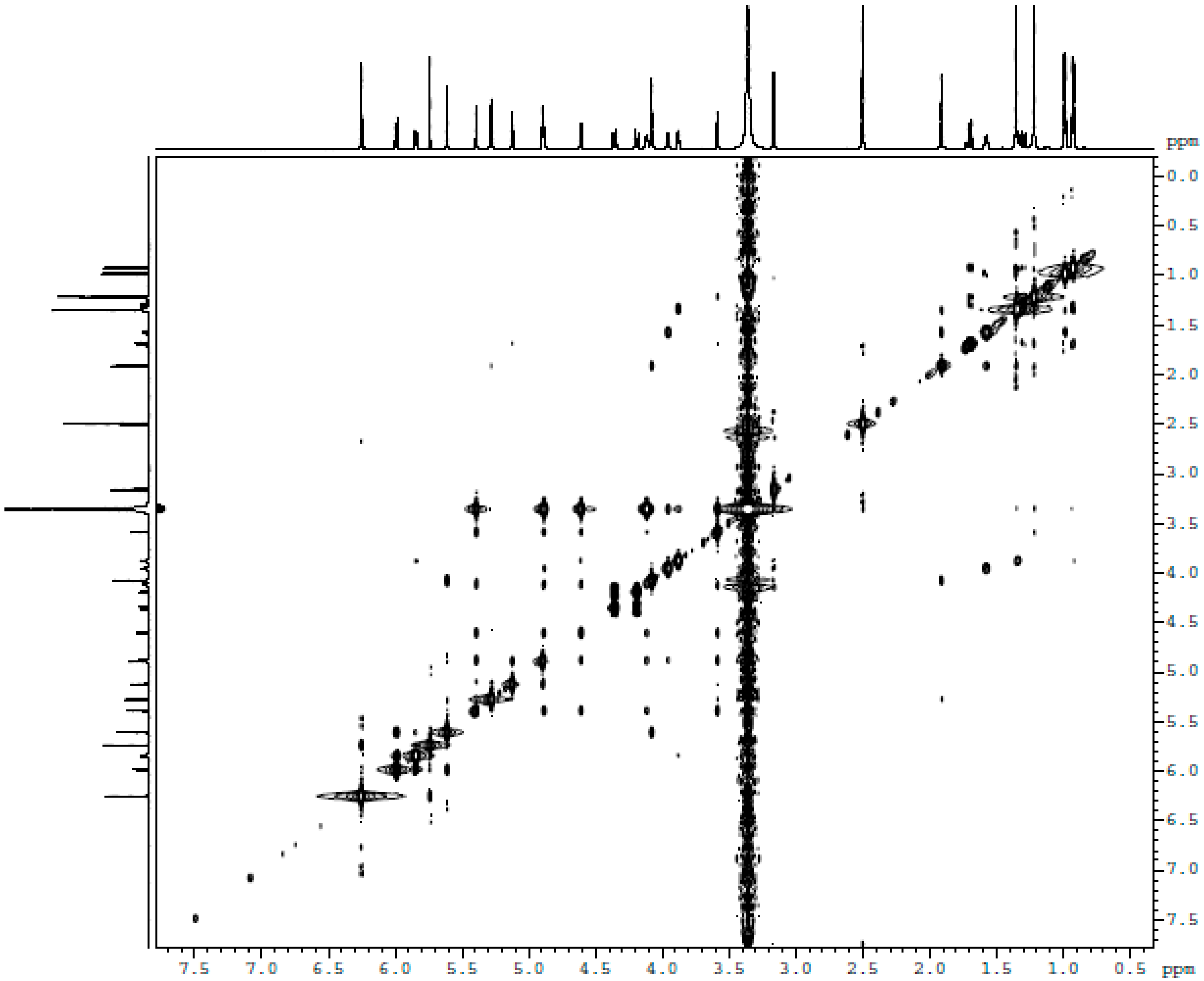

Figure S20. NOESY spectrum of **3** in DMSO-d_6.

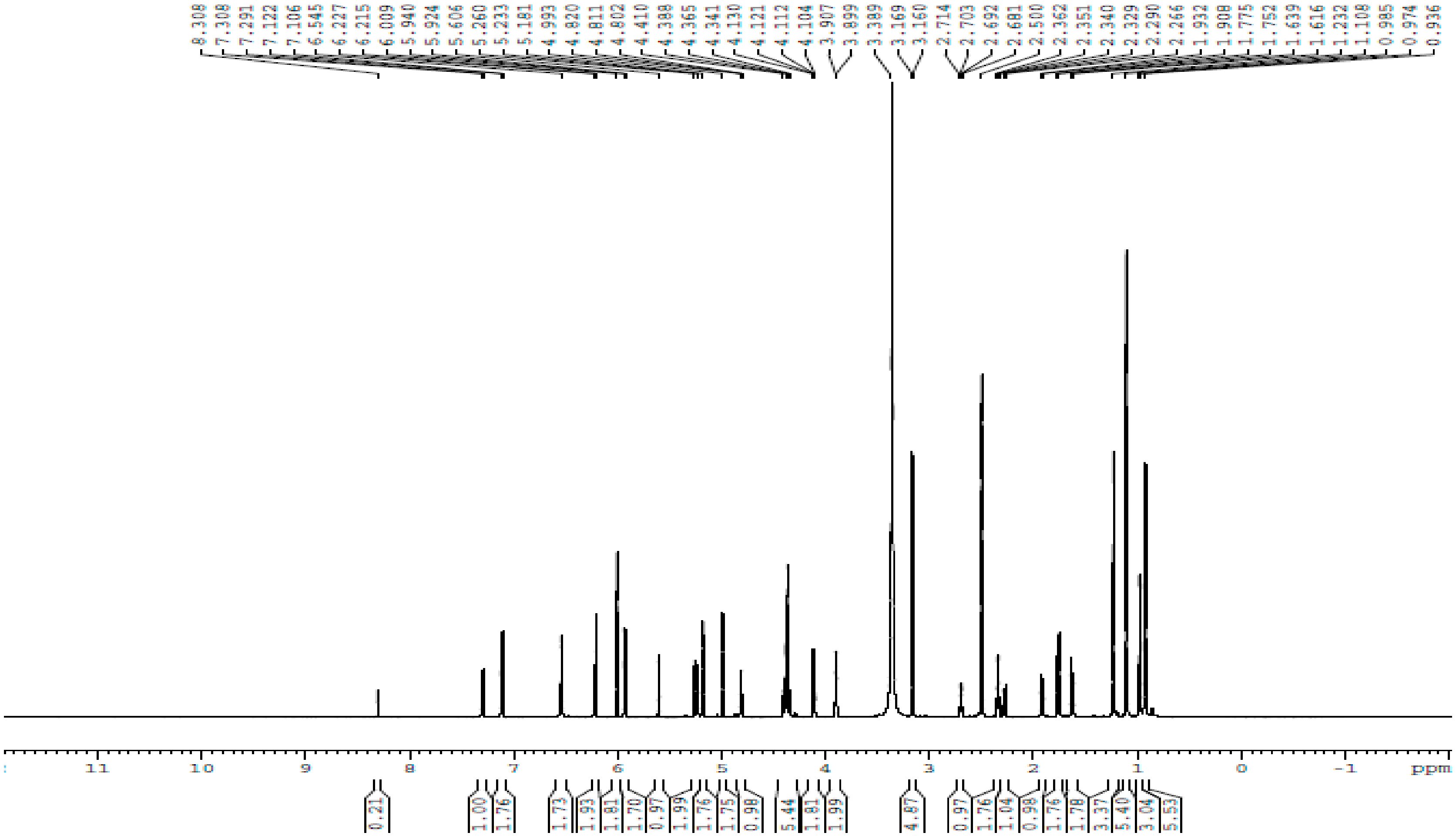

Figure S21. *Cont.*

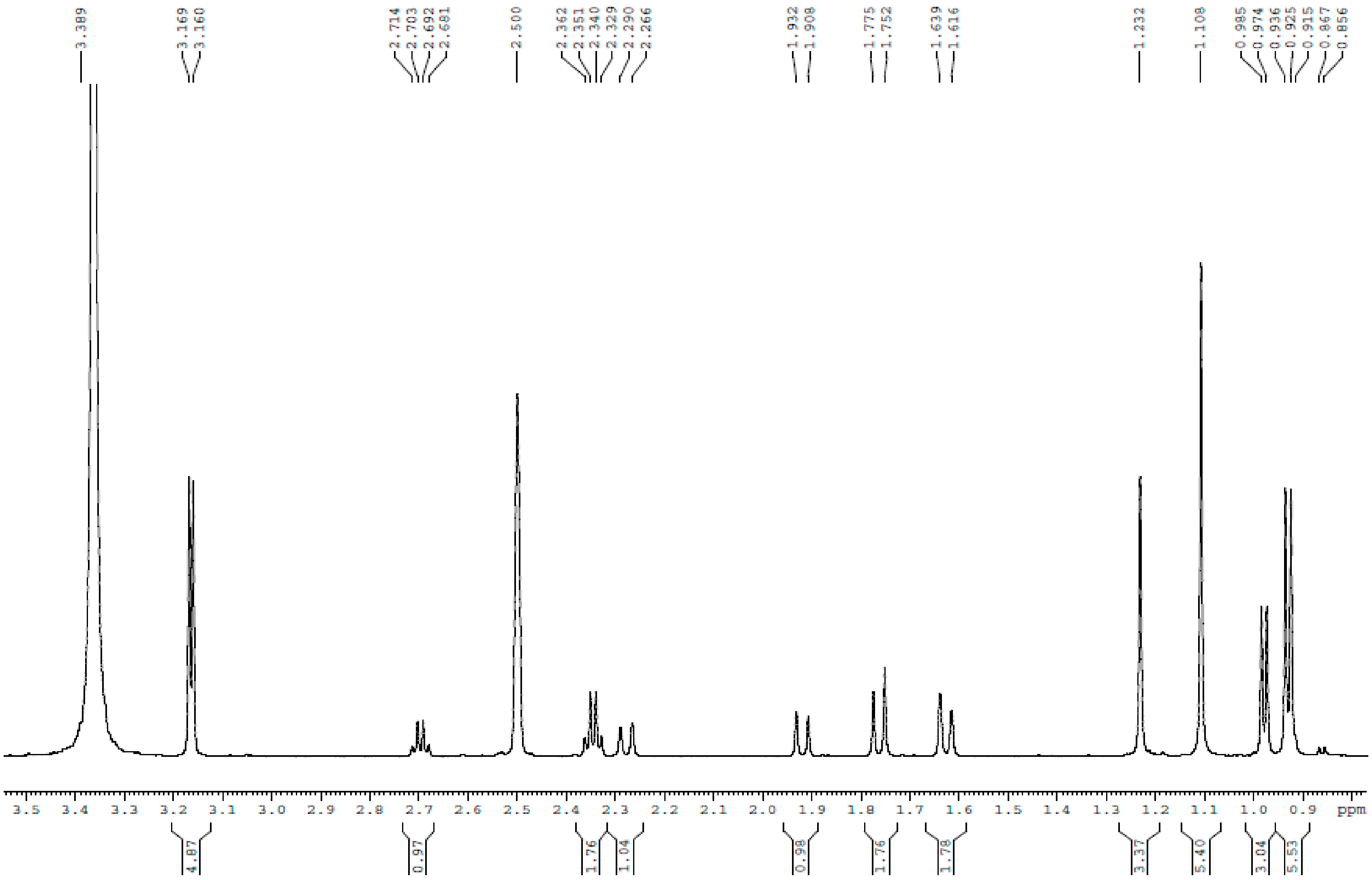

Figure S21. *Cont.*

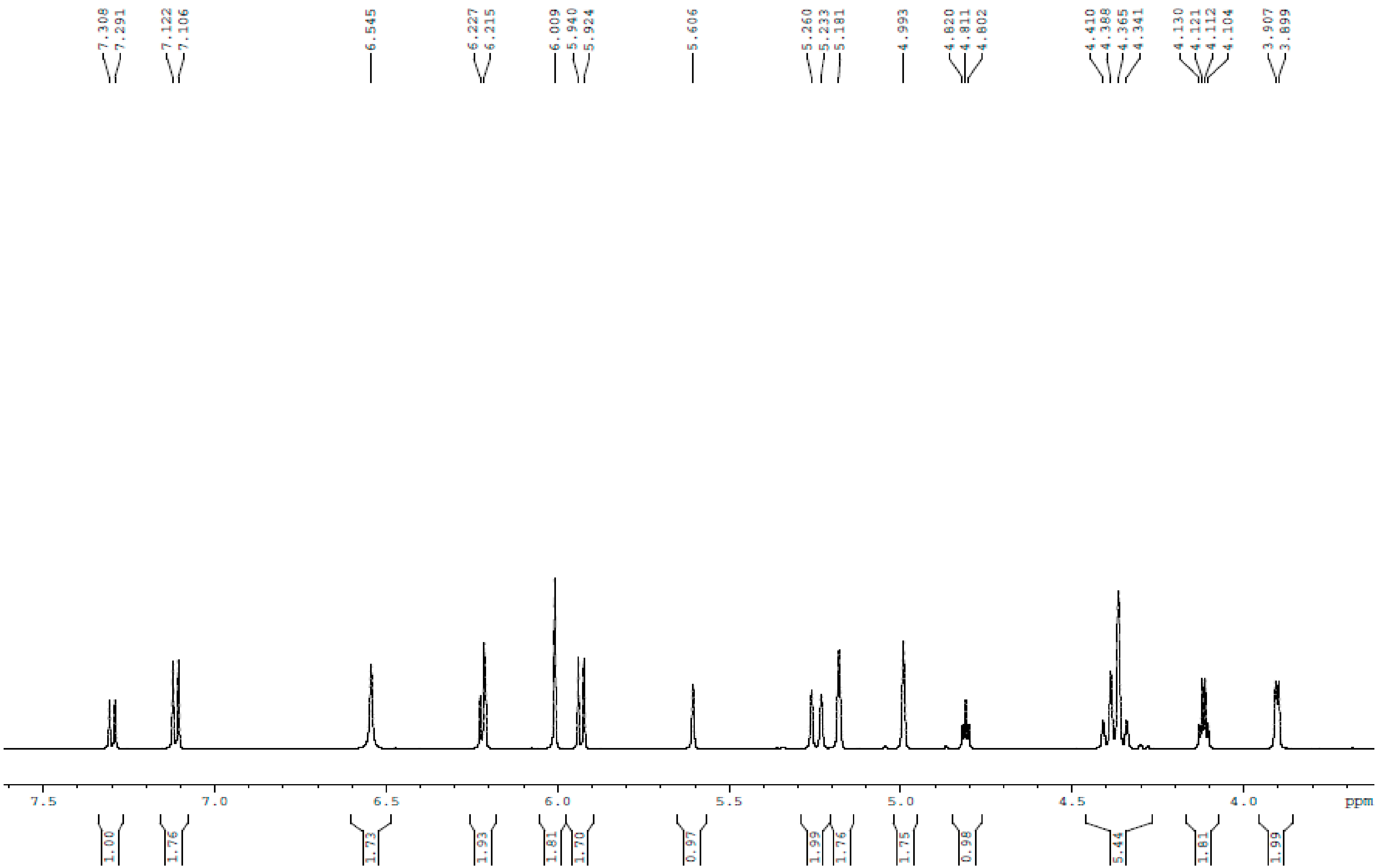

Figure S21. ^{1}H-NMR spectrum of **4** in DMSO-d_6.

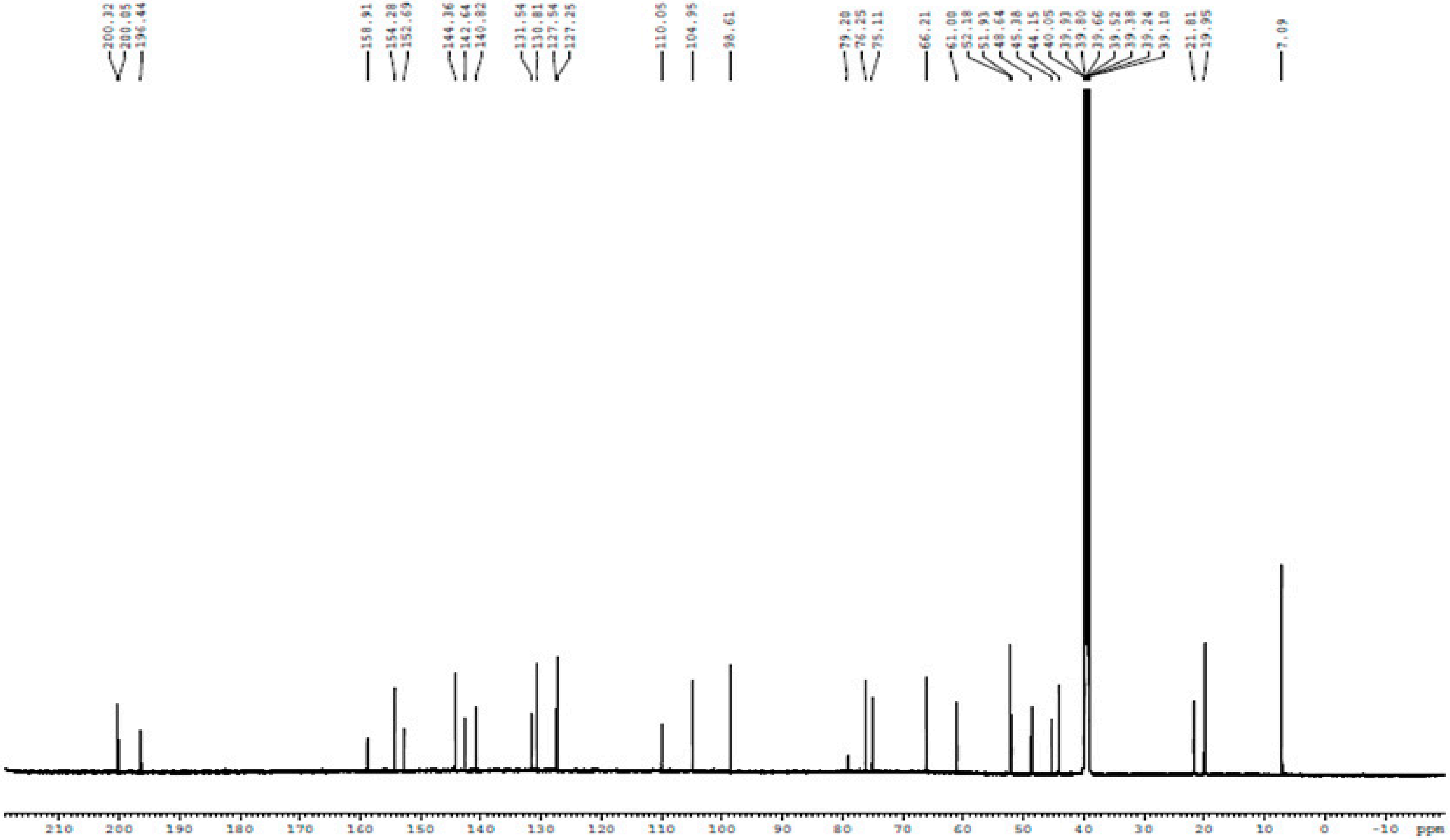

Figure S22. *Cont.*

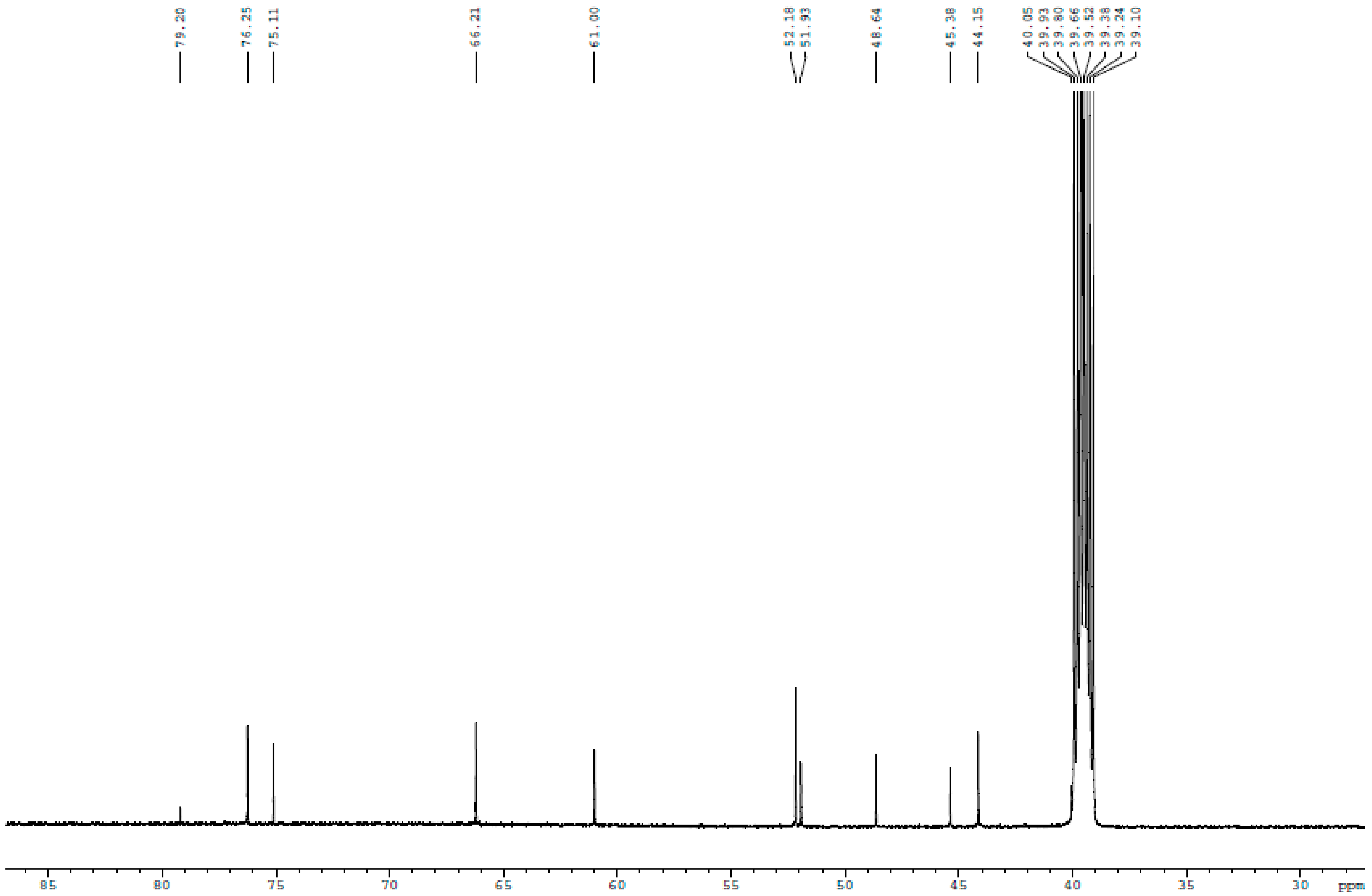

Figure S22. *Cont.*

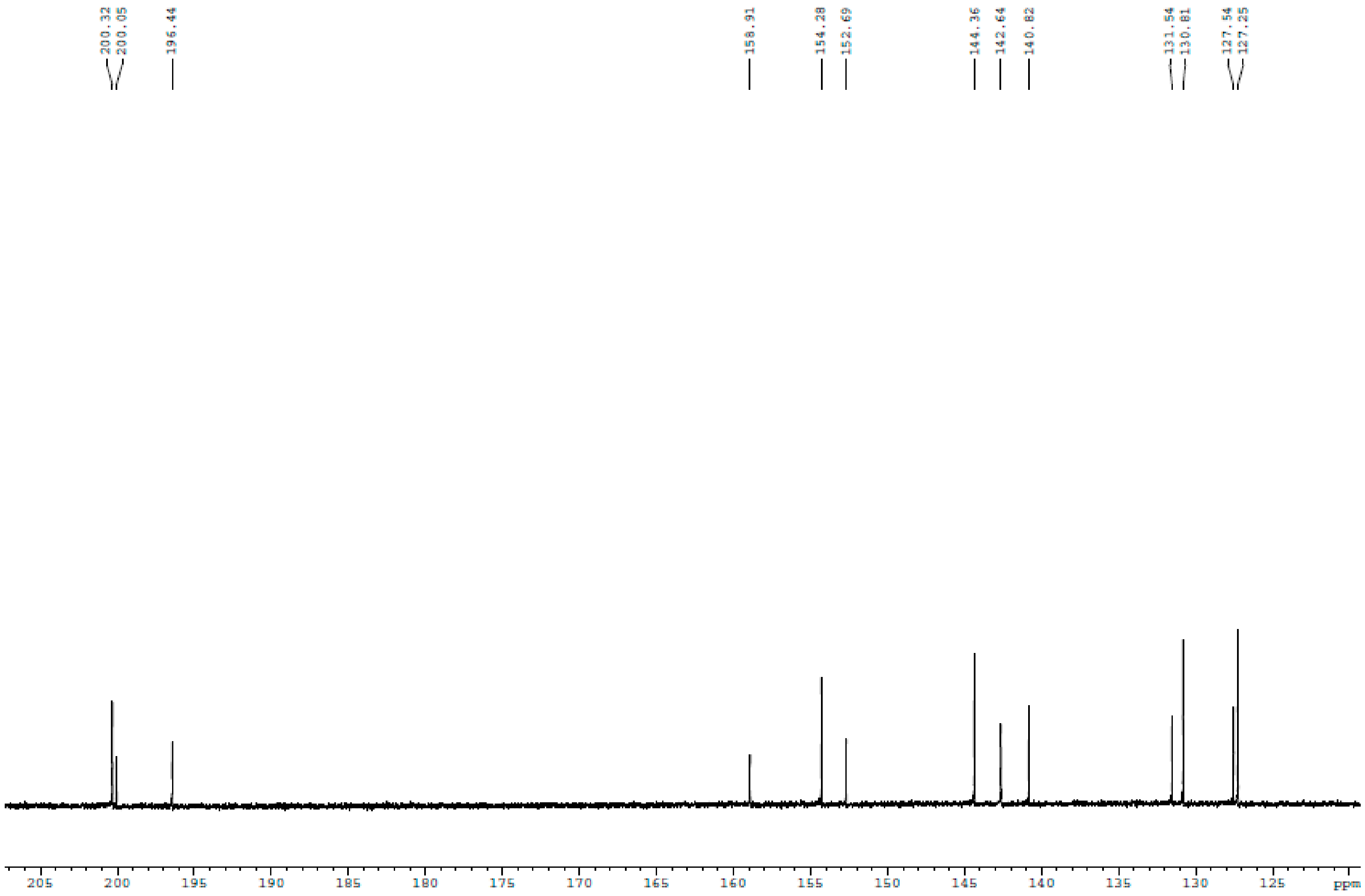

Figure S22. *Cont.*

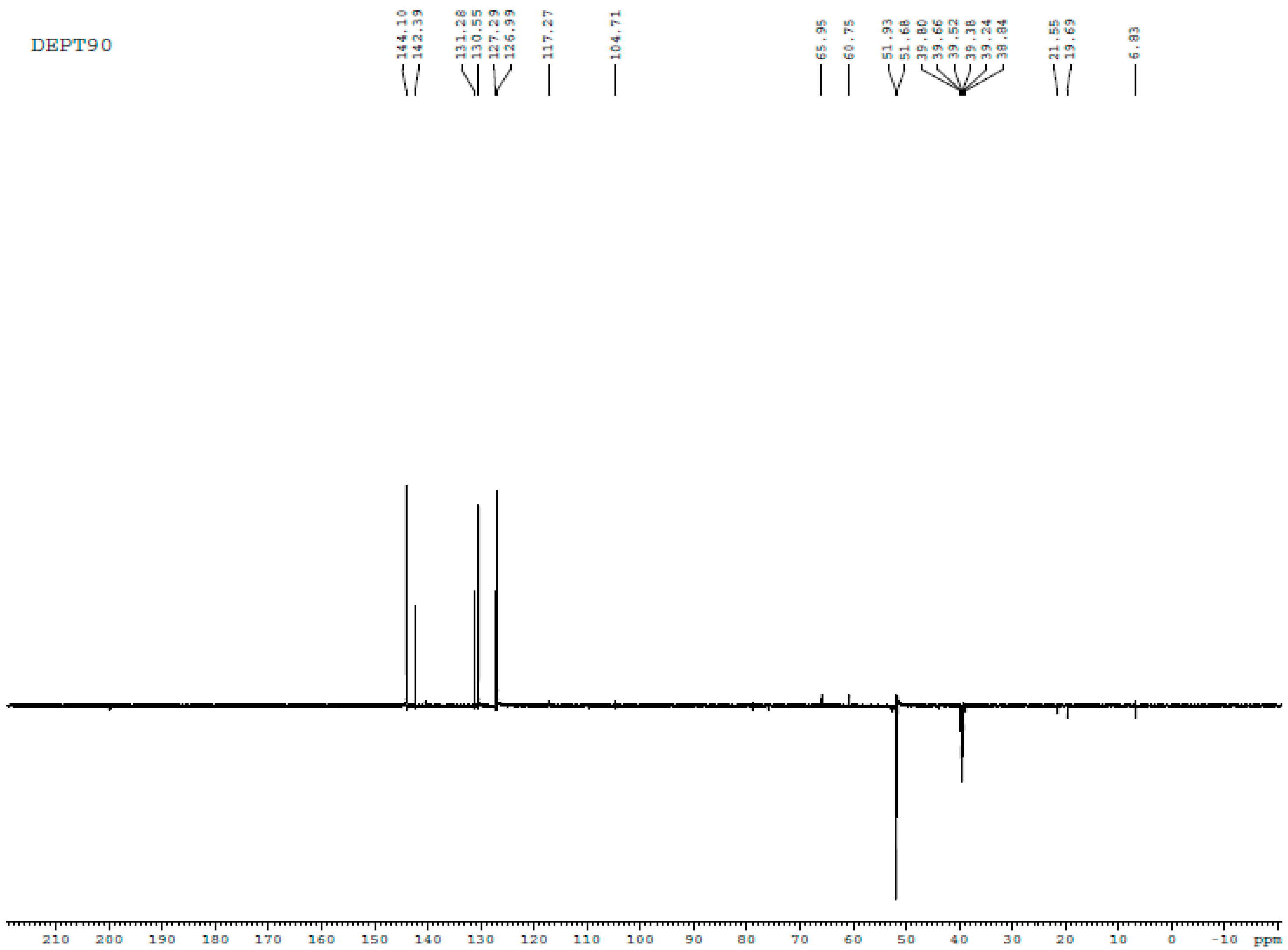

Figure S22. *Cont.*

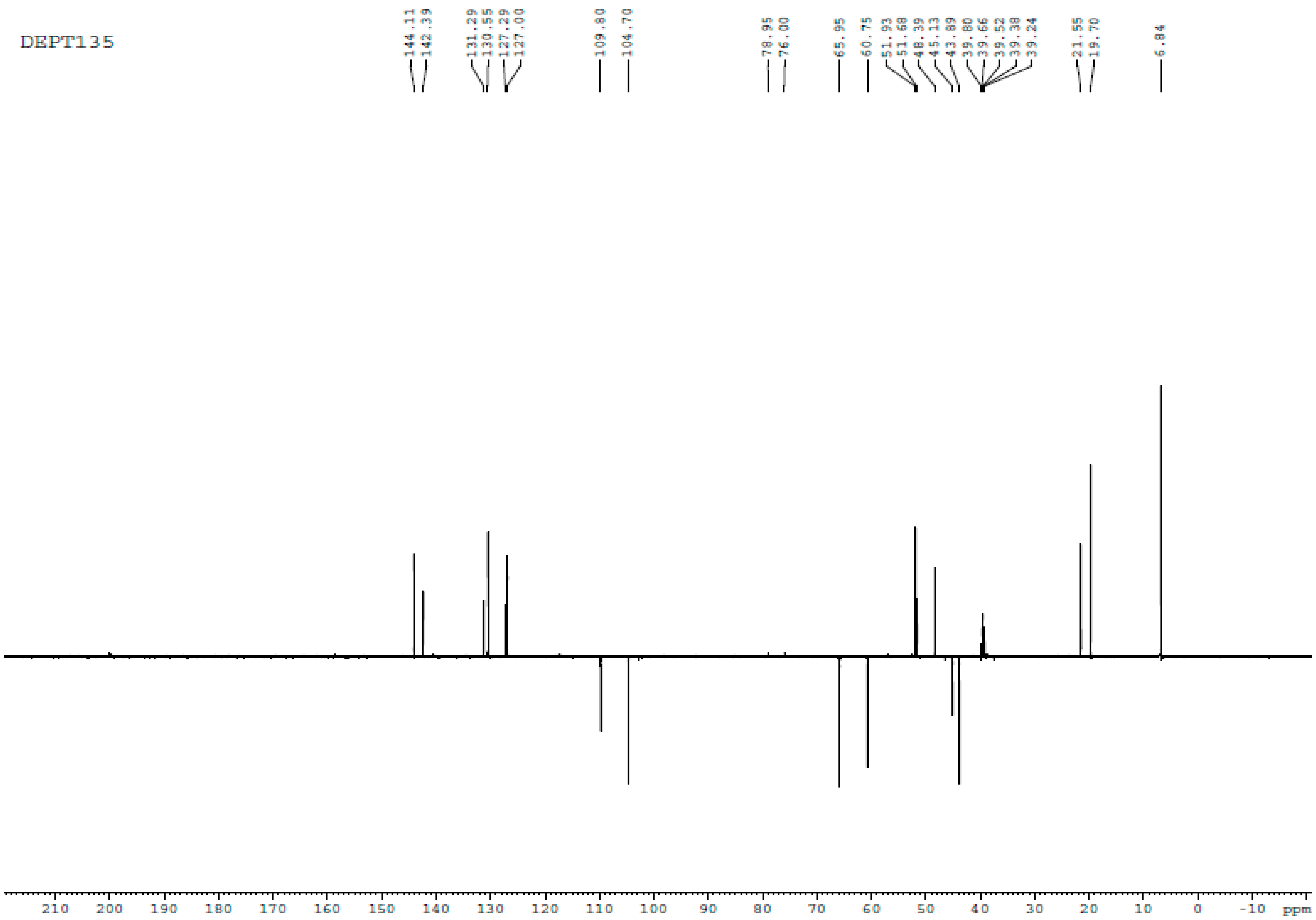

Figure S22. ^{13}C-NMR spectrum and DEPT of **4** in DMSO-d_6.

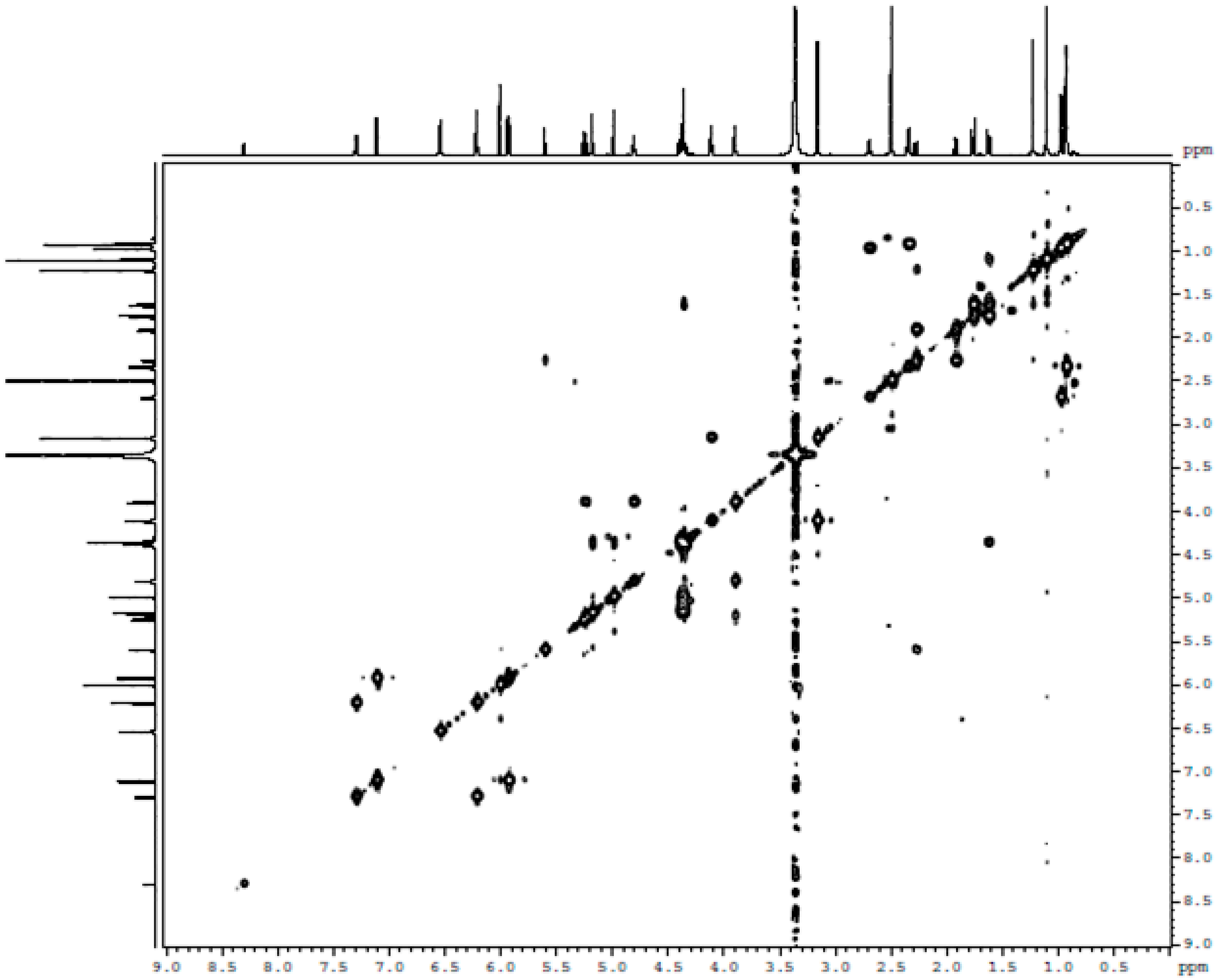

Figure S23. ^{1}H-^{1}H COSY spectrum of **4** in DMSO-d_6.

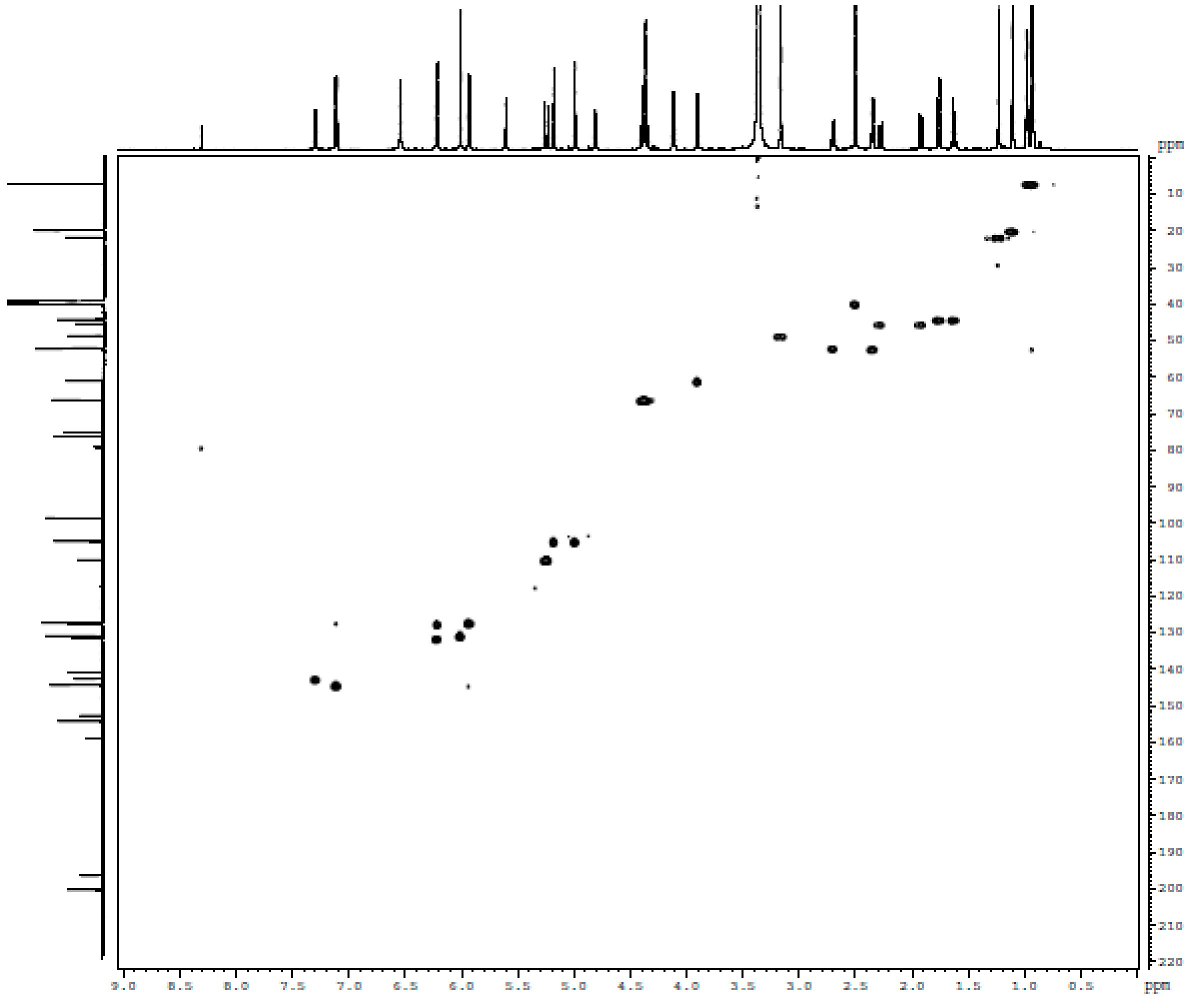

Figure S24. HSQC spectrum of **4** in DMSO-d_6.

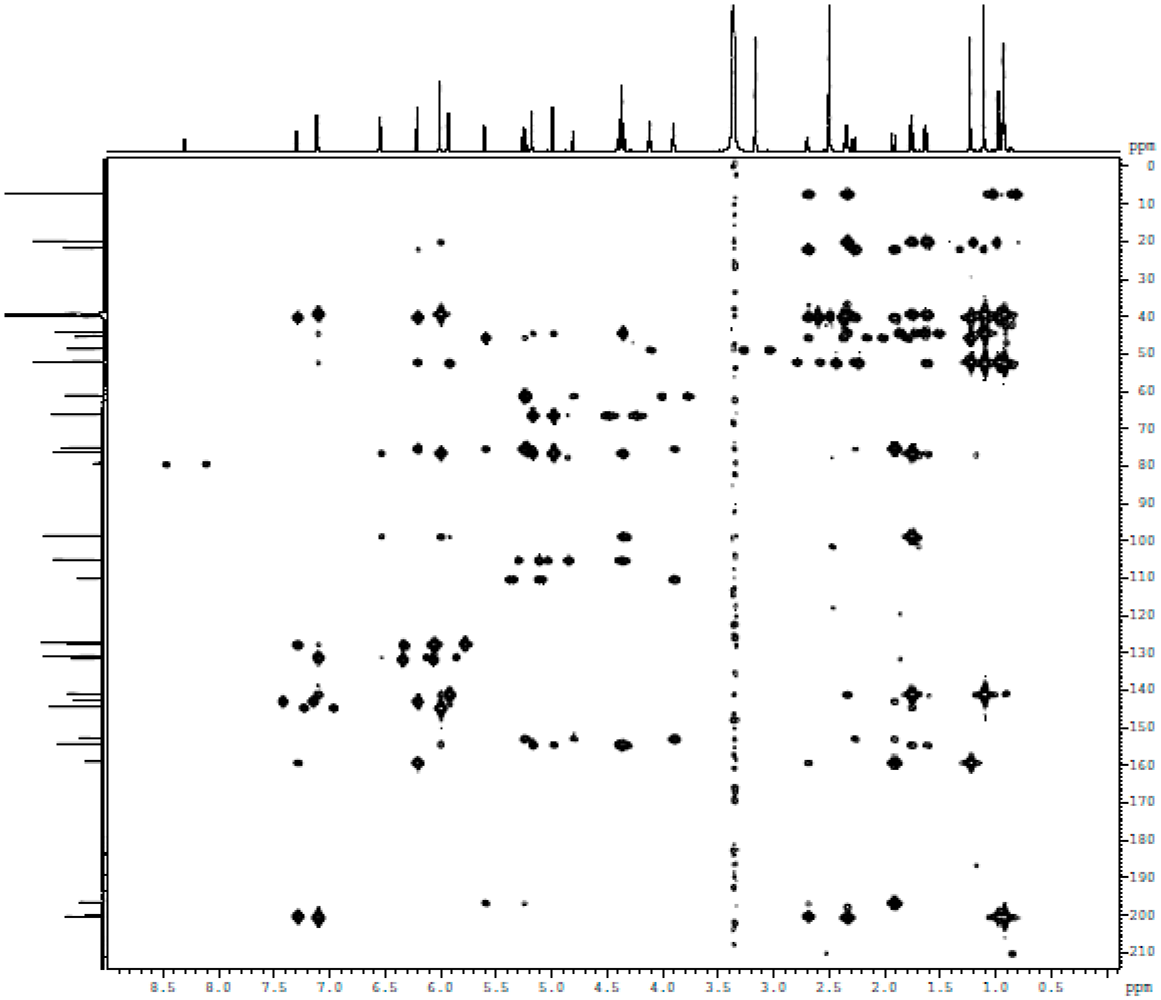

Figure S25. HMBC spectrum of **4** in DMSO-d_6.

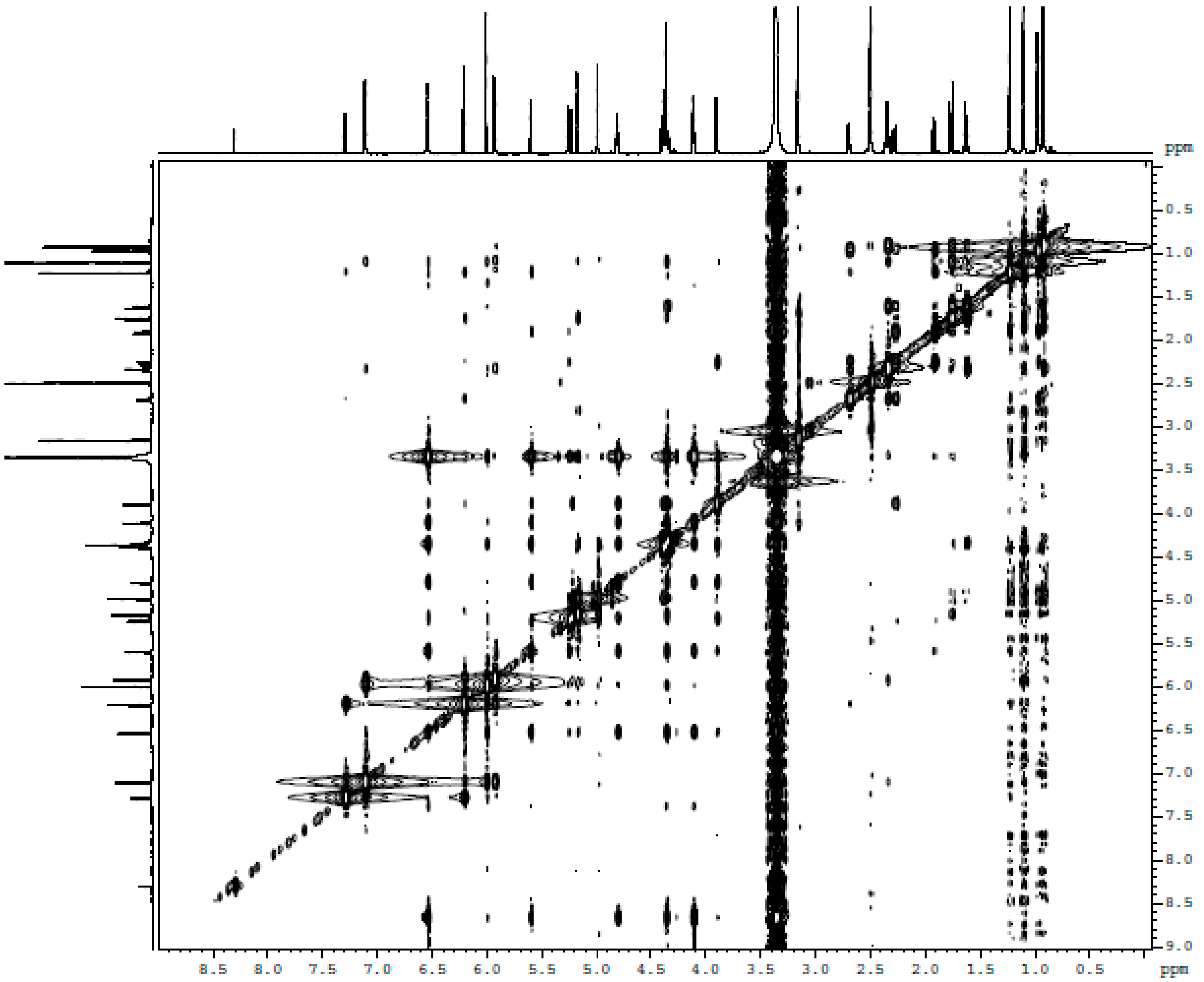

Figure S26. NOESY spectrum of **4** in DMSO-*d*6.

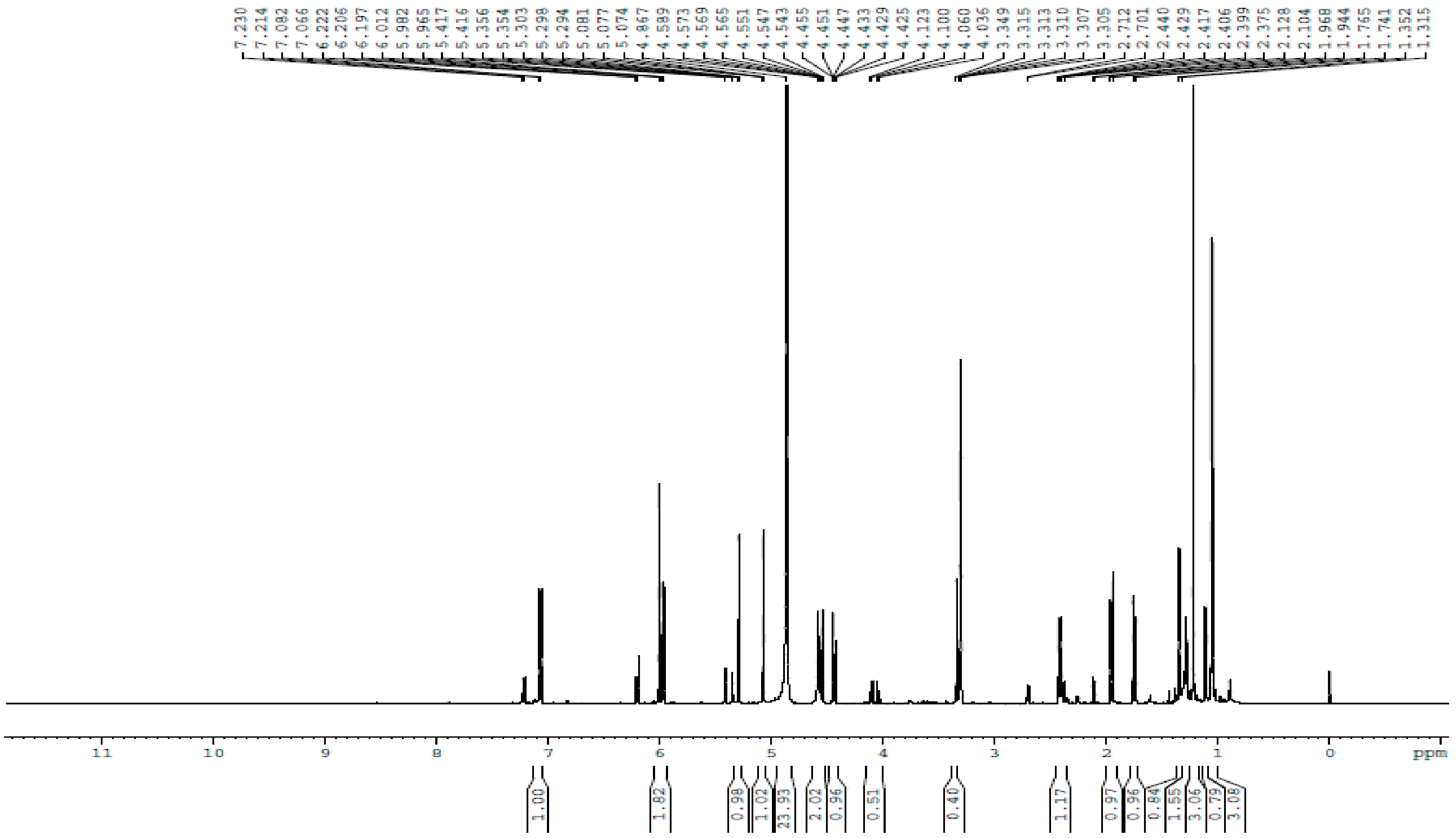

Figure S27. *Cont.*

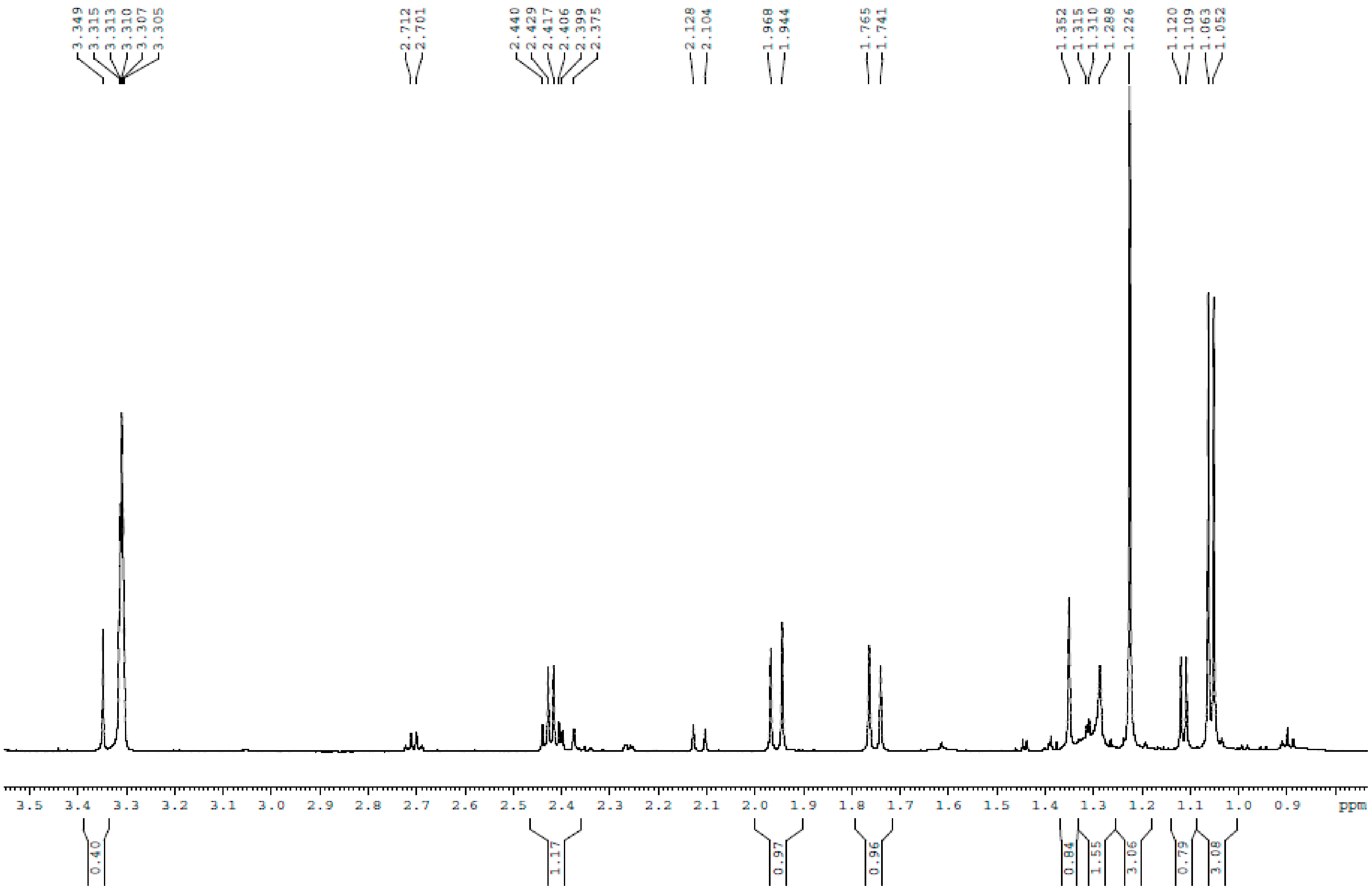

Figure S27. *Cont.*

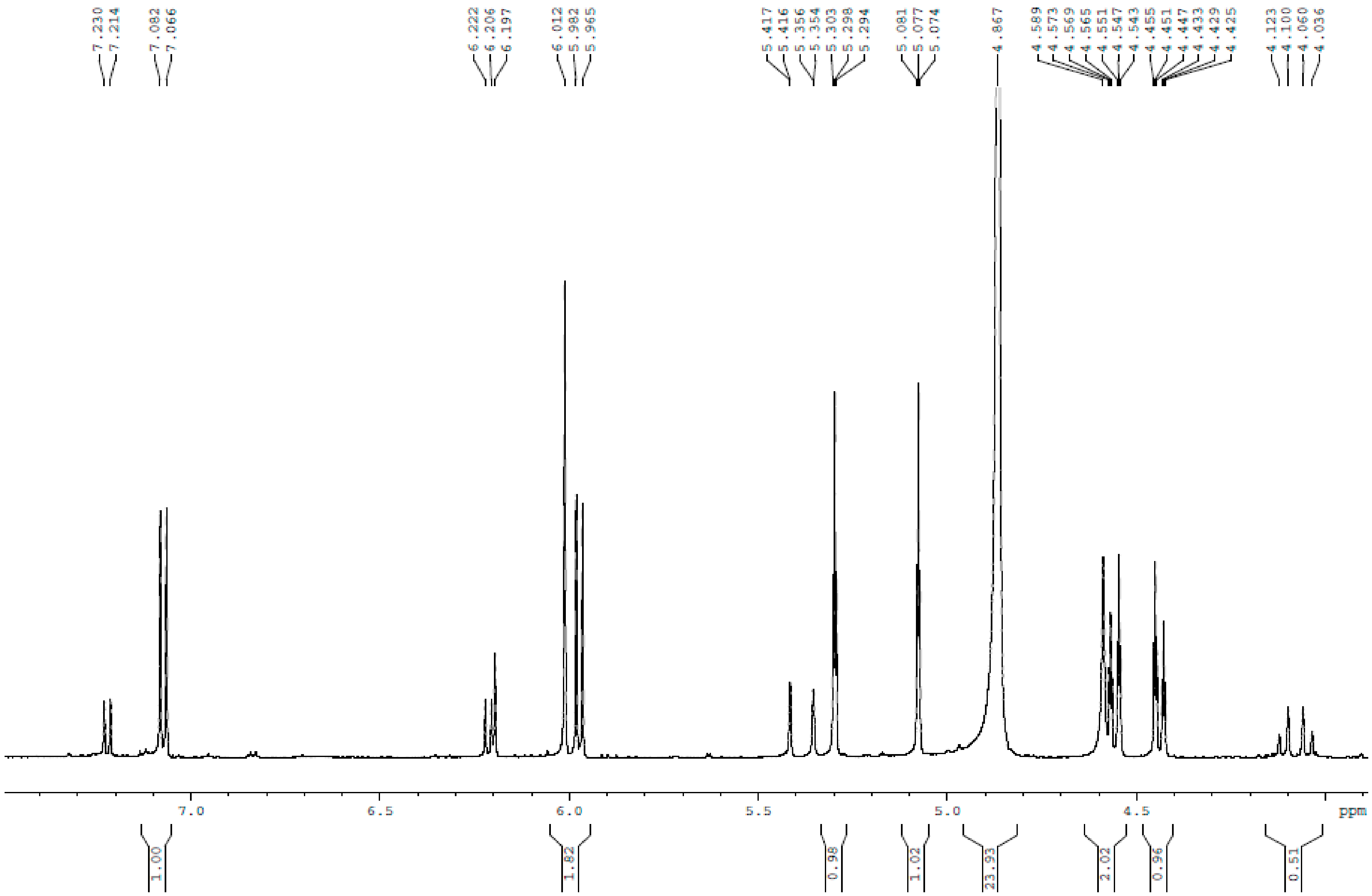

Figure S27. ^{1}H-NMR spectrum of **4** in MEOD-d_4.
